# HSPB1 facilitates chemoresistance through inhibiting ferroptotic cancer cell death and regulating NF-κB signaling pathway in breast cancer

**DOI:** 10.1038/s41419-023-05972-0

**Published:** 2023-07-15

**Authors:** Yiran Liang, Yajie Wang, Yan Zhang, Fangzhou Ye, Dan Luo, Yaming Li, Yuhan Jin, Dianwen Han, Zekun Wang, Bing Chen, Wenjing Zhao, Lijuan Wang, Xi Chen, Tingting Ma, Xiaoli Kong, Qifeng Yang

**Affiliations:** 1 Department of Breast Surgery, Qilu Hospital, Cheeloo College of Medicine, Shandong University, Jinan, 250012 Shandong China; 2 Department of Breast Surgery, Jinan Central Hospital, Cheeloo College of Medicine, Shandong University, Jinan, 250013 Shandong China; 3 Pathology Tissue Bank, Qilu Hospital of Shandong University, Jinan, 250012 Shandong China; 4 Research Institute of Breast Cancer, Shandong University, Jinan, 250012 Shandong China

**Keywords:** Breast cancer, Cancer therapeutic resistance

## Abstract

Chemoresistance is one of the major causes of therapeutic failure and poor prognosis for breast cancer patients, especially for triple-negative breast cancer patients. However, the underlying mechanism remains elusive. Here, we identified novel functional roles of heat shock protein beta-1 (HSPB1), regulating chemoresistance and ferroptotic cell death in breast cancer. Based on TCGA and GEO databases, HSPB1 expression was upregulated in breast cancer tissues and associated with poor prognosis of breast cancer patients, which was considered an independent prognostic factor for breast cancer. Functional assays revealed that HSPB1 could promote cancer growth and metastasis in vitro and in vivo. Furthermore, HSPB1 facilitated doxorubicin (DOX) resistance through protecting breast cancer cells from drug-induced ferroptosis. Mechanistically, HSPB1 could bind with Ikβ-α and promote its ubiquitination-mediated degradation, leading to increased nuclear translocation and activation of NF-κB signaling. In addition, HSPB1 overexpression led to enhanced secretion of IL6, which further facilitated breast cancer progression. These findings revealed that HSPB1 upregulation might be a key driver to progression and chemoresistance through regulating ferroptosis in breast cancer while targeting HSPB1 could be an effective strategy against breast cancer.

## Introduction

Breast cancer is one of the most prevalent malignancies in women, accounting for more than 24% of new female cancer cases and about 15% of cancer-related death around the world [1]. Based on the expression status of estrogen receptor (ER), progesterone receptor (PR), and human epidermal growth factor receptor 2 (HER2), breast cancer is generally classified into four subtypes [2]: Luminal A, Luminal B, HER2-enriched, and triple-negative breast cancer (TNBC). TNBC is a subtype of breast cancer characterized by the absence of ER and PR and the lack of HER2 amplification or overexpression [3], accounting for 15–20% of all invasive breast cancers [4]. Due to the absence of druggable molecular drivers in TNBC, chemotherapy is still the mainstay of systemic treatment for TNBC patients [5]. Nevertheless, intrinsic and acquired drug resistance greatly limited the efficiency of chemotherapy, leading to high rates of metastasis and poor prognosis in breast cancer patients [6]. Therefore, characterization of the underlying molecular mechanisms of chemoresistance would help to develop novel therapeutic strategies to enhance the efficacy of chemotherapy in breast cancer patients.

Numerous mechanisms have been reported to be responsible for chemoresistance [7–9], including enhanced efflux of intracellular drugs, epithelial-to-mesenchymal transition (EMT), improved DNA damage repair, and increased cell death inhibition. Ferroptosis, a recently recognized form of cell death, is mainly caused by excessive iron-dependent lipid peroxidation and results in iron-mediated oxidative damage of cell membranes with an increasing level of intracellular reactive oxygen species (ROS) [10]. It has unique characteristics distinct from apoptosis, necrosis, and autophagy in morphology, biochemistry, and genetics [11, 12]. Accumulated evidence indicated that ferroptosis played a significant role in the fate of cancer cells and response to various cancer treatments, such as chemotherapy, radiotherapy, and immunotherapy [13]. Tumor cells with drug resistance and high metastatic tendency showed higher susceptibility to ferroptosis [14, 15], indicating that targeting the negative regulators of ferroptosis might further render chemoresistant cancer cells susceptible to ferroptotic cell death [16]. In addition, damage-related molecular patterns (DAMPs) released from the ferroptotic cancer cells could enhance inflammation and immune responses through acting on surrounding cells [17–19]. Various reagents, factors, and drugs, such as RSL3, erastin, and sorafenib, have been demonstrated to inhibit tumor growth through inducing ferroptosis [20, 21]. Therefore, further studies are required to elucidate the molecular mechanism of ferroptosis, which would facilitate the implementation of novel approaches to overcome chemoresistance.

Based on the differential analysis of data from TCGA and GEO databases, we identified that the expression of Heat shock protein beta-1 (HSPB1), a member of the human small heat shock proteins (HSPBs), was significantly upregulated in breast cancer tissues. HSPBs family consists of ten isoforms (HSPB1-HSPB10) and is characterized by low molecular weight (MW) (14–43 kD), highly conserved, and capable of dimerization and oligomerization to form large homogeneous or heterogeneous complexes [22, 23]. HSPBs exert different activities in cells: preventing protein aggregation and helping in refolding as holdase chaperones (HSPB2-6, HSPB8); promoting substrates to degradation via autophagy and the ubiquitin-proteasome pathway (HSPB1, HSPB8); regulating the assembly and structural integrity of cytoskeleton components (HSPB1-10); protecting cells against cellular stress and blocking the activity of pro-apoptotic factors (HSPB1-8) [24]. Among them, HSPB1 is one of the most studied members of HSPB family, which has been documented to respond to different types of cellular stress, acting as an antioxidant during oxidative stress and an anti-apoptotic agent during chemical stress [25]. The mutations of HSPBs are mostly associated with multiple diseases, such as cardiomyopathies, myopathies, Charcot-Marie-Tooth disease, and motor neuropathies [26, 27]. According to the TCGA Pan Cancer database (https://www.cbioportal.org/), the mutation rate of HSPB1 in breast cancer patients is about 0.4%, which is lower than that of some classical indicators such as TP53. Interestingly, amplification is the major type of mutation, and it might be responsible for the upregulated expression of HSPB1 in breast cancer. However, the detailed role of HSPB1 mutation in breast cancer has not been reported, and more research is needed in the future.

In the current study, we identified that high expression of HSPB1 was associated with poor prognosis of breast cancer patients. Moreover, functional experiments verified that HSPB1 showed a crucial role in the progression of breast cancer. We further revealed the relevance of HSPB1 in the regulation of chemoresistance of breast cancer for the first time, which was mediated by chemotherapeutics-induced ferroptosis. Our study highlights HSPB1 as a novel regulator of chemoresistance in breast cancer, providing the potential that targeting HSPB1 might be a novel strategy to prevent breast cancer progression and overcome therapy resistance.

## Results

### HSPB1 is elevated in breast cancer tissues, and high HSPB1 expression is associated with poor prognosis of breast cancer patients

**Fig. 1:**
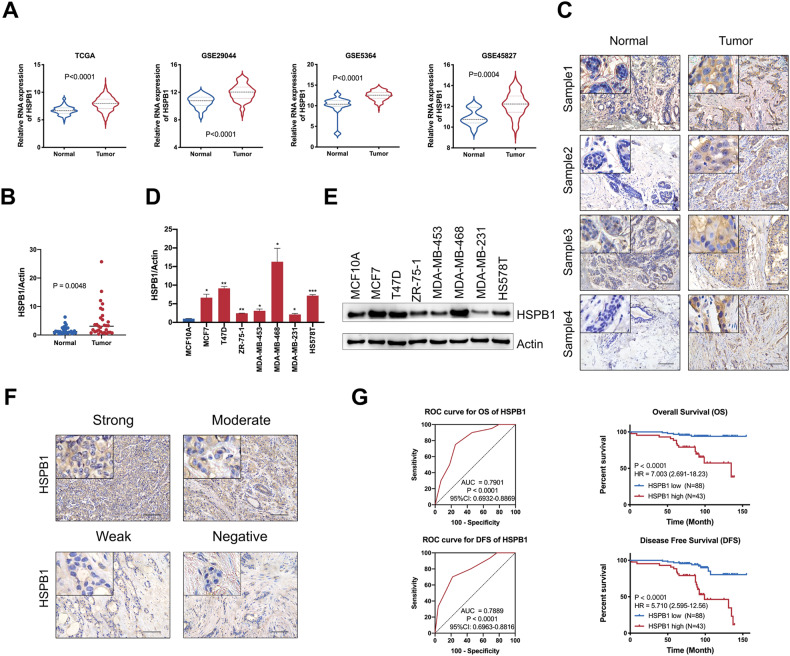
HSPB1 was associated with poor response to chemotherapy of breast cancer patients. **A** The expression of HSPB1 was elevated in breast cancer tissues compared to normal tissues according to TCGA and GEO database. The RNA (**B**) and protein (**C**) expression of HSPB1 was upregulated in breast cancer tissues compared to normal tissues based on Qilu cohort. Scale bar = 100 μm. The RNA (**D**) and protein (**E**) expression of HSPB1 was upregulated in most non-TNBC cells (MCF7, T47D, ZR-75-1, MDA-MB-453) and TNBC cells (MDA-MB-468, MDA-MB-231, HS578T) compared to that in normal cell (MCF-10A). **F** HSPB1 expression was detected by IHC. Scale bar = 100 μm. **G** Patients with breast cancer were divided into two subsets with low and high HSPB1 expression based on ROC analysis. The correlation between HSPB1 expression and overall survival or disease-free survival in breast cancer was calculated with the log-rank test. (*P < 0.05, **P < 0.01, ***P < 0.001).

We first analyzed the mRNA expression in several public breast cancer datasets, and hierarchical clustering analysis revealed that the expression of HSPB1 was upregulated in breast cancer tissues compared to normal tissues based on TCGA and GEO databases (Fig. 1A, Supplementary Fig. S1A). Moreover, higher RNA expression of HSPB1 was discovered in several other cancers (Supplementary Fig. S1B), such as cervical and endocervical cancer (CESC), cholangiocarcinoma (CHOL), esophageal carcinoma (ESCA), glioblastoma (GBM), kidney renal papillary cell carcinoma (KIRP), and liver hepatocellular carcinoma (LIHC). We further detected the expression of HSPB1 in human breast cancer tissues and adjacent normal tissues in a cohort of patients from Qilu Hospital, and upregulated mRNA expression of HSPB1 was also detected in breast cancer tissues (Fig. 1B). Consistently, the IHC staining also revealed that the protein expression of HSPB1 was upregulated in breast cancer tissues compared to normal tissues according to Qilu cohort and TCGA dataset (Fig. 1C, Supplementary Fig. S1C). In addition, HSPB1 expression was more abundant in most breast cancer cells compared to normal cell (MCF-10A) (Fig. 1D, E), further supporting the tumor-promoting role of HSPB1 in breast cancer. We also investigate the clinical significance of HSPB1, and noted that elevated HSPB1 expression was associated with worse overall survival (OS), recurrence-free survival (RFS), and distant metastasis free survival (DMFS) by analyzing several publicly available datasets (Supplementary Fig. S1D–F). Furthermore, Kaplan–Meier analysis showed that breast cancer patients with high HSPB1 expression had poorer overall survival and disease-free survival based on the analysis of 131 cases of breast cancers (Fig. 1F, G). Collectively, these findings indicated that HSPB1 expression was increased in breast cancer tissues and associated with poor prognosis of breast cancer patients.

### Clinical significance of HSPB1 in breast cancer

The chi-square test was used to evaluate the correlations between HSPB1 expression and clinicopathologic features of breast cancer patients, such as age, tumor stage, LN metastasis, distant metastasis, histologic grade, ER status, PR status, HER-2 status, and Ki67 expression. The results indicated that high expression of HSPB1 was significantly associated with distant metastasis in breast cancer (*p* < 0.001), indicating a potential role of HSPB1 in breast cancer progression (Supplementary Table S1). Furthermore, univariate and multivariate Cox regression analyses were performed to screen the potential prognostic factors in breast cancer. We found that age, >3 LN metastasis, and HSPB1 expression were independent prognostic indicators for the overall survival of breast cancer (Supplementary Table S2). Significantly, age, LN metastasis, distant metastasis, and HSPB1 expression were considered independent prognostic indicators for disease-free survival of breast cancer (Supplementary Table S3). These results indicated that HSPB1 was an unfavorable prognostic biomarker in breast cancer.

### HSPB1 overexpression facilitates breast cancer cell proliferation, migration, and invasion in vitro

**Fig. 2:**
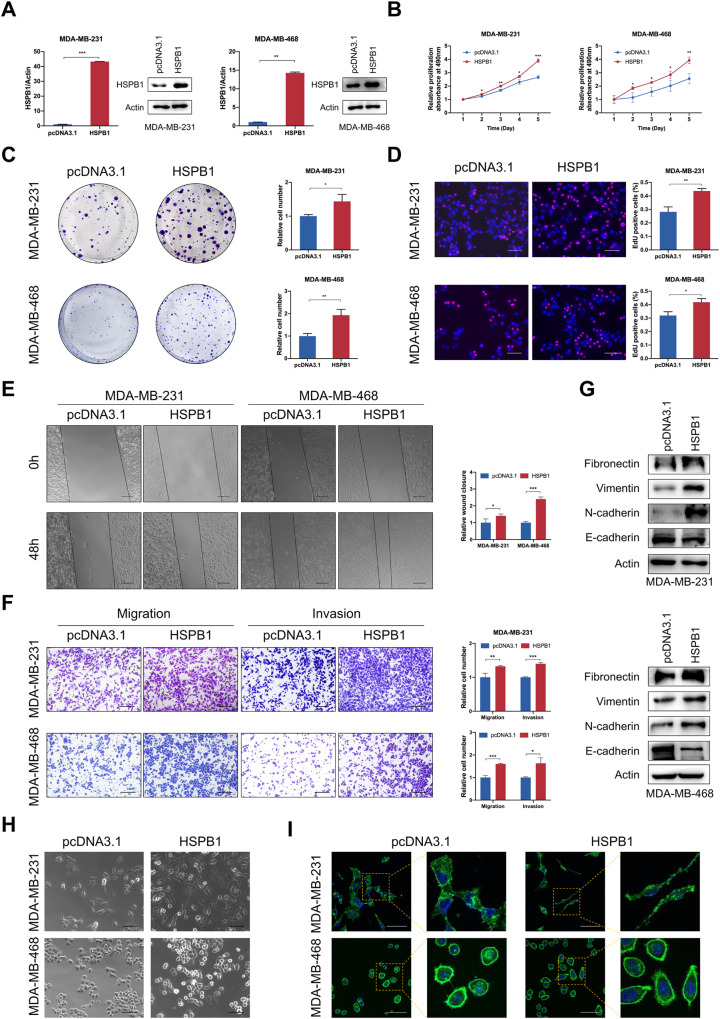
HSPB1 overexpression promoted breast cancer growth, migration, and invasion in vitro. **A** HSPB1 overexpression efficiency was confirmed by qRT-PCR and western blot in breast cancer cells. MTT (**B**), colony formation (**C**), and EdU (**D**) assays were performed to evaluate the effect of HSPB1 overexpression on cell proliferative ability. Scale bar = 100 μm. **E** Wound healing assay was used to evaluate the effect of HSPB1 overexpression on the migration ability of breast cancer cells. Scale bar = 200 μm. **F** The migratory and invasive abilities of HSPB1 overexpressing breast cancer cells were assessed by transwell assay. Scale bar = 100 μm. **G** Western blot showed the effect of HSPB1 on the expression of EMT-related proteins. **H** Representative images of morphological changes of control and HSPB1 overexpressing breast cancer cells. Scale bar = 100 μm. **I** Morphological changes of control and HSPB1 overexpressing breast cancer cells assessed by phalloidin staining. Scale bar = 75 μm. (*P < 0.05, **P < 0.01, ***P < 0.001).

To investigate whether HSPB1 can alter breast cancer cell tumor biology, a series of in vitro experiments were performed in MDA-MB-231 and MDA-MB-468 cell lines overexpressing HSPB1 or with HSPB1 knockdown. Overexpression efficiency was confirmed by qRT-PCR and western blot (Fig. 2A). The MTT and colony formation assays indicated that HSPB1 overexpression promoted breast cancer cell proliferation (Fig. 2B, C). Consequently, HSPB1 overexpression led to increased DNA synthesis activities as determined by EdU assays (Fig. 2D). As shown in the wound healing assay and transwell assay, HSPB1 overexpression markedly promoted the migration and invasion abilities of both MDA-MB-231 and MDA-MB-468 cells (Fig. 2E, F). The epithelial-mesenchymal transition (EMT) is the major mechanism of migration and invasion of cancer cells. Therefore, we further assessed the role of HSPB1 on the expression of EMT markers. Western blot assay (Fig. 2G) revealed that HSPB1 overexpression led to decreased expression of epithelial markers (E-cadherin) and increased expression of mesenchymal markers (Fibronectin, N-cadherin, Vimentin), highlighting the significant effect of HSPB1 on regulating EMT in breast cancer cells. In addition, we found that HSPB1 overexpression could lead to obvious morphological changes, which shaped from cobblestone to fibroblast-like morphology (Fig. 2H, I). Consistently, HSPB1 knockdown inhibited cell growth, migration, invasion, and EMT in breast cancer cells (Supplementary Fig. S2). Collectively, these results indicated that HSPB1 acted as a tumor promoter in breast cancer cells.

### HSPB1 overexpression suppresses ferroptosis to promote doxorubicin resistance of breast cancer cells in vitro

**Fig. 3:**
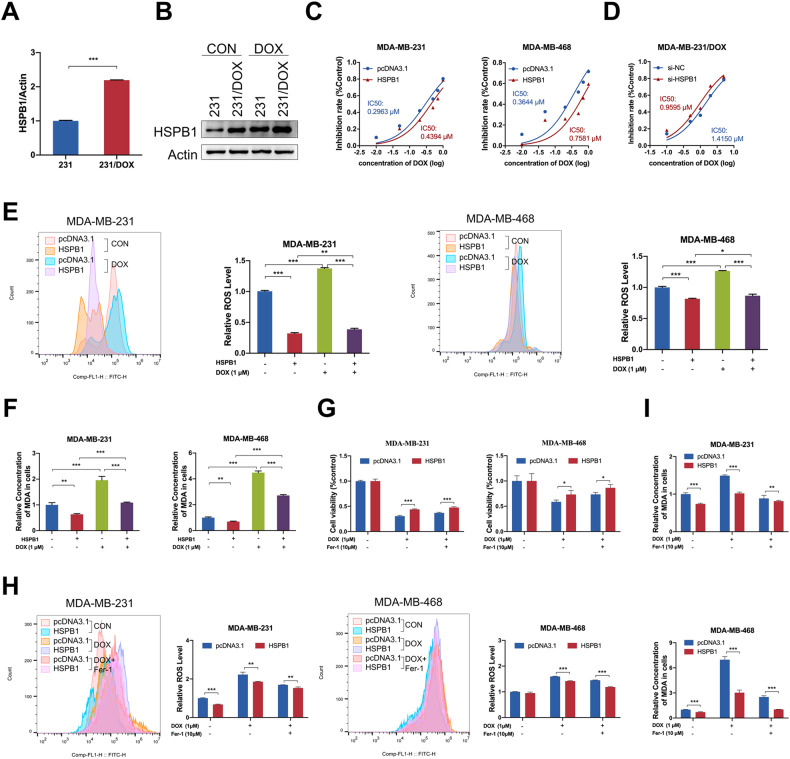
HSPB1 overexpression suppressed doxorubicin-induced ferroptosis in breast cancer cells. The RNA (**A**) and protein (**B**) expression of HSPB1 was elevated in doxorubicin-resistant breast cancer cells. **C** Inhibitory rates of MDA-MB-231 and MDA-MB-468 cells with or without HSPB1 overexpression was analyzed by MTT assay 48 h after treatment with different concentrations of doxorubicin. **D** Inhibitory rates of MDA-MB-231/DOX cells with or without HSPB1 knockdown was analyzed by MTT assay 48 h after treatment with different concentrations of doxorubicin. ROS levels (**E**) and cellular MDA levels (**F**) were determined in MDA-MB-231 and MDA-MB-468 cells with or without HSPB1 overexpression 48 h after treatment with 1 μM doxorubicin. **G** Cell viability was examined in MDA-MB-231 and MDA-MB-468 cells with or without HSPB1 overexpression 48 h after treatment with doxorubicin (1 μM) plus either DMSO or Ferrostatin-1 (Fer-1, 10 μM). ROS levels (**H**) and cellular MDA levels (**I**) were determined in MDA-MB-231 and MDA-MB-468 cells with or without HSPB1 overexpression 48 h after treatment with 1 μM doxorubicin plus either DMSO or 10 μM Fer-1. (*P < 0.05, **P < 0.01, ***P < 0.001).

Elevated mRNA and protein expression levels of HSPB1 were identified in doxorubicin-resistant breast cancer cells (MDA-MB-231/DOX, 231/DOX) compared with parental cells (MDA-MB-231, 231) (Fig. 3A, B), indicating its promoting role in doxorubicin resistance. Moreover, doxorubicin treatment led to increased HSPB1 expression in a dose-dependent and time-dependent manner (Supplementary Fig. S3A, B). We also analyzed the expression level of HSPB1 in breast cancer tissues with different responses to chemotherapy using several public breast cancer datasets, and the results showed that HSPB1 mRNA is upregulated in the majority of chemo-resistant breast cancer tissues (Supplementary Fig. S3C). These results highlighted the significant association between HSPB1 expression and doxorubicin resistance. Therefore, we further evaluated the effect of HSPB1 on breast cancer cell resistance to doxorubicin. Significantly, the IC_50_ value of doxorubicin was significantly higher in HSPB1 overexpressing breast cancer cells compared to that in control cells (Fig. 3C). Consistently, breast cancer cells transfected with si-HSPB1 showed attenuated resistance to doxorubicin compared to cells transfected with si-NC (Fig. 3D, Supplementary Fig. S4A). Moreover, HSPB1 knockdown could lead to decreased cell proliferation, migration, and invasion of doxorubicin-resistant cells (Supplementary Fig. S4B–G). These results revealed the essential role of HSPB1 in doxorubicin resistance of breast cancer.

Recently, HSPB1 is identified as a novel negative regulator of ferroptosis in several human cancer cells [28], such as Hela, U2OS, and LNCap cells. Our results indicated that erastin (an inducer of ferroptosis) treatment led to increased mRNA and protein levels of HSPB1 in a dose-dependent manner in breast cancer cells (Supplementary Fig. S5A), which is consistent with the observations in other cancer cells [28]. HSPB1 overexpression inhibited while HSPB1 knockdown increased erastin-induced intracellular concentrations of lipid ROS in breast cancer cells (Supplementary Fig. S5B, C), which is one of the significant signatures of ferroptosis. Furthermore, we treated breast cancer cells with ferrostatin-1 (Fer-1, an inhibitor of ferroptosis). The results indicated that HSPB1 overexpression decreased erastin-induced growth inhibition and ROS production in breast cancer cells, and Fer-1 treatment showed a synergistic effect with HSPB1 overexpression (Supplementary Fig. S5D, E). Consistently, HSPB1 knockdown led to opposite results, and treatment with Fer-1 attenuated the effect of erastin in HSPB1 knockdown cancer cells (Supplementary Fig. S5D, E). The results observed in MDA-MB-231/DOX cells further confirmed the above findings (Supplementary Fig. S5F–H). Thus, these observations indicated a significant role of HSPB1 in the regulation of ferroptosis in breast cancer cells.

Previous study has reported the association between doxorubicin and ferroptosis [29], however, the detailed anti-tumor effect of doxorubicin-induced ferroptosis is not fully elucidated. Therefore, we further investigated whether HSPB1 could regulate the anti-cancer activity of doxorubicin through modulating ferroptosis in breast cancer cells. Indeed, doxorubicin treatment led to increased levels of ROS and malondialdehyde (MDA, the metabolite of lipid peroxidation), two surrogate markers for ferroptosis, indicating that doxorubicin could induce ferroptosis in breast cancer cells (Fig. 3E, F, Supplementary Fig. S6A, B). Significantly, the effect caused by doxorubicin treatment was attenuated by HSPB1 overexpression and enhanced by HSPB1 knockdown (Fig. 3E, F, Supplementary Fig. S6A, B). Furthermore, Fer-1 was added in the HSPB1-overexpressing or HSPB1-knockdown cells with the presence of doxorubicin. The results showed that Fer-1 treatment could attenuate the effect of doxorubicin-induced ferroptosis (Fig. 3G–I, Supplementary Fig. S6C–E), as indicated by the recovery of cell viability and the reduction of cellular ROS and MDA levels. Moreover, Fer-1 exhibited a synergistic promoting effect with HSPB1 overexpression and had an antagonistic effect with HSPB1 knockdown (Fig. 3G–I, Supplementary Fig. S6C–E). As expected, similar results of HSPB1 knockdown were found in MDA-MB-231/DOX cells, which was reflected in the inhibited cell viability and upregulated ROS and MDA levels (Supplementary Fig. S6F–H). We also investigated the role of HSPB1 in paclitaxel (PTX)-induced ferroptosis, which is another frequently-used chemotherapeutics for breast cancer patients [30]. The results indicated that HSPB1 could promote the resistance to PTX through inhibiting PTX-induced ferroptosis, as determined by the cell viability and ROS production under different conditions (Supplementary Fig. S7). Altogether, these results strengthened the effect of HSPB1 on doxorubicin resistance through regulating ferroptotic cell death in breast cancer cells.

### HSPB1 suppresses ferroptosis via regulating the activation of NF-κB signaling

**Fig. 4:**
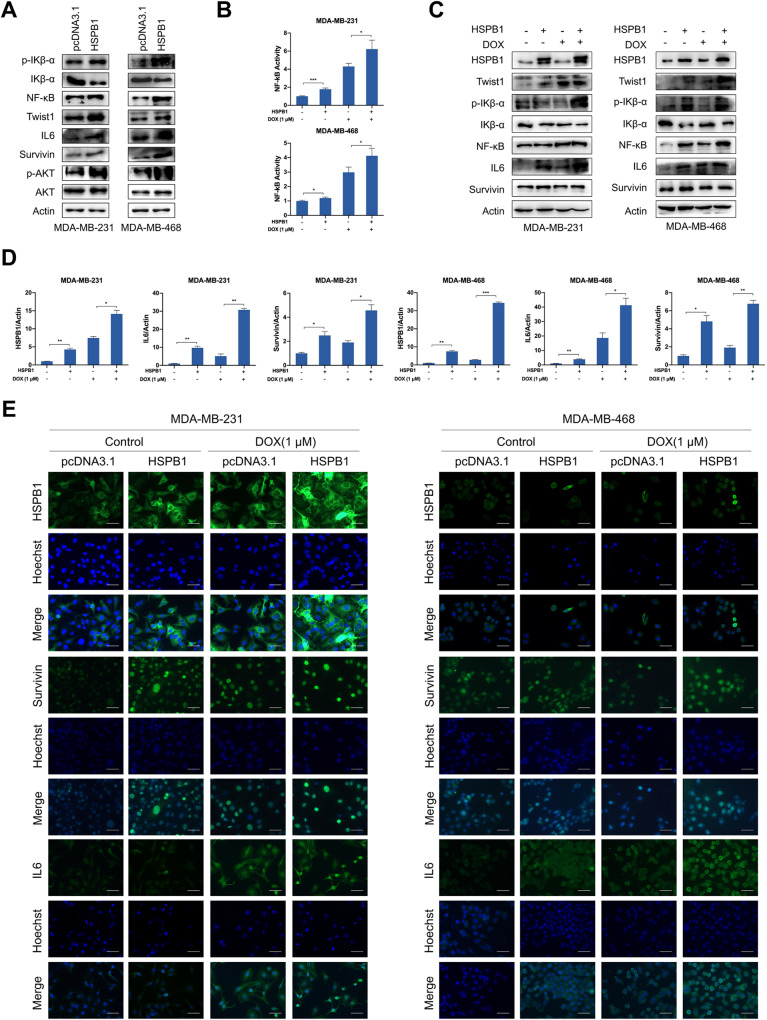
HSPB1 overexpression enhanced activation of NF-κB signaling. **A** Western blot was performed using cell lysates of MDA-MB-231 and MDA-MB-468 cells with or without HSPB1 overexpression. NF-κB transcriptional activity was determined by NF-κB activation reporter assay (**B**), western blot (**C**), and qRT-PCR (**D**). **E** After 24 h of treatment with 1 μM doxorubicin, immunofluorescence was performed to detect the expression of HSPB1, Survivin, and IL6 in MDA-MB-231 and MDA-MB-468 cells with or without HSPB1 overexpression. Scale bar = 50 μm. (*P < 0.05, **P < 0.01, ***P < 0.001).

Reportedly, NF-κB signaling pathway contributed to the progression of a variety of diseases by regulating ferroptosis, such as polycystic ovary syndrome [31], colorectal cancer [32], glioblastoma [33], and pancreatic cancer [34]. Therefore, we speculate whether HSPB1 regulates ferroptosis through NF-κB signaling pathway in breast cancer. HSPB1 overexpression led to decreased expression of IKβ-α (a negative regulator of NF-κB) and increased expression of NF-κB, p-IKβ-α, and downstream molecules of NF-κB (Twist1, IL6, and Survivin) in breast cancer cells (Fig. 4A), indicating the potential role of HSPB1 in regulating NF-κB activity. Using NF-κB reporter assay, we found that doxorubicin treatment led to upregulated NF-κB transactivation activity, and HSPB1 overexpression further enhanced the effect induced by doxorubicin (Fig. 4B). Consistently, the protein levels of p-IKβ-α, NF-κB, and its target genes were increased and the protein expression of IKβ-α was decreased following doxorubicin treatment, and HSPB1 overexpression led to an increased effect of doxorubicin (Fig. 4C). The qRT-PCR and IF assays also indicated similar expression changes of Survivin and IL6 in breast cancer cells (Fig. 4D, E). Moreover, HSPB1 knockdown could diminish the effect of doxorubicin treatment on the activation of NF-κB signaling (Supplementary Fig. S8). These data indicated that HSPB1 was involved in the activation of NF-κB signaling.

### HSPB1 promotes the ubiquitination-mediated degradation of Ikβ-α, leading to increased nuclear translocation of NF-κB in breast cancer cells

**Fig. 5:**
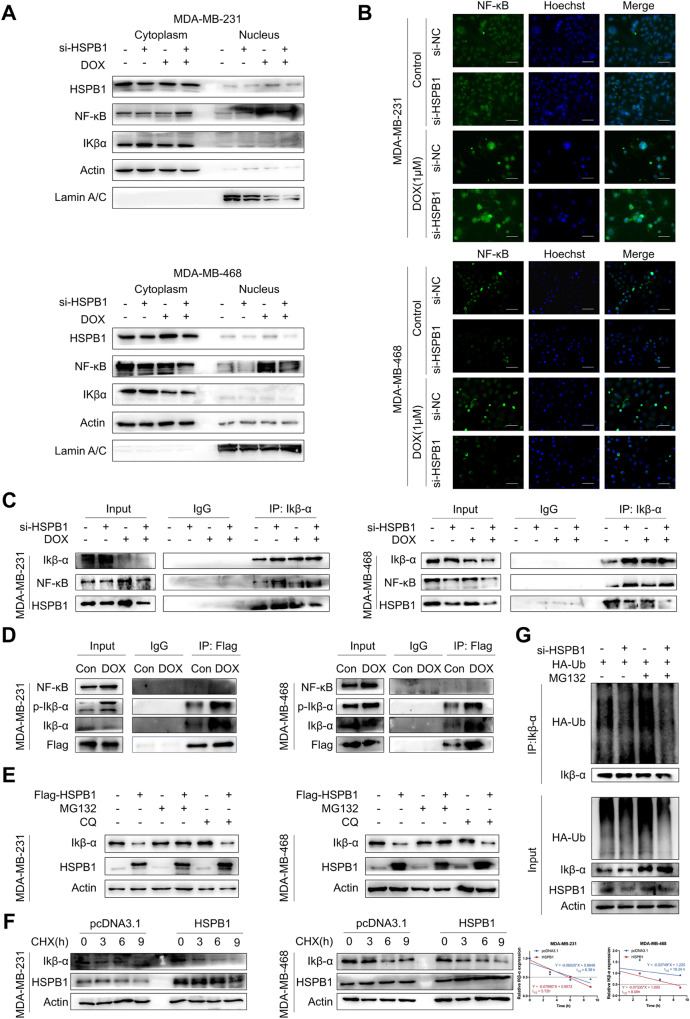
HSPB1 was involved in nuclear translocation of NF-κB through promoting ubiquitination-mediated degradation of Ikβ-α. **A** The protein levels in nuclear and cytoplasmic lysates were detected by western blot. Actin was used as an internal control for cytoplasmic lysates, and Lamin A/C was used as a loading control for nuclear lysates. **B** After 24 h of treatment with 1 μM doxorubicin, MDA-MB-231 and MDA-MB-468 cells with or without HSPB1 knockdown were stained with anti- NF-κB antibody to investigate subcellular localization and expression levels. Scale bar = 50 μm. **C** Coimmunoprecipitation (Co-IP) analysis of Ikβ-α in cells treated with 1 μM doxorubicin for 24 h were evaluated for the presence of Ikβ-α, NF-κB, and HSPB1. **D** The interaction between HSPB1 and Ikβ-α or NF-κB in MDA-MB-231 and MDA-MB-468 cells after treatment with 1 μM doxorubicin was determined by Co-IP assay. **E** MDA-MB-231 and MDA-MB-468 cells were treated with MG132 or CQ, and western blot was used to detect the effect of HSPB1 on the expression of Ikβ-α. **F** MDA-MB-231 and MDA-MB-468 cells were transfected with HSPB1-overexpressing vectors or control vectors. After treatment with 100 μg/ml cycloheximide (CHX) for the indicated time, cells were collected for WB analysis. ImageJ software was used to quantify band intensity. **G** HEK293T cells with or without HSPB1 knockdown were simultaneously transfected with HA-Ub expression plasmids. Then, cells were treated with or without 10 μM MG132 for 6 h and collected for immunoprecipitation with anti- Ikβ-α antibody and ubiquitination detection.

The IKβ-α could bind with NF-κB to keep it in the cytoplasm, preventing nuclear translocation of NF-κB. There is evidence showing that HSPB1 could enhance the ubiquitination-mediated degradation of Ikβ-α through binding with phosphorylated Ikβ-α, leading to the release of NF-κB from the cytoplasmic NF-κB/Ikβ-α complex and enhanced NF-κB activity [35]. We also revealed that doxorubicin treatment led to increased nuclear translocation of NF-κB, which was inhibited by HSPB1 knockdown (Fig. 5A, B). Moreover, the co-IP assay demonstrated that doxorubicin treatment could attenuate the binding between Ikβ-α and NF-κB, while HSPB1 knockdown could restore the inhibited interaction of Ikβ-α with NF-κB (Fig. 5C). In addition, the results also indicated an increase in the binding activity between HSPB1 and Ikβ-α induced by doxorubicin treatment (Fig. 5D). Given our above-mentioned results (Fig. 4A, Supplementary Fig. S8A), which revealed the obvious effect of HSPB1 on the expression of Ikβ-α in breast cancer cells, we further examine the role of HSPB1 in regulating the protein stability of Ikβ-α. 10 μM MG132 (a proteasome inhibitor) or 20 μM chloroquine (CQ, a lysosome inhibitor) was used to treat breast cancer cells to investigate the possibility of its proteasomal or lysosomal degradation. The results showed that overexpression of HSPB1 led to decreased expression of Ikβ-α in control and CQ group (Fig. 5E). MG132 treatment led to upregulated expression of Ikβ-α compared to the other two groups, however, the expression of Ikβ-α was less affected by HSPB1 overexpression in MG132 group (Fig. 5E). Next, breast cancer cells transfected with an empty vector or with HSPB1 overexpressing vector were treated with 100 μg/ml cycloheximide (CHX, a protein synthesis inhibitor). The results showed that overexpression of HSPB1 markedly decreased the half-life of Ikβ-α from 6.39 h (CHX + Vector) to 5.72 h (CHX + HSPB1) in MDA-MB-231 cells and from 19.34 h (CHX + Vector) to 8.08 h (CHX + HSPB1) in MDA-MB-468 cells (Fig. 5F), suggesting that HSPB1 could decrease the protein stability of Ikβ-α. We further examined whether HSPB1 regulated the ubiquitination of Ikβ-α in breast cancer cells. The results indicated that HSPB1 knockdown decreased the ubiquitinated levels of Ikβ-α compared to the control group (Fig. 5G). These findings indicated that HSPB1 regulated the activity of NF-κB through contributing to ubiquitination-mediated degradation of Ikβ-α in breast cancer cells.

### Restoring NF-κB activity attenuates the suppressive effect of HSPB1 knockdown in breast cancer cells

**Fig. 6:**
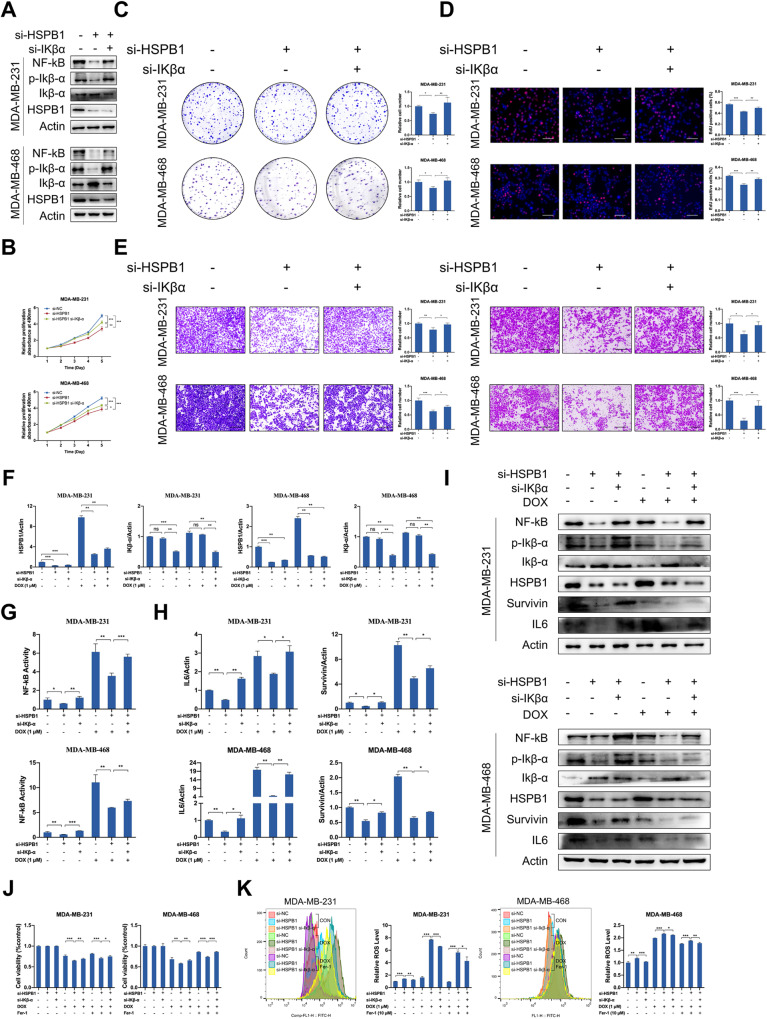
Ikβ-α knockdown partially restored the suppressive effect of HSPB1 knockdown in breast cancer cells. MDA-MB-231 and MDA-MB-468 cells were transfected with negative control siRNA (Ctrl-siRNA), ctrl-siRNA + si-HSPB1, or si-HSPB1 + si-Ikβ-α for 48 h. **A** The expression levels of HSPB1, Ikβ-α, p-Ikβ-α, and NF-κB were determined by western blot. The MTT assay (**B**), colony formation assay (**C**), and EdU assay (**D**) were used to detect cell proliferative ability. Scale bar = 100 μm. **E** Transwell assay was performed to determined cell migration and invasion. Scale bar = 100 μm. **F** The transfected MDA-MB-231 and MDA-MB-468 cells were treated with or without 1 μM doxorubicin. The HSPB1 and Ikβ-α expression was detected by qRT-PCR in indicated cells. **G** NF-κB transactivation was detected by NF-κB activation reporter assay. **H** The RNA expression levels of Survivin and IL6 were detected by qRT-PCR. **I** The protein expression levels of HSPB1, Ikβ-α, p-Ikβ-α, NF-κB, Survivin and IL6 were determined by western blot. **J** The MTT assay was used to analyze the viability of transfected cells following doxorubicin plus either DMSO or Fer-1 treatment. **K** Cellular ROS levels were detected in transfected cells after doxorubicin plus either DMSO or Fer-1 treatment. (*P < 0.05, **P < 0.01, ***P < 0.001).

We further examined whether additional activation of NF-κB could diminish the inhibitory effect caused by HSPB1 knockdown. The increased expression of Ikβ-α and decreased expression of NF-κB caused by HSPB1 knockdown could be restored by knockdown of Ikβ-α (Fig. 6A). Moreover, Ikβ-α knockdown could attenuate the inhibited effect of HSPB1 knockdown on cell proliferation, migration, and invasion (Fig. 6B–E, Supplementary Fig. S9A). The NF-κB activity could be restored in HSPB1 knockdown cells by knockdown of Ikβ-α with or without doxorubicin treatment (Fig. 6F–I), as determined by NF-κB reporter assay and detection of the expression of NF-κB target genes. In addition, the inhibited cell viability and enhanced ROS production induced by doxorubicin or erastin in HSPB1 knockdown cells could be restored by simultaneous knockdown of Ikβ-α (Fig. 6J–K, Supplementary Fig. S9B). These results suggest that HSPB1 regulated the biological behaviors and doxorubicin-induced ferroptosis through NF-κB activity in breast cancer cells.

### HSPB1 enhances malignant behaviors of breast cancer cells by regulating IL6 expression

**Fig. 7:**
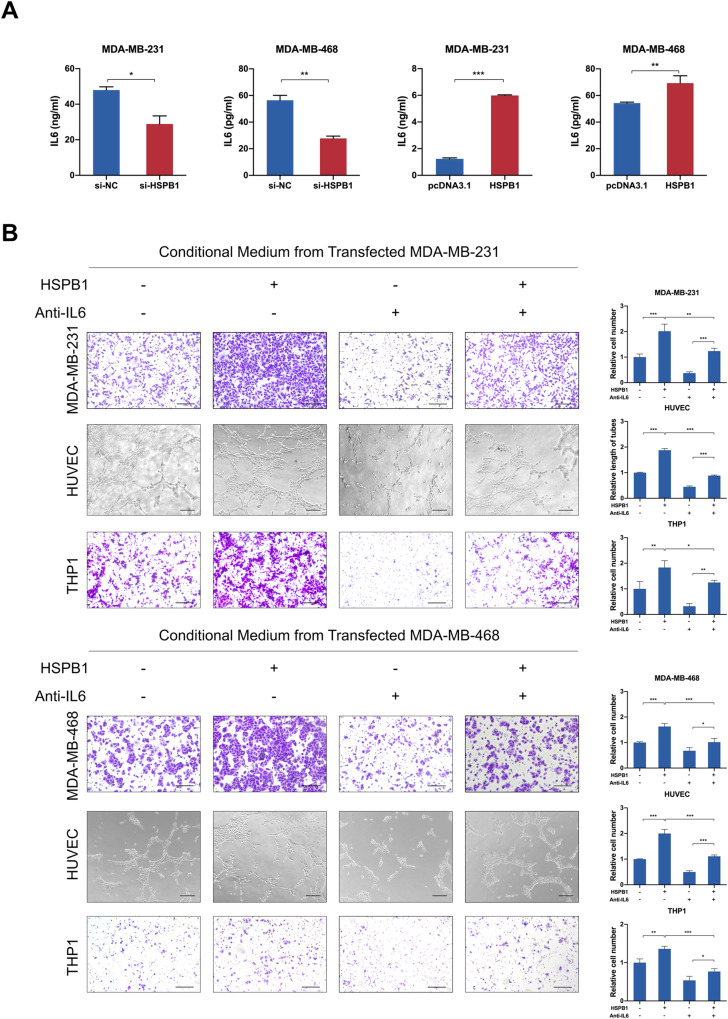
HSPB1 promoted breast cancer progression through regulating IL6. **A** The ELISA assays were conducted to detect the expression levels of IL6 in transfected cells. **B** IL6 neutralizing antibody treatment (2.5 μg/ml) efficiently reversed the promoting effects of conditioned medium from HSPB1-overexpressing MDA-MB-231 or MDA-MB-468 cells on the breast cancer cell migration (Scale bar = 100 μm), tube formation (Scale bar = 200 μm), and THP1 migration (Scale bar = 100 μm). (*P < 0.05, **P < 0.01, ***P < 0.001).

IL6 belongs to the family of IL (interleukin)-6-type cytokines, which could be secreted by myriads of cells [36, 37], including monocytes, fibroblasts, endothelial cells, keratinocytes, and cancer cells. Previous studies reported that IL6 showed a significant role in regulating various cellular functions [38, 39], such as cell proliferation, metastasis, vascular permeability, metabolism, and infiltration of immune cells. Given the obvious effect of HSPB1 on the expression of IL6 in breast cancer cells detected by our above results, we further investigate whether the secretion of IL6 could be influenced by HSPB1. The ELISA assay indicated that HSPB1 knockdown led to decreased IL6 secretion while HSPB1 overexpression promoted the secretion of IL6 (Fig. 7A). Next, we evaluated the influence of secreted IL6 on the malignant behaviors of breast cancer. The supernatants from HSPB1 overexpressing cells led to increased migration ability of breast cancer cells and tube formation of HUVECs, while the supplement of IL6 neutralizing antibodies could partly attenuate the promoted effect (Fig. 7B).

As the major component of tumor microenvironment, tumor-associated macrophages play an important role in the whole process from tumor occurrence to metastasis [40]. We used CIBERSORT, xCell, and quanTIseq algorithms to evaluate the levels of immune cell infiltration in tumor microenvironment. The results of Spearman correlation analysis showed that the expression level of HSPB1 was positively correlated with the infiltration level of M2 macrophages and the ratio of M2 to M1, while negatively correlated with the infiltration level of M1 macrophages (Supplementary Fig. S10A). It could be speculated that HSPB1 might play an important role in the progression of breast cancer by promoting the infiltration of M2 macrophages. Previous studies reported that tumor cells could produce numerous chemokines to attract macrophages, which further dictate the fate of tumor development and progression through secreting an assorted array of cytokines. It is well-established that IL6 is an essential chemokine for the recruitment of macrophages in cancers [39, 41], such as cervical cancer and prostate cancer. Accordingly, we further evaluated whether HSPB1 could modulate the infiltration of macrophages in breast cancer. The conditioned medium from HSPB1 knockdown cells led to decreased effect on the migration ability of macrophages and attenuated chemotaxis to macrophages, while the conditioned medium from HSPB1 overexpressing cells exhibited the opposite effect (Supplementary Fig. S10B–E). To confirm that the enhanced behaviors of macrophages induced by HSPB1 were mediated by IL6, anti-IL6-neutralizing antibodies were used. The results showed that the addition of IL6 neutralizing antibodies could abrogate the enhanced effect of supernatants from HSPB1 overexpressing cells on THP1 migration (Fig. 7B), suggesting the involvement of HSPB1-IL6 pathway in the regulation of accumulation of macrophages in breast cancer. Collectively, these observations indicated that HSPB1 could aggravate the progression of breast cancer through promoting IL6 secretion.

### HSPB1 promotes tumor growth, doxorubicin resistance, and metastasis of breast cancer in vivo

**Fig. 8:**
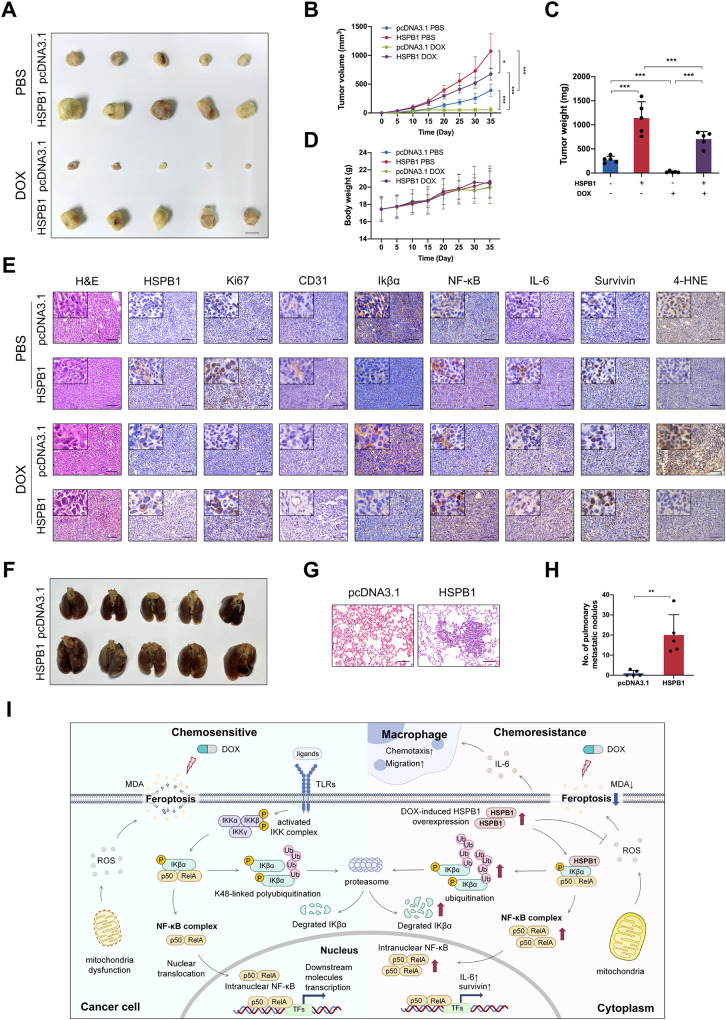
HSPB1 promoted chemoresistance and metastasis of breast cancer. **A** Control or HSPB1-overexpressing MDA-MB-231 cells were transplanted into the flanks of nude mice, followed by doxorubicin or PBS treatment (*n* = 5). Images of tumors harvested. Scar bar = 1 cm. **B** The tumor volumes were measured every five days. **C** Tumor weights were recorded after sacrifice of the mice. **D** The body weights of mice were measured every five days. **E** H&E staining showed the tissue morphology of transplanted tumors. Representative pictures of immunohistochemical staining of HSPB1, Ki67, CD31, Ikβα, NF-κB, IL-6, Survivin, and 4-HNE in the tumor tissues. Scale bar = 100 μm. **F** Pictures of lung metastatic nodules. **G** H&E staining showed the tissue morphology of lungs isolated from indicated mice. Scale bar = 100 μm. **H** The statistical graph of lung metastatic nodules. **I** The mechanistic schematic model of the role of HSPB1 in breast cancer. (*P < 0.05, **P < 0.01, ***P < 0.001).

We further evaluated the function of HSPB1 in breast cancer in vivo using a nude mouse xenograft model. The stable HSPB1-overexpressing or control MDA-MB-231 cells were injected into the flanks of BALB/c nude mice. When the tumor volume reached 50 mm^3^, PBS or doxorubicin was injected intravenously. As shown in Fig. 8A–C, HSPB1 overexpression led to increased tumor volume and tumor weight. Moreover, the tumors in the doxorubicin-injected mice were smaller and lighter than those in PBS-treated mice (Fig. 8A–C), indicating the suppressive effect of doxorubicin on the growth of breast cancer in vivo. Significantly, the tumors in the doxorubicin-injected mice bearing HSPB1-overexpressing cells were significantly larger and heavier compared to those in doxorubicin-injected mice implanted with control cells (Fig. 8A–C), suggesting that overexpression of HSPB1 attenuated the anti-tumor effect of doxorubicin. No significant body weight loss occurred during the treatment (Fig. 8D), excluding the drug-related toxicities. HE staining was used to evaluate the morphology of the tumors (Fig. 8E). The IHC analysis indicated that the expression changes of IKβ-α, NF-κB, and its target genes in the tumor tissues were consistent with the findings in vitro (Fig. 8E). Also, IHC staining of 4-HNE confirmed that DOX enhanced lipid peroxidation in tumor tissue, while HSPB1 could significantly suppress it (Fig. 8E). Moreover, doxorubicin decreased the protein expression of Ki-67, which was enhanced by HSPB1 overexpression (Fig. 8E). These results demonstrated that HSPB1 increased the resistance of breast cancer to doxorubicin-induced ferroptosis via NF-κB signaling in vivo.

In addition, we also investigated the effect of HSPB1 on breast cancer metastasis in vivo using a pulmonary metastasis model. The stable HSPB1-overexpressing or control MDA-MB-231 cells were injected into BALB/c nude mice via the tail vein. The lung metastatic burden was significantly higher in HSPB1-overexpressing mice, in which the number of metastatic mice and lung metastatic foci was increased compared to those in control group (Fig. 8F–H). Hematoxylin and eosin (H&E) staining was used to pathologically confirm the metastatic nodule in the dissected lungs, and overexpression of HSPB1 significantly increased the size and number of lung metastatic nodules (Fig. 8F–H). Collectively, these results suggested that HSPB1 facilitated tumor growth, doxorubicin resistance, and metastasis in vivo (Fig. 8I).

## Discussion

Due to the lack of ER, PR, and HER2 receptors, there is no ideal target available for effective therapy against TNBC [42]. Chemotherapy remains the first-line treatment for most TNBC patients [3, 43]. However, TNBC is an aggressive phenotype and often refractory to chemotherapy, leading to disease progression, relapse and metastasis, which is the reason for more than 90% death of TNBC patients [44, 45]. Therefore, finding novel targets that could clarify the underlying mechanisms and be exploited to overcome chemoresistance are critical for improving the prognosis of breast cancer patients. In the present study, we substantiated that HSPB1 could prevent breast cancer cells from chemotherapy-induced ferroptosis, and exhibited a significant role in mediating the progression and chemoresistance of breast cancer.

Ferroptosis is a nonapoptotic form of programmed cell death characterized by iron-dependent peroxidation accumulation of phospholipids, which is regulated by cell metabolism, redox homeostasis, and various cancer-related signaling pathways [46]. It has been reported that ferroptosis plays a pivotal role in suppressing breast cancer growth by modulating various tumor properties. Therefore, induction of ferroptosis via targeting ferroptosis-related genes has become a therapeutic strategy to combat tumor progression and chemoresistance. For instance, inhibition of cannabinoid receptor type 1 promoted the effect of erastin/RSL3 in inducing ferroptosis and enhanced the inhibitory effect on TNBC growth [47]. FGFR suppression triggered ferroptosis and enhanced susceptibility to anti-HER2 therapy in recalcitrant HER2-positive breast cancer [48]. Cystathione β-synthetase silence made TNBC cells more vulnerable to oxidative stress and prone to ferroptosis through reprograming transsulfuration, and finally impaired tumor progression [49]. At the same time, emerging evidence showed that ferroptosis-related genes could be indicators of patient prognosis and therapeutic efficacy. For example, the expression status of ACSL4 and GPX4, the positive and negative regulator of ferroptosis, respectively, could serve as independent predictive factors for pCR of paclitaxel-cisplatin-based neoadjuvant chemotherapy, and prognostic factors for patient survival [50]. Besides, a cell death index (CDI) that includes a ferroptosis-related gene TCF7L2 could accurately predict the clinical prognosis and drug sensitivity of TNBC after surgery [51]. However, numerous molecules may play important roles in the whole process of ferroptosis. Therefore, more studies are needed to reveal the role and mechanism of ferroptosis in the progression and chemoresistance of breast cancer.

HSPB1 is a member of the small heat shock proteins, which could be highly inducible under stressful conditions [52]. Previous studies have shown that HSPB1 exerted a tumor-promoting effect in various cancers through its proliferative and anti-apoptotic functions, such as esophageal squamous cell [52], prostate cancer [53], carcinoma squamous cell carcinoma of tongue [54]. However, the effect of HSPB1 on the progression and chemoresistance and the underlying mechanism in breast cancer has not yet been fully explored. In the study, we identified upregulated HSPB1 expression in breast cancer tissues based on the results obtained from several public datasets, which was further confirmed using tissues from our cohort. Moreover, high expression of HSPB1 was associated with a worse prognosis in breast cancer patients. Subsequently, our results revealed that HSPB1 could promote breast cancer proliferation, migration, invasion, and doxorubicin resistance.

Doxorubicin (DOX) is a cytotoxic anthracycline antibiotic, widely used for treating a variety of cancers, which is considered the foundation of chemotherapy for breast cancer [55]. Accumulating studies have shown that doxorubicin promoted cancer cell death through inhibiting DNA replication and generating H_2_O_2_, which is the Fenton reaction substrate leading to increased ROS production [33, 56]. Thus, doxorubicin might kill cancer cells by indirectly inducing ferroptosis. Previous studies reported that HSPB1 might be a negative regulator of ferroptosis [28], which was upregulated following erastin treatment in several cancer cells, such as Hela cells, U2OS and LNCap. Here, we observed similar functions of doxorubicin in breast cancer cells. Doxorubicin treatment substantially increased the intracellular ROS and MDA levels in breast cancer cells, which are the critical executors of ferroptosis [14]. Consistent with the previous study, we identified the induced expression of HSPB1 after erastin treatment [28]. In addition, our results also revealed the doxorubicin-mediated upregulation of HSPB1 in breast cancer, indicating that HSPB1 might be involved in the regulation of doxorubicin-induced ferroptosis. In fact, HSPB1 overexpression led to attenuated doxorubicin-induced ferroptosis, as determined by the reduction of cellular concentrations of lipid ROS and MDA, and treatment with Fer-1 could further enhance the effect caused by HSPB1 overexpression. These results suggested that HSPB1 overexpression inhibited ferroptosis and ultimately led to a decrease of the doxorubicin sensitivity of breast cancer cells. We further constructed the doxorubicin-resistant cells (MDA-MB-231/DOX), and significantly upregulated expression of HSPB1 was identified in the chemoresistant cells compared to the corresponding parental cells. HSPB1 knockdown could render chemoresistant cells to regain sensitivity to doxorubicin treatment through promoting doxorubicin-induced ferroptosis. Similar findings have been observed following paclitaxel treatment, another wildly-used anti-cancer drug, indicating the significant role of HSPB1 in chemotherapy-induced ferroptosis.

NF-κB pathway is essential for governing inflammatory response in cancers and is implicated as a hallmark of cancer progression and a potential therapeutic target [57]. There are many molecules involved in tumor progression that regulate ferroptosis through NF-κB pathway. For instance, SIRT6 promoted ferroptosis and suppressed glycolysis through inactivating the NF-κB pathway [34]. LIFR inactivated NF-κB signaling through SHP1, causing downregulation of the iron-sequestering cytokine LCN2, which further rendered sensitivity to ferroptosis inducers [58]. To gain insight into the intricate molecular mechanism by which HSPB1 regulated doxorubicin-induced ferroptosis, we first explored whether HSPB1 was able to regulate NF-κB pathway to exhibit a tumor-promoting role in breast cancer. Here, we found that HSPB1 could promote the nuclear translocation and activity of NF-κB in breast cancer cells, as determined by the elevated expression of its target genes. A previous study reported that ubiquitin‐mediated degradation of NF‐κB inhibitor proteins, known as IκBs, was the major cause of activation of NF-κB signaling [59]. Among the IκB family, IκB-α is the most well‐studied member. HSPB1 was reported to promote the 26 S proteasome-mediated degradation of ubiquitinated proteins, such as phosphorylated IKβ-α [35]. Consistently, our results also demonstrated that HSPB1 could bind with IKβ-α and promote ubiquitination-mediated IKβ-α degradation, leading to enhanced nuclear translocation and activity of NF-κB in breast cancer cells. Moreover, doxorubicin treatment could further reinforce the mutual combination between HSPB1 and IKβ-α, indicating the underlying mechanism involved in HSPB1-mediated doxorubicin resistance. In addition, the inhibitory effect caused by HSPB1 knockdown could be reversed by the knockdown of IKβ-α in breast cancer cells, which further demonstrated that HSPB1 regulated the aggressive behaviors of breast cancer cells through NF-κB activity.

NF-κB is a well-known transcriptional factor involved in the expression of several cytokines. In this study, high expression of IL6 was identified in HSPB1-overexpressing group, and the inhibited expression of IL6 caused by HSPB1 knockdown could be rescued by IKβ-α knockdown. IL6 could be secreted from various cells in cancer tissues, including myeloid cells, cancer-associated fibroblasts, and cancer cells [60–62]. Our results revealed that HSPB1 overexpression promoted the secretion of IL6, whereas HSPB1 knockdown led to decreased IL6 secretion in breast cancer cells. A previous study reported that high serum IL6 concentration was associated with poor prognosis of breast cancer patients [63]. Moreover, IL6 family members were reported to promote cell migration, angiogenesis, as well as macrophage recruitment and infiltration [64, 65]. Therefore, we further investigated the effect of HSPB1-induced IL6 in the progression and immune infiltration of breast cancer. The conditioned medium collected from HSPB1-overexpressing cells significantly promoted the migration of cancer cells and macrophages as well as the angiogenesis of HUVECs, which could be abrogated by the supplement of IL6 neutralizing antibodies. IL6 has also been reported to induce macrophage polarization to the M2-phenotype in various cancers [66, 67], however, the association between HSPB1 and M2 polarization in breast cancer needed to be further elucidated. Overall, IL6 showed a significant role in the HSPB1-mediated malignant behaviors, and it is possible that HSPB1 is involved in the regulatory function of IL6 through modulating NF-κB activity in breast cancer.

In conclusion, our results uncovered that HSPB1 is a significant drug-resistant factor, which could inhibit chemotherapy-induced ferroptosis in breast cancer through promoting the activation of NF-κB signaling pathway. Our observations provide a foundational rationale that targeting HSPB1 and combinational induction of ferroptosis with anticancer drugs would be a potential therapeutic strategy to overcome chemoresistance in breast cancer.

## Materails and methods

### Patients and samples

The breast cancer tissues and adjacent normal tissues from the same patients were obtained from patients diagnosed with breast cancer undergoing surgery at the Qilu Hospital of Shandong University from March 2007 to January 2017. Survival time was defined as the time from the date of operation until the date of the last follow-up or death. This study was approved by the Ethical Committee of Qilu Hospital of Shandong University. The informed consents for participation in this study from all patients were obtained.

### Cell culture

Human breast cancer cell lines (MDA-MB-231 and MDA-MB-468), human umbilical vein endothelial cells (HUVECs), THP-1 cells, and HEK293T cells were purchased from American Type Culture Collection (ATCC, Manassas, Virginia, USA). The cells were authenticated by STR analysis (Suzhou, China) and tested for negative mycoplasma contamination using Mycoplasma Detection Kit (Sigma-Aldrich, MP0050, Missouri, USA). MDA-MB-231, MDA-MB-468, HUVEC, and HEK293T cell lines were cultured in DMEM (CM10017, Macgene, Beijing, China), and THP1 cells were maintained with RPMI-1640 medium. The culture medium was supplemented with 10% fetal bovine serum (FBS, Gibco, Carlsbad, California, USA), 100 U/ml penicillin, and 100 µg/ml streptomycin. These cells were incubated in a humidified incubator containing 5% CO_2_ at 37 °C.

### Reactive oxygen species (ROS) analysis

The differently treated cells were digested, washed twice with PBS, and incubated with 10 µM 2ʹ,7ʹ-dichlorofluorescein diacetate (H2DCFDA, ID3130, Solarbio, Beijing, China) in serum-free and antibiotic-free DMEM medium at 37 °C for 30 min in the dark. After washing with PBS, the intracellular ROS levels were then analyzed using a FACSCalibur flow cytometer (BD Biosciences, New Jersey, USA).

### Malondialdehyde (MDA) Assay

The malondialdehyde (MDA) assay kit (Beyotime, S0131S, Shanghai, China) was applied to detect the level of lipid peroxidation according to the manufacturer’s protocol. In brief, 2×10^6^ treated or transfected cells were collected and mixed with 150 µl lysis buffer on ice for 30 min. Then 100 µl of sample supernatant was mixed with 200 µl of TBA working solution for 15 min at 100 °C, while the residual supernatant of each sample was used for protein concentration detection. After centrifugation, the supernatant was collected, and MDA content was displayed as the absorbance value measured at 532 nm. The MDA concentration was calculated, and the relative content of MDA was finally obtained as the ratio of their MDA concentration to their protein concentration.

### NF-κB reporter assay

The luciferase-based NF-κB reporter vector was obtained from Yeasen Biotech Co., Ltd (11501ES03, Shanghai, China). The NF-κB reporter vector and reference Renilla luciferase vector (Promega, E2241, Madison, Wisconsin, USA) were co-transfected into breast cancer cells at a ratio of 10:1. At 48 h post-transfection, the Dual-Luciferase Reporter Assay System (Promega, E2920, Madison, Wisconsin, USA) was used to measure the luciferase activities with EnSpire^TM^ Multimode Plate Reader (PerkinElmer, Waltham, Massachusetts, USA). The Firefly luciferase activities were normalized with the corresponding Renilla luciferase activities.

### Enzyme-Linked Immunosorbent Assay (ELISA) analysis

The levels of IL-6 secreted by the breast cancer cells in their medium were tested using a CUSABIO® Human Interleukin 6, IL-6 ELISA kit (Cusabio Biotech Co. Ltd., CSB-E04638h, Wuhan, China) based on the manufacturer’s instruction. Briefly, the culture medium of transfected breast cancer cells was replaced by equivalent DMEM without serum 24 h before collection. Then the medium was harvested and the supernatant was obtained by 1000 × g centrifugation for 15 min at 4 °C. 100 µl medium supernatant was used for incubation in the ELISA plate, followed by interaction with biotin-labeled IL-6 antibody working solution and HRP-avidin working solution successively. Then, 90 µl transmembrane domain substrate was added into the plate for 15-30 min in the dark and 50 µl stop solution was subsequently added. Finally, the absorbance was measured at 450 nm by a Microplate Reader (PerkinElmer, Waltham, Massachusetts, USA). The specific concentration value of IL-6 (pg/mL) was calculated based on the standard curve.

### In vivo animal study

Female BALB/c nude mice (4-6 weeks) were purchased from the GemPharmatech Co., Ltd. (Nanjing, China). For subcutaneous inoculation, 1×10^7^ MDA-MB-231 cells stably expressing HSPB1 or control vectors were resuspended in 200 μl PBS and implanted subcutaneously into the right flank regions of the mice. When the subcutaneous tumors reached an average size of 50 mm^3^, the nude mice from each group were randomly and equally divided into two subgroups, which were treated with 2 mg/kg of DOX or an equal volume of vehicle control via intravenous injection every 3 days for a total of 7 times. Tumor size was measured with calipers every 5 days, and tumor volume was calculated using the formula: volume = length × (width)^2^/2. The mice were sacrificed at the end of the experiments, and the subcutaneous tumors were weighed and photographed. For in vivo metastasis assay, 5 × 10^5^ MDA-MB-231 cells stably expressing HSPB1 or control vectors were resuspended in 200 μl PBS and intravenously injected into the tail veins of nude mice. 4 weeks later, all the mice were sacrificed and the lungs were harvested and analyzed. Hematoxylin and eosin (H&E) staining was used to confirm the tissue morphology. All animal investigations were approved by the Shandong University Animal Care and Use Committee.

### Immunohistochemistry (IHC)

The tissues were first fixed in formalin, dehydrated, embedded in paraffin, and then sliced into 4 μm sections. The sections were deparaffinized in xylene and hydrated with gradient alcohol. Citrate buffer was further used for antigen retrieval, and 3% H_2_O_2_ was used to eliminate endogenous peroxidase activity. Sections were then blocked by BSA. Subsequently, the sections were incubated with primary antibody at 4 °C overnight. Next day, after washed by PBS, the sections were incubated with peroxidase-conjugated secondary antibody at room temperature for 15 min and stained with diaminobenzidine working solution. Then, the sections were counterstained with hematoxylin, dehydrated with gradient alcohol, mounted, and photographed under the Olympus light microscope. HSPB1 expression was analyzed and scored based on the intensity and the percentage of positively stained tumor cells in the tissue samples, which was evaluated using IHC scores: IHC score = percentage score × intensity score. Percentage staining scores were defined as hereunder mentioned: (i) 0, <10%; (ii) 1, 10–25%; (iii) 2, 25–50%; (iv) 3, 50–75%; and (v) 4, >75%; intensity staining scores were divided into four grades as follows: (i) 0, no staining; (ii) 1, light brown; (iii) 2, brown; and (iv) 3, dark brown. Based on the above criteria, HSPB1 expression was classified into four grades: (i) negative, IHC score ≤ 3; (ii) weak, IHC score > 3 and ≤6; (iii) moderate, IHC > 6 and ≤9; and (iii) strong, IHC > 9. According to the IHC scores and prognosis (OS and DFS) of breast cancer patients, we drew receiver operating characteristic (ROC) curves for OS and DFS. Cutoff values were then calculated based on the sensitivity and specificity of ROC curves, and were all calculated as 7. Finally, the breast cancer patients were divided into two groups with HSPB1-high (IHC score >7) and -low (IHC score <7) expression by the cutoff values of IHC scores calculated using the ROC curves.

### Statistical analysis

Analyses were performed using GraphPad Prism 8.0 Software (La Jolla, California, USA). The data are presented as mean ± standard deviation (SD) from three independent experiments. *P*-values were calculated with Student’s *t* test or ANOVA test for comparison of two groups or more than two groups. *P* < 0.05 was considered statistically significant. Survival curves were plotted with the Kaplan–Meier method and compared by the log-rank test. *P* < 0.05 was considered statistically significant.

## Supplementary information

Supplementary Materials

Original western blot

Reproducibility Checklist

## Data Availability

The data and material are available by contacting the corresponding author upon reasonable request.
