# Supplementary material for: HSPB1 facilitates chemoresistance through inhibiting ferroptotic cancer cell death and regulating NF-κB signaling pathway in breast cancer
Source: Cell Death Dis. 2023 Jul 15;14(7):434. doi: 10.1038/s41419-023-05972-0 (PMC10349816; doi:10.1038/s41419-023-05972-0)
Supplement: Supplementary file 1 — Supplementary Materials [file 41419_2023_5972_MOESM1_ESM.pdf]

HSPB1 facilitates chemoresistance through inhibiting ferroptotic cancer cell death and regulating NF-κB signaling pathway in breast cancer

Yiran Liang[1,#], Yajie Wang[1,#], Yan Zhang[1,2], Fangzhou Ye[1], Dan Luo[1], Yaming Li[1], Yuhan Jin[1], Dianwen Han[1], Zekun Wang[1], Bing Chen[3], Wenjing Zhao[3], Lijuan Wang[3], Xi Chen[1], Tingting Ma[1], Xiaoli Kong[1], and Qifeng Yang[1,3,4,*]

[1]Department of Breast Surgery, Qilu Hospital, Cheeloo College of Medicine, Shandong University, Jinan, Shandong, 250012, China

[2]Department of Breast Surgery, Jinan Central Hospital, Cheeloo College of Medicine, Shandong University, Jinan, Shandong, 250013, China

[3]Pathology Tissue Bank, Qilu Hospital of Shandong University, Jinan, Shandong, 250012, China

[4]Research Institute of Breast Cancer, Shandong University, Jinan, Shandong, 250012, China

[#] These authors contributed equally to this work.

[*]Correspondence: Qifeng Yang

Qilu Hospital, Cheeloo College of Medicine, Shandong University, Wenhua Xi Road No. 107, Jinan 250012, Shandong, China; Fax: +86-531-82169470; E-mail: qifengy_sdu@163.com

Supplementary Materials and Methods

Cell transfection

The full length of HSPB1 was cloned into pcDNA3.1 (Invitrogen, Carlsbad, California, USA) to generate pcDNA3.1-HSPB1 constructs. After transfection with the overexpression vectors or empty vectors, cells were selected by G418 (1 μg/ml) for 2 months until the formation of visible clones. The survived cell clones were amplified and the overexpression efficiency of individual clones was confirmed using qRT-PCR assays. The satisfactory clones were identified as stably transfected cells and used for in vivo experiments. The siRNAs were purchased from GenePharma (Shanghai, China), and the target sequences for HSPB1, Ikβ-α, and Control are listed in Table S4. Lipofectamine 2000 (Invitrogen, 11668019, Carlsbad, California, USA) was used for cell transfection following the manufacturer's instructions.

Cell proliferation assays

Cell proliferation assays were performed using MTT assay. Briefly, 1 500 transfected cells were seeded into a 96-well plate. Cell proliferation was assessed after indicated time. The cells were incubated for 4-6 h after the addition of 20 μl MTT (5 mg/ml) per well. Then, the culture supernatant was removed and 100 μl DMSO was added into each well. The absorbance was measured at 490 nm using a Microplate Reader (Bio-Rad, Hercules, California, USA).

IC50 detection and cytotoxic assay

The transfected cells were cultured in 96-well plates (3 000 cells/well). For IC50 detection, cells were grown in complete medium containing different concentrations of doxorubicin (MCE, HY-15142A, New Jersey, USA) or paclitaxel (MCE, HY-B0015, New Jersey, USA) for 48 h. The cell survival rate was further evaluated by MTT assay. For cytotoxic assay, cells were treated differently as indicated using doxorubicin, paclitaxel, or erastin (MCE, HY-15763, New Jersey, USA) with or without ferrostatin-1 (MCE, HY-100579, New Jersey, USA) for 48 h. The cell viability was assessed by MTT assay and presented as relative to the absorbance value of the control group.

Colony formation assay

1 000 Transfected cells were seeded into 6 cm plates and cultured for over 2 weeks. Then, cells were washed with PBS, fixed with methanol for 15 min and subsequently stained with 0.2% crystal violet for 20 min at room temperature. Colonies were counted and photographed.

EdU incorporation assay

The measurement of proliferative cells and nucleic acid were performed using EdU incorporation assay kit (RiboBio, C10310-1, Guangzhou, China) according to the manufacturer's instructions. Briefly, 2 × 104 transfected cells were seeded into a 96-well plate per well for 24 h. After incubation with 50 μM EdU for another 2.5 h, cells were washed with PBS, fixed with 4% paraformaldehyde (PFA) for 30 min, and

permeabilized with 0.5% Triton X-100 for 10 min. Subsequently, cells were stained with 1× Apollo Dye Solution for 30 min and 1× Hoechst for 30 min at room temperature in the dark. The images were obtained under a fluorescence microscope (ZEISS, Jena, Germany) and positive cells were counted.

Wound healing assay

The transfected cells were seeded in a 24-well culture plate and cultured until reaching to 90% confluence. The cell monolayer was scratched using a sterile tip (10 μl), washed with PBS, and cultured with serum-free DMEM. Photographs were taken with a light microscope at the indicated time.

Migration and invasion assays

For migration assays, 80 000 transfected cells resuspend with serum-free DMEM were seeded into the upper insert of transwell chamber (pore size 8 μm; Corning, New York, USA). 700 μl medium supplemented with 20% FBS were put into the lower chambers. For invasion assays, the transwell chambers were coated with Matrigel (Corning, 356232, New York, USA) solution. After incubation for 24-48h, the cells penetrated to the lower surface of the membrane were fixed with methanol for 15 min and then stained with 0.2% crystal violet for 20 min. Then the stained cells were photographed and counted.

RNA preparation and qRT-PCR

Total RNA was extracted from cells or frozen tissues using the RNAiso Plus (Total RNA extraction reagent) (Takara, 9108, Kyoto, Japan). The complementary DNA (cDNA) was synthesized using a PrimeScript reverse transcriptase (RT) reagent kit (Takara, RR037B, Kyoto, Japan). The expression of mRNA was examined by real-time quantitative-PCR (qRT-PCR) using TB Green™ Advantage ® qPCR Premix (Takara, 639676, Kyoto, Japan) and a LightCycler480 Detection System (Roche, Germany). The relative expression of indicated genes was analyzed by the 2−ΔΔCt method. β-actin was used as normalization control. The primer sequences used for qRT-PCR are listed in Table S5.

Western blot analysis

Total protein was extracted from cells or frozen tissues using the RIPA lysis buffer (Beyotime, P0013, Shanghai, China) with protease inhibitor (PMSF) and phosphatase inhibitor (NaF). The protein concentration was determined using a BCA protein assay kit (Beyotime, P0010, Shanghai, China). The protein samples were separated by 10% SDS-PAGE gel and then transferred to a 0.22 μm polyvinylidene difluoride (PVDF) membrane (Millipore, Billerica, Massachusetts, USA) in a wet electron transfer device. Then, the membrane was blocked using 5% skimmed milk in TBST for 1 h at room temperature and incubated with the primary antibody overnight at 4 °C. The next day, after incubation with the secondary antibody for 1 h at room temperature, an enhanced chemiluminescence (ECL) kit (Vazyme, E423-01, Nanjing, China) was used to visualize the target protein. The antibodies are listed in Table S6.

Immunofluorescence staining

5×10^5 cells were seeded into 24-well culture plates for 24 h, and treated with the indicated drugs or left untreated for another 24 h. The cells were washed with PBS, fixed with 4% paraformaldehyde (PFA) for 30 min, and permeabilized with 0.5% Triton X-100 for 10 min. Subsequently, cells were blocked with 10% goat serum in PBS at room temperature for 1 h, and incubated with primary antibody at 4 °C overnight. After incubation with secondary antibody for 2 h at room temperature in the dark, the nuclei were stained by DAPI (Beyotime, C1005, Shanghai, China) at room temperature for 15 min. The stained cells were observed and photographed using a fluorescent microscope (ZEISS, Jena, Germany). The antibodies are listed in Table S6.

Supplementary Figures and Legends

Figure S1. The expression of HSPB1 was upregulated in breast cancer tissues and associated with poor prognosis of breast cancer patients. (A) The heatmaps showing the differentially expressed genes in breast cancer tissues and normal tissues in TCGA and GEO databases. (B) The RNA expression of HSPB1 was upregulated in various cancers according to TCGA database. (C) The protein expression of HSPB1 was increased in breast cancer tissues compared to normal tissues based on the Human Protein Atlas database. (D) High expression of HSPB1 was associated with poor OS of breast cancer patients. (E) High expression of HSPB1 was associated with poor RFS of breast cancer patients. (F) High expression of HSPB1 was associated with poor DMFS of breast cancer patients.

Figure S2. HSPB1 knockdown attenuated breast cancer growth, migration, and invasion in vitro. (A) HSPB1 knockdown efficiency was confirmed by qRT-PCR and western blot in breast cancer cells. (B-D) MTT (B), colony formation (C), and EdU (D) assays were performed to evaluate the effect of HSPB1 knockdown on cell proliferative ability. Scale bar = 100 μm. (E) Wound healing assay was used to evaluate the effect of HSPB1 knockdown on the migration ability of breast cancer cells. Scale bar = 200 μm. (F) The migratory and invasive abilities of HSPB1-knockdown breast cancer cells were assessed by transwell assay. Scale bar = 100 μm. (G) Western blot showed the effect of HSPB1 knockdown on the expression of EMT-related proteins. (H) Representative images of morphological changes of

control and HSPB1 knockdown breast cancer cells. Scale bar = 100 μm. (I) Morphological changes of control and HSPB1 knockdown breast cancer cells assessed by phalloidin staining. Scale bar = 75 μm. (* P < 0.05, ** P < 0.01, *** P < 0.001)

Figure S3. HSPB1 was associated with chemoresistance of breast cancer. (A) MDA-MB-231 and MDA-MB-468 cells were treated with different concentrations of doxorubicin. The RNA and protein expression of HSPB1 was detected. (B) MDA-MB-231 and MDA-MB-468 cells were treated with doxorubicin (1 μM) and collected at the indicated time. The RNA and protein expression of HSPB1 was evaluated. (C) The expression of HSPB1 was upregulated in progressive breast cancer tissues compared to chemo-sensitive tissues. (* P < 0.05, ** P < 0.01, *** P < 0.001)

Figure S4. HSPB1 knockdown sensitized breast cancer cells to doxorubicin. (A) Inhibitory rates of MDA-MB-231 and MDA-MB-468 cells with or without HSPB1 knockdown was analyzed by MTT assay 48 h after treatment with different concentrations of doxorubicin. (B) HSPB1 knockdown efficiency was confirmed by qRT-PCR in MDA-MB-231/DOX cells. (C-E) MTT (C), EdU (D), and colony formation (E) assays were performed to evaluate the effect of HSPB1 knockdown on proliferative ability of MDA-MB-231/DOX cells. Scale bar = 100 μm. (F-G) Wound healing assay (Scale bar = 200 μm) (F), and transwell assay (Scale bar = 100 μm) (G) were used to assess the migratory and invasive abilities of MDA-MB-231/DOX cells.

(* $P < 0.05$, ** $P < 0.01$, *** $P < 0.001$)

Figure S5. The expression of HSPB1 was associated with ferroptosis. (A) MDA-MB-231 and MDA-MB-468 cells were treated with different concentrations of erastin for 48 h, and RNA and protein expression levels of HSPB1 were assayed by qRT-PCR or western blot. (B) Analysis of lipid ROS in MDA-MB-231 and MDA-MB-468 cells with or without HSPB1 overexpression after treatment with erastin (10 μM, 48 h). (C) Analysis of lipid ROS in MDA-MB-231 and MDA-MB-468 cells with or without HSPB1 knockdown after treatment with erastin (10 μM, 48 h). (D-E) Cell viability (D) and cellular ROS levels (E) in transfected MDA-MB-231 and MDA-MB-468 cells after indicated treatment. (F-H) Cell viability (F), ROS levels (G) and cellular MDA levels (H) were detected in MDA-MB-231/DOX cells with or without HSPB1 knockdown 48 h after treatment with 10 μM or 20 μM erastin plus either DMSO or 10 μM Ferrostatin-1. (* $P < 0.05$, ** $P < 0.01$, *** $P < 0.001$)

Figure S6. HSPB1 knockdown enhanced doxorubicin-induced ferroptosis in breast cancer cells. (A-B) ROS levels (A) and cellular MDA levels (B) were determined in MDA-MB-231, MDA-MB-468, and MDA-MB-231/DOX cells with or without HSPB1 knockdown 48 h after treatment with 1 μM doxorubicin. (C-E) Cell viability (C), cellular ROS levels (D), and cellular MDA levels (E) in MDA-MB-231 and MDA-MB-468 cells with or without HSPB1 knockdown after indicated treatment.

(F-H) Cell viability (F), cellular ROS levels (G), and cellular MDA levels (H) in MDA-MB-231/DOX cells with or without HSPB1 knockdown after treatment with doxorubicin plus either DMSO or Ferrostatin-1. (ns, no significance, * P < 0.05, ** P < 0.01, *** P < 0.001)

Figure S7. HSPB1 mediated the sensitivity of breast cancer cells to paclitaxel through suppressing ferroptosis. (A) Inhibitory rates of MDA-MB-231 and MDA-MB-468 cells with or without HSPB1 overexpression 48 h after treatment with different concentrations of paclitaxel. (B) Inhibitory rates of MDA-MB-231 and MDA-MB-468 cells with or without HSPB1 knockdown 48 h after treatment with different concentrations of paclitaxel. (C) Cellular ROS levels of MDA-MB-231 and MDA-MB-468 cells with or without HSPB1 overexpression 48 h after treatment with 10 μM paclitaxel. (D) Cellular ROS levels of MDA-MB-231 and MDA-MB-468 cells with or without HSPB1 knockdown 48 h after treatment with 10 μM paclitaxel. (E) Cell viability of MDA-MB-231 and MDA-MB-468 cells with or without HSPB1 overexpression 48 h after indicated treatment. (F) Cell viability of MDA-MB-231 and MDA-MB-468 cells with or without HSPB1 knockdown 48 h after indicated treatment. (G) Cellular ROS levels of MDA-MB-231 and MDA-MB-468 cells with or without HSPB1 overexpression 48 h after indicated treatment. (H) Cellular ROS levels of MDA-MB-231 and MDA-MB-468 cells with or without HSPB1 knockdown 48 h after indicated treatment. (ns, no significance, * P < 0.05, ** P < 0.01, *** P < 0.001)

Figure S8. HSPB1 knockdown attenuated activation of NF-κB signaling. (A) Western blot was performed using cell lysates of MDA-MB-231 and MDA-MB-468 cells with or without HSPB1 knockdown. (B-D) NF-κB transcriptional activity was determined by NF-κB activation reporter assay (B), western blot (C), and qRT-PCR (D). (E) After 24h of treatment with 1 μM doxorubicin, immunofluorescence was performed to detect the expression of HSPB1, Survivin, and IL6 in MDA-MB-231 and MDA-MB-468 cells with or without HSPB1 knockdown. Scale bar = 50 μm. (* P < 0.05, ** P < 0.01, *** P < 0.001)

Figure S9. Restoring NF-κB activity partially reversed the inhibited effect of HSPB1 knockdown in breast cancer cells. MDA-MB-231 and MDA-MB-468 cells were transfected with negative control siRNA (Ctrl-siRNA), ctrl-siRNA + si-HSPB1, or si-HSPB1 + si-Ikβ-α for 48 h. (A) Wound healing assay was performed to determined cell migration. Scale bar = 200 μm. (B) The MTT assay was used to analyze the viability of transfected cells following 10 μM erastin plus either DMSO or 10 μM Fer-1 treatment. (* P < 0.05, ** P < 0.01, *** P < 0.001)

Figure S10. The effect of HSPB1 on the behaviors of THP1. (A) The correlation between HSPB1 expression and the level of macrophage infiltration in tumor microenvironment was analyzed. (B-C) The supernatant of breast cancer cells with or without HSPB1 knockdown was added in the upper chamber or lower chamber, and

transwell assay was used to detect the migration of macrophages (B) or chemotaxis (C). (D-E) The conditioned medium from HSPB1 overexpressing cells or control cells was added in the upper chamber or lower chamber, and transwell assay was used to detect the migration of macrophages (D) or chemotaxis (E). Scale bar = 100 μm. (* $P < 0.05$, ** $P < 0.01$, *** $P < 0.001$)

Figure S1

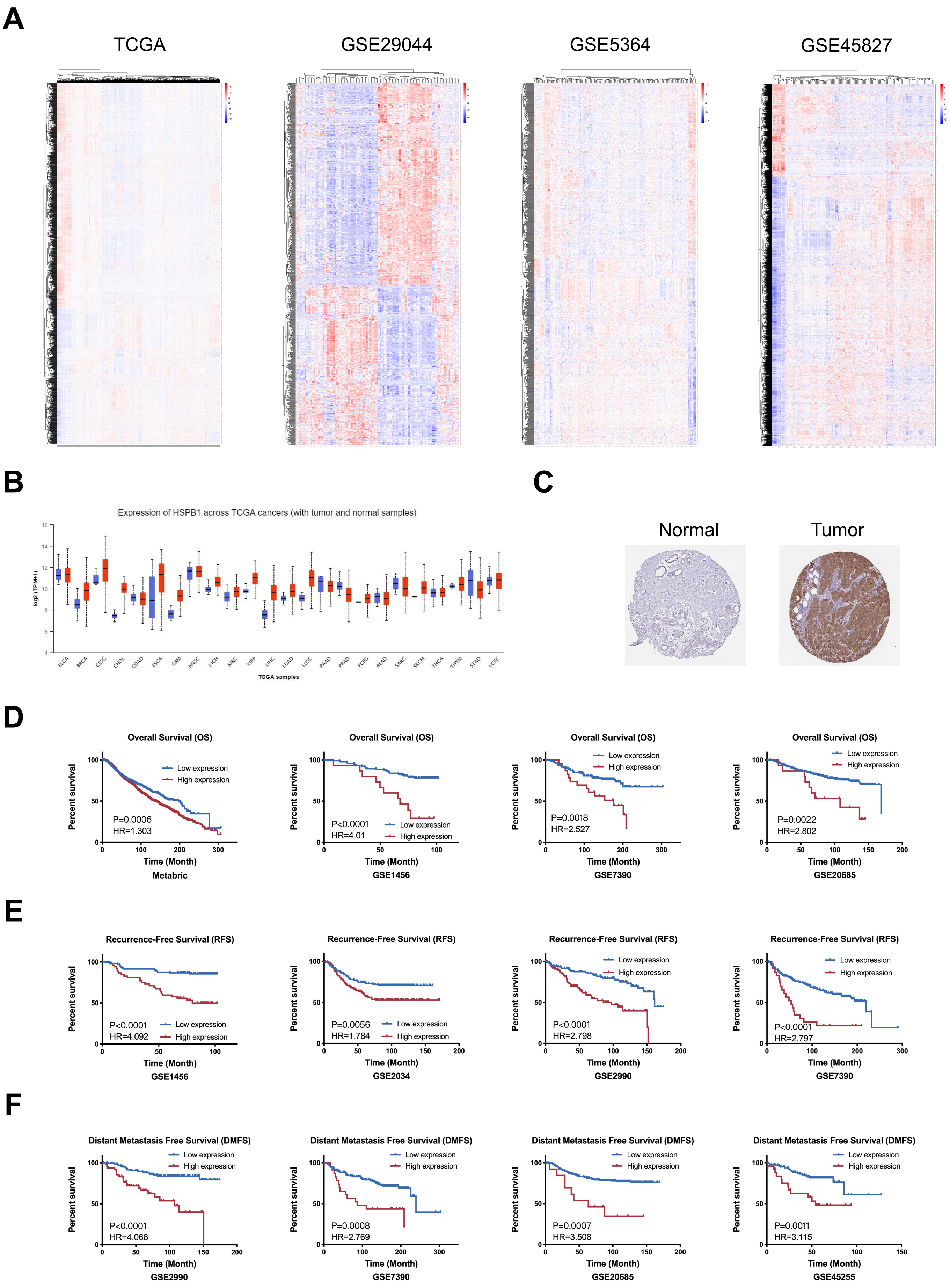
A
TCGA
GSE29044
GSE5364
GSE45827
B
Expression of HSPB1 across TCGA cancers (with tumor and normal samples)
log2 (TPM+1)
TCGA samples
BLCA
BRCA
CESC
CHOL
COAD
ESCA
GBM
HNSC
KICH
KIRC
KIRP
LIHC
LUAD
LUSC
PAAD
PRAD
PCPG
READ
SARC
SKCM
THCA
THYM
STAD
UCEC
C
Normal
Tumor
D
Overall Survival (OS)
Percent survival
Time (Month)
Low expression
High expression
P=0.0006
HR=1.303
Metabric
P<0.0001
HR=4.01
GSE1456
P=0.0018
HR=2.527
GSE7390
P=0.0022
HR=2.802
GSE20685
E
Recurrence-Free Survival (RFS)
P<0.0001
HR=4.092
GSE1456
P=0.0056
HR=1.784
GSE2034
P<0.0001
HR=2.798
GSE2990
P<0.0001
HR=2.797
GSE7390
F
Distant Metastasis Free Survival (DMFS)
P<0.0001
HR=4.068
GSE2990
P=0.0008
HR=2.769
GSE7390
P=0.0007
HR=3.508
GSE20685
P=0.0011
HR=3.115
GSE45255

Figure S2

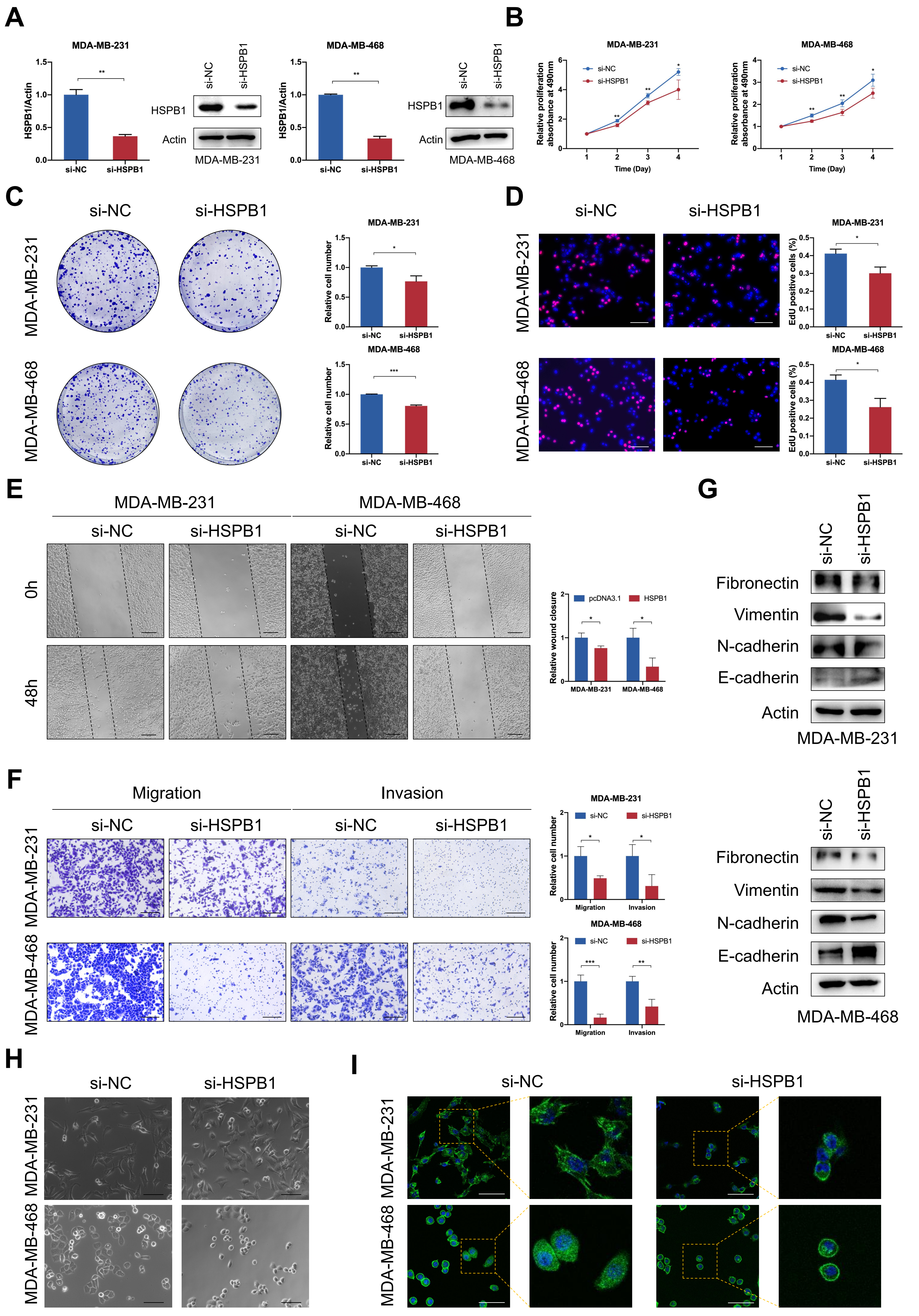

A
MDA-MB-231
HSPB1/Actin
si-NC
si-HSPB1
HSPB1
Actin
MDA-MB-468
B
Relative proliferation absorbance at 490nm
Time (Day)
C
Relative cell number
D
EdU positive cells (%)
E
0h
48h
Relative wound closure
pcDNA3.1
HSPB1
F
Migration
Invasion
G
Fibronectin
Vimentin
N-cadherin
E-cadherin
H
I

Figure S3

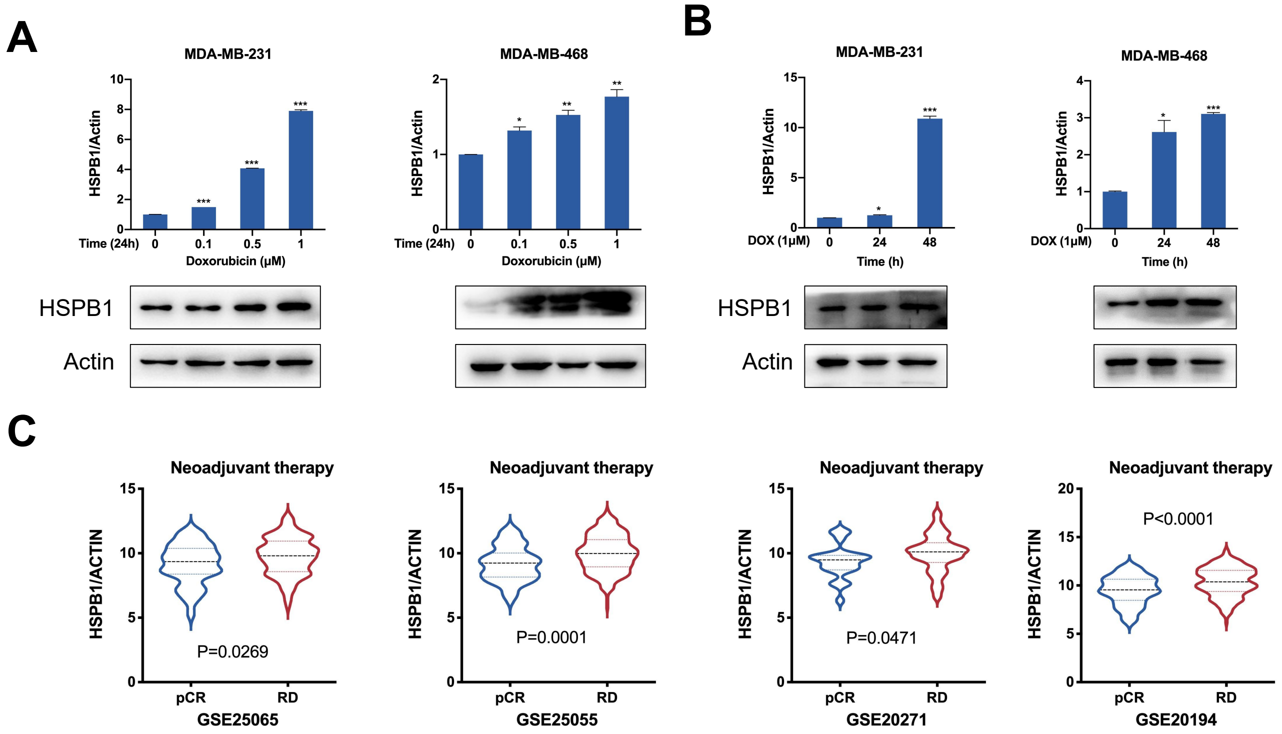
A
MDA-MB-231
HSPB1/Actin
Time (24h)
Doxorubicin (μM)
MDA-MB-468
HSPB1
Actin
B
DOX (1μM)
Time (h)
C
Neoadjuvant therapy
HSPB1/ACTIN
P=0.0269
pCR
RD
GSE25065
P=0.0001
GSE25055
P=0.0471
GSE20271
P<0.0001
GSE20194

Figure S4

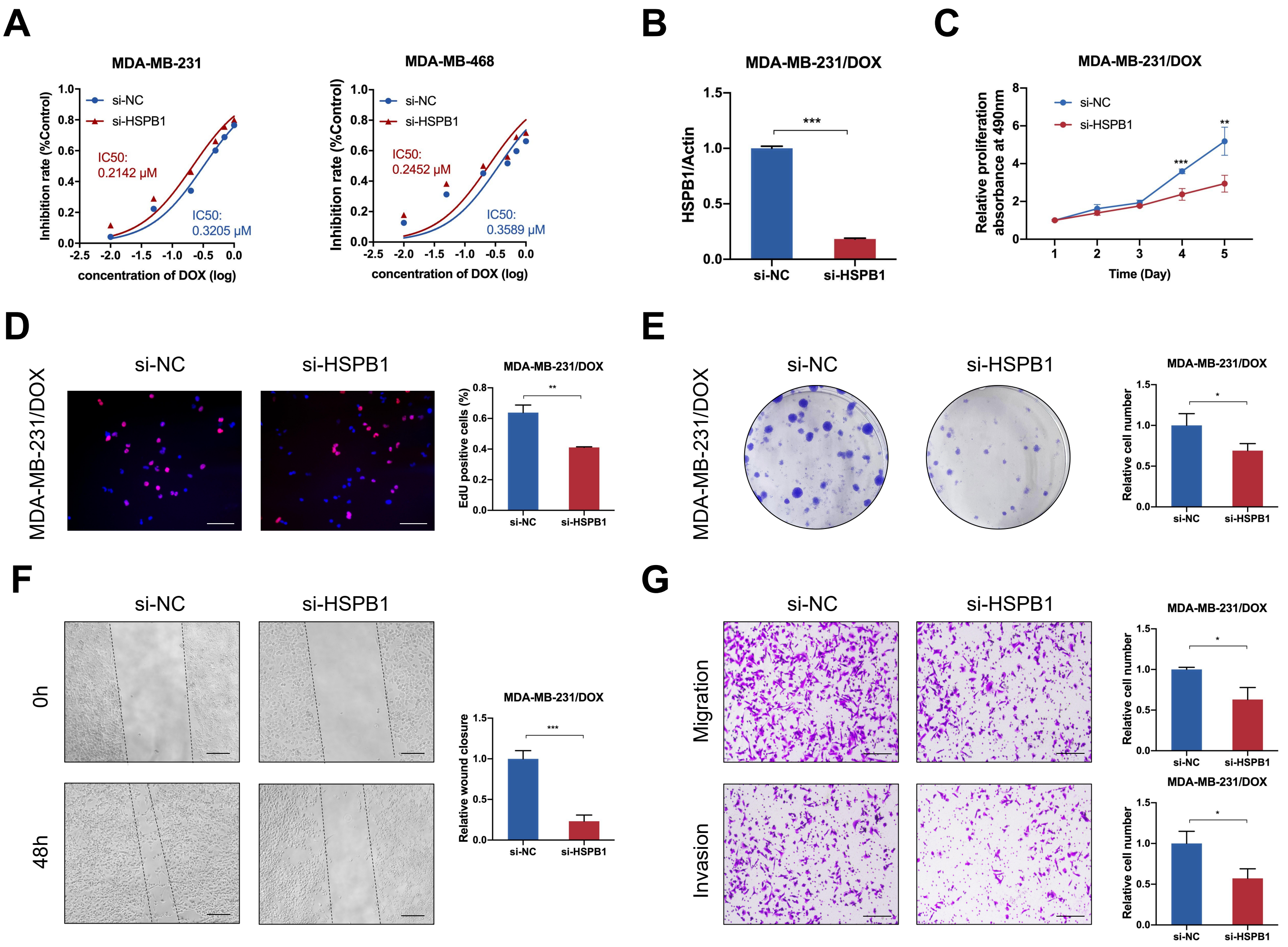

A
MDA-MB-231
MDA-MB-468
si-NC
si-HSPB1
IC50: 0.2142 μM
IC50: 0.3205 μM
IC50: 0.2452 μM
IC50: 0.3589 μM
Inhibition rate (%Control)
concentration of DOX (log)
B
MDA-MB-231/DOX
HSPB1/Actin
C
Relative proliferation absorbance at 490nm
Time (Day)
D
EdU positive cells (%)
E
Relative cell number
F
0h
48h
Relative wound closure
G
Migration
Invasion

Figure S5

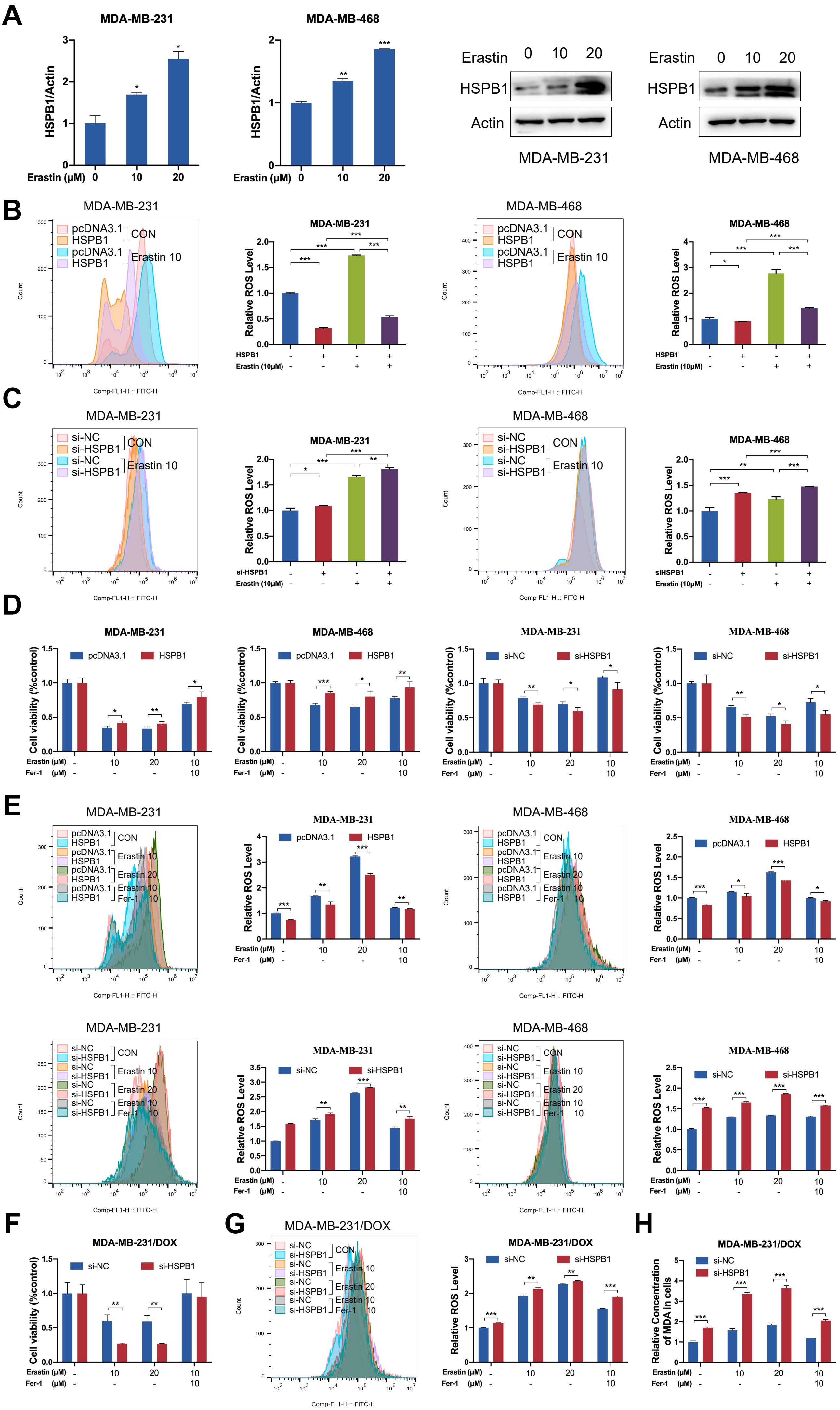
A
MDA-MB-231
MDA-MB-468
HSPB1/Actin
Erastin (μM) 0 10 20
Erastin 0 10 20
HSPB1
Actin
MDA-MB-231
MDA-MB-468
B
pcDNA3.1
HSPB1
CON
Erastin 10
Relative ROS Level
Erastin (10μM)
Comp-FL1-H :: FITC-H
Count
C
si-NC
si-HSPB1
siHSPB1
D
Cell viability (%control)
Fer-1 (μM)
E
Erastin 20
F
MDA-MB-231/DOX
G
H
Relative Concentration of MDA in cells

Figure S6

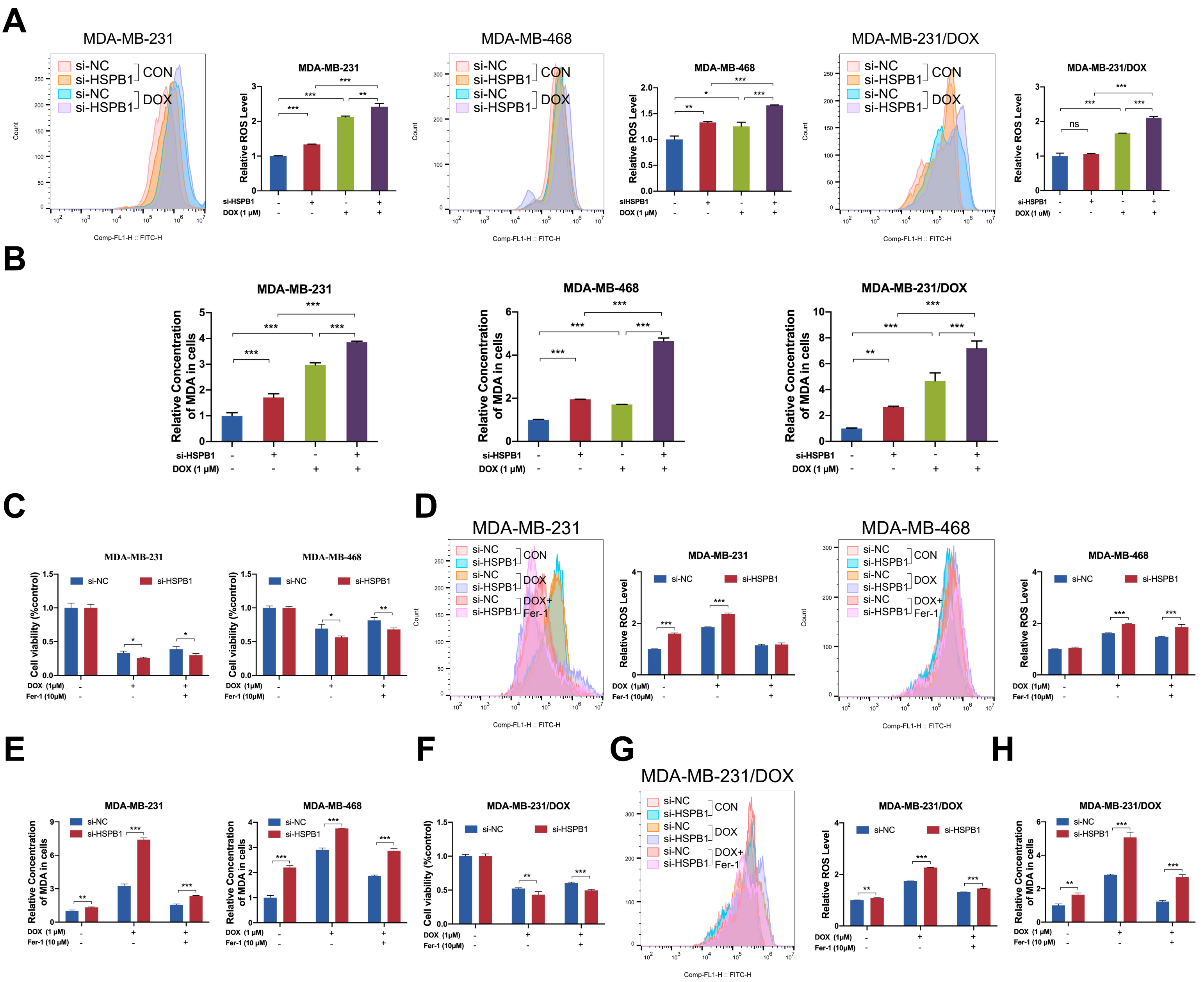

A
MDA-MB-231
MDA-MB-468
MDA-MB-231/DOX
si-NC
si-HSPB1
CON
DOX
Relative ROS Level
si-HSPB1
DOX (1 μM)
Count
Comp-FL1-H :: FITC-H
B
Relative Concentration of MDA in cells
C
Cell viability (%control)
Fer-1 (10μM)
D
DOX+ Fer-1
E
F
G
H

Figure S7

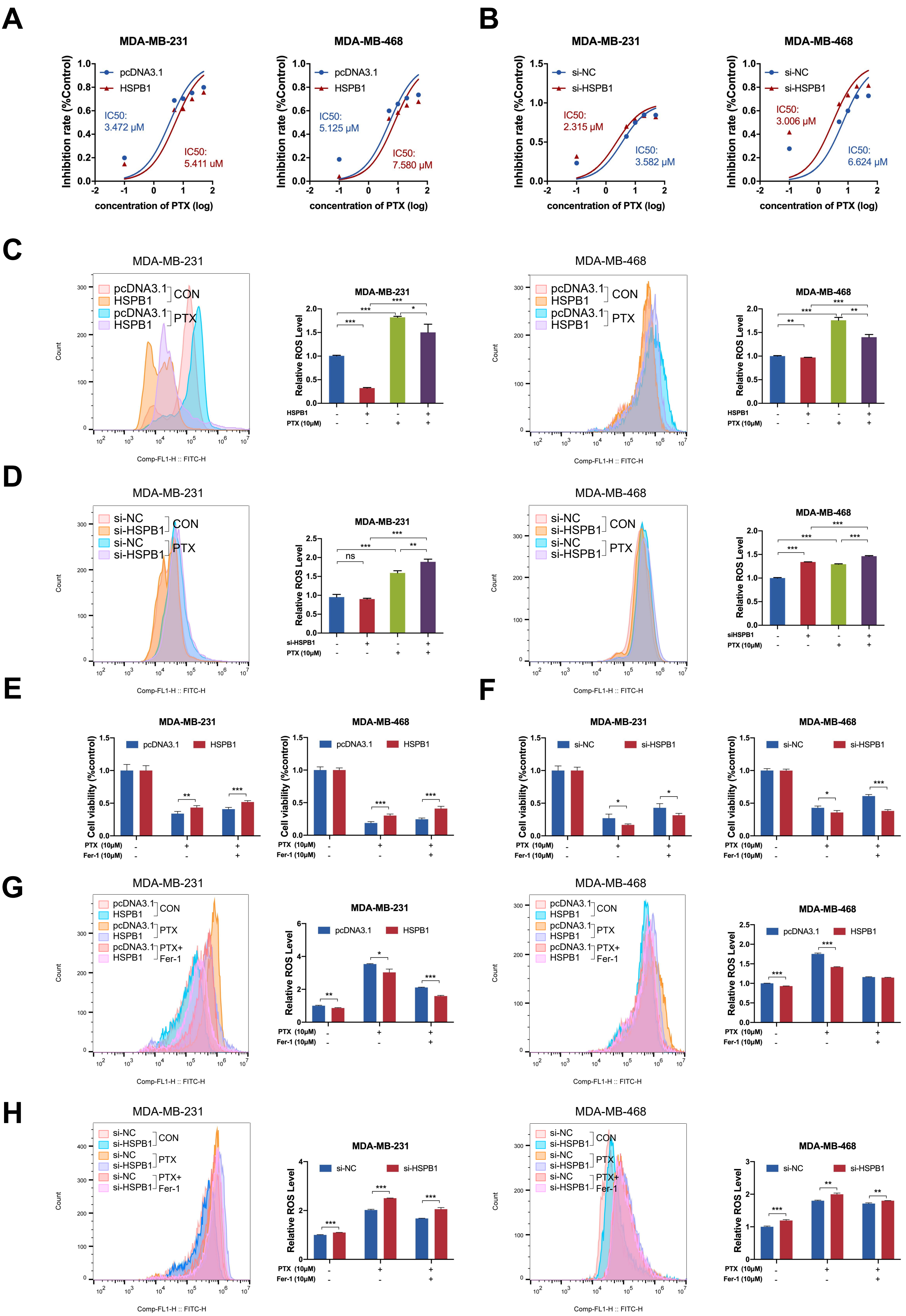

A
B
C
D
E
F
G
H
MDA-MB-231
MDA-MB-468
pcDNA3.1
HSPB1
si-NC
si-HSPB1
IC50: 3.472 μM
IC50: 5.411 uM
IC50: 5.125 μM
IC50: 7.580 μM
IC50: 2.315 μM
IC50: 3.582 μM
IC50: 3.006 μM
IC50: 6.624 μM
Inhibition rate (%Control)
concentration of PTX (log)
CON
PTX
PTX+ Fer-1
Count
Comp-FL1-H :: FITC-H
Relative ROS Level
Cell viability (%control)
HSPB1
siHSPB1
PTX (10μM)
Fer-1 (10μM)
ns

Figure S8

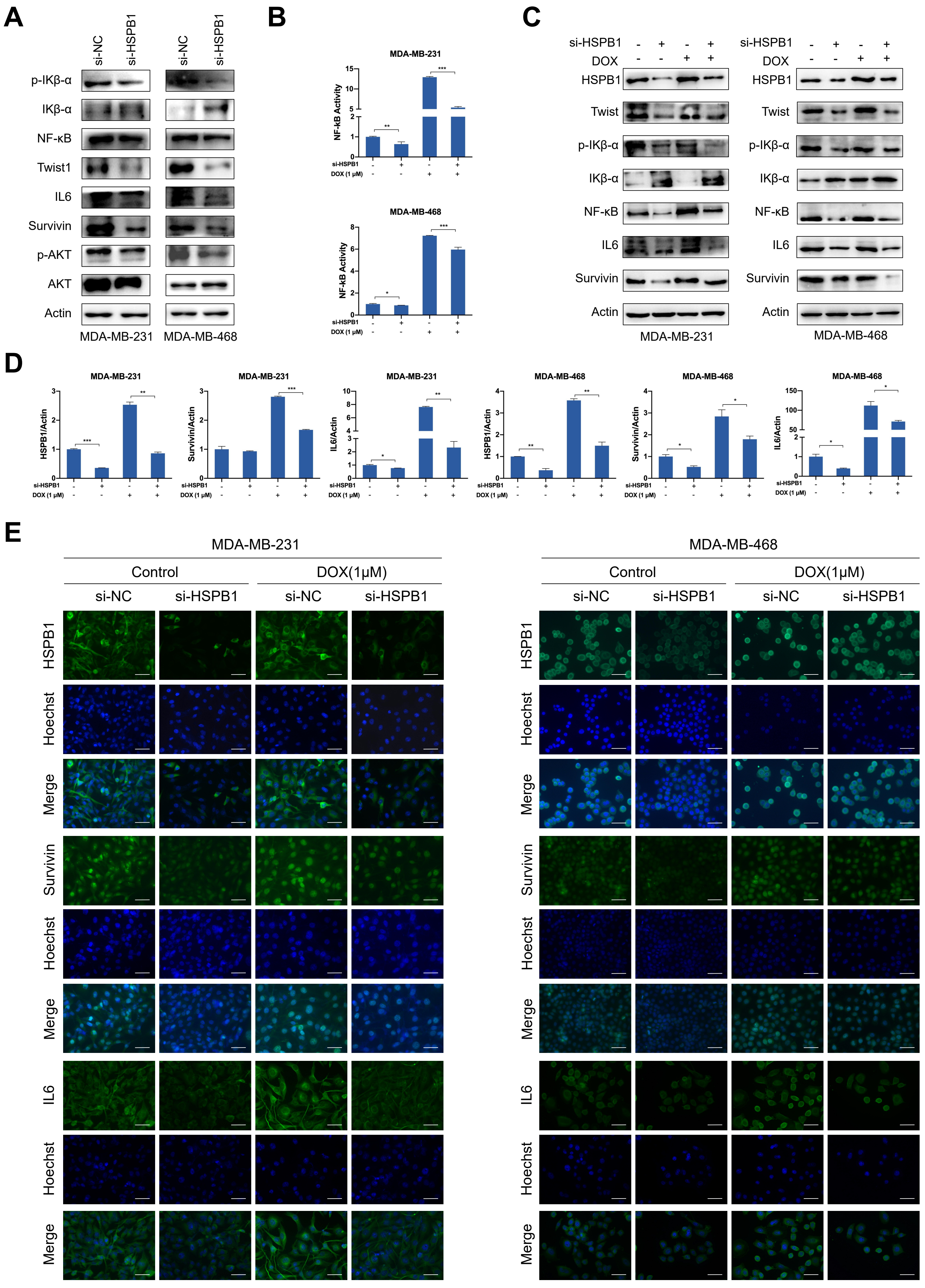
A
si-NC
si-HSPB1
p-IKβ-α
IKβ-α
NF-κB
Twist1
IL6
Survivin
p-AKT
AKT
Actin
MDA-MB-231
MDA-MB-468
B
MDA-MB-231
NF-κB Activity
si-HSPB1
DOX (1 μM)
MDA-MB-468
C
si-HSPB1
DOX
HSPB1
Twist
p-IKβ-α
IKβ-α
NF-κB
IL6
Survivin
Actin
MDA-MB-231
MDA-MB-468
D
MDA-MB-231
HSPB1/Actin
Survivin/Actin
IL6/Actin
MDA-MB-468
E
MDA-MB-231
MDA-MB-468
Control
DOX(1μM)
si-NC
si-HSPB1
HSPB1
Hoechst
Merge
Survivin
IL6

Figure S9

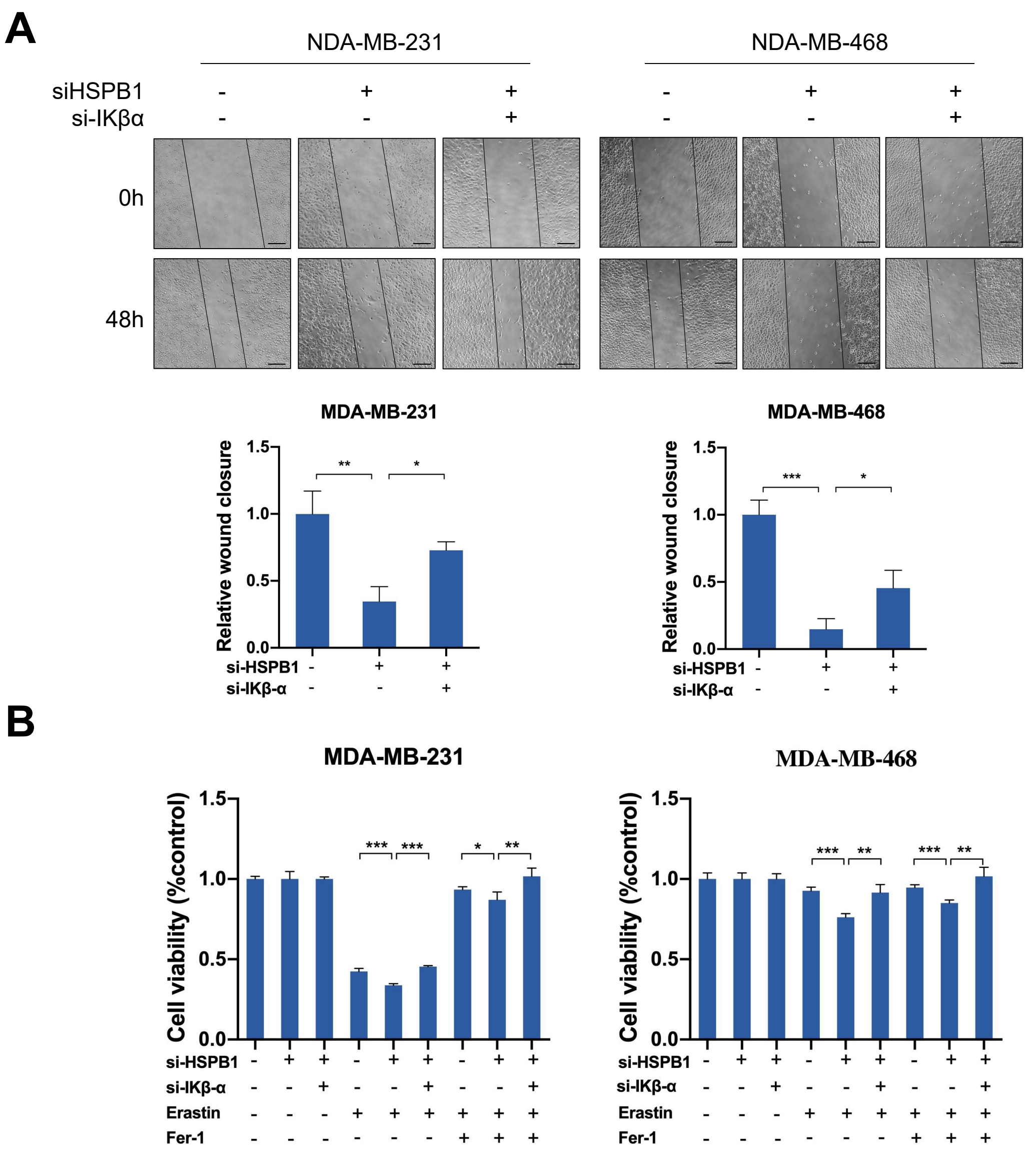
A
NDA-MB-231
NDA-MB-468
siHSPB1
si-IKβα
0h
48h
MDA-MB-231
MDA-MB-468
Relative wound closure
si-HSPB1
si-IKβ-α
B
MDA-MB-231
MDA-MB-468
Cell viability (%control)
si-HSPB1
si-IKβ-α
Erastin
Fer-1

Figure S10

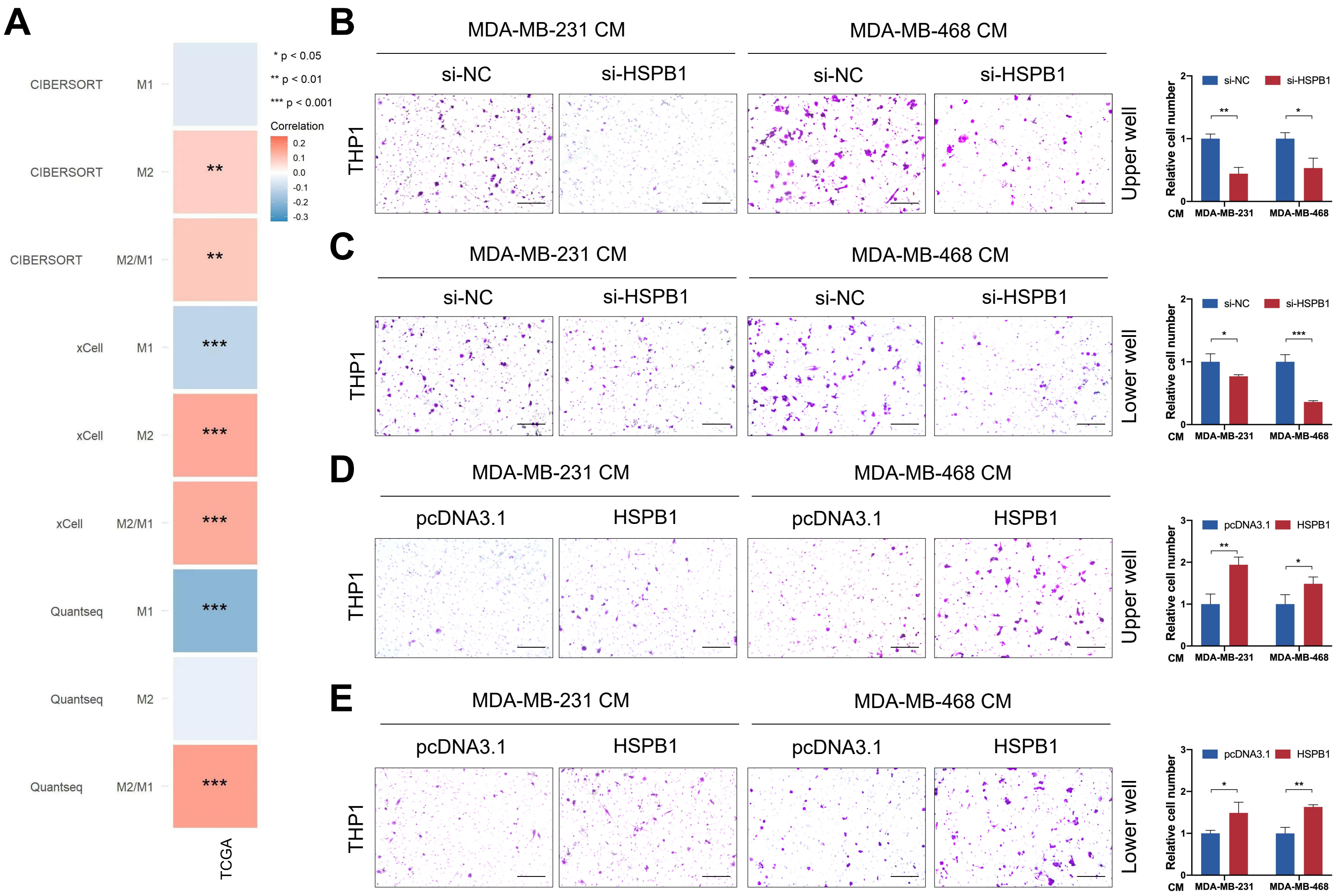
A
CIBERSORT M1
CIBERSORT M2
CIBERSORT M2/M1
xCell M1
xCell M2
xCell M2/M1
Quantseq M1
Quantseq M2
Quantseq M2/M1
TCGA
* p < 0.05
** p < 0.01
*** p < 0.001
Correlation
B
C
D
E
MDA-MB-231 CM
MDA-MB-468 CM
si-NC
si-HSPB1
pcDNA3.1
HSPB1
THP1
Upper well
Lower well
Relative cell number
CM
MDA-MB-231
MDA-MB-468

Supplementary Tables

Table S1. Correlations between HSPB1 expression and clinicopathologic features in 131 breast cancer patients.

Characteristics	HSPB1-low (n=88)	HSPB1-high (n=43)	p value
Age			p=0.291
＜59	67 (76.14%)	29 (67.44%)	
≥59	21 (23.86%)	14 (32.56%)	
Tumor stage			p=0.806
≤T1	44 (50.00%)	20 (46.51%)	
＞T1	43 (48.86%)	23 (53.49%)	
unknown	1 (1.14%)	0 (0.00%)	
LN metastasis			p=0.166
0	61 (69.32%)	23 (53.48%)	
1-3	16 (18.18%)	10 (23.26%)	
＞3	11 (12.50%)	10 (23.26%)	
Distant metastasis			**p＜0.001**
No	76 (86.36%)	21 (48.84%)	
Yes	7 (7.95%)	6 (13.95%)	
unknown	5 (5.68%)	16 (37.21%)	
Histologic grade			p=0.166
G1	7 (7.95%)	0 (0.00%)	
G2	46 (52.27%)	20 (46.51%)	
G3	27 (30.68%)	19 (44.19%)	
unknown	8 (9.09%)	4 (9.30%)	
ER status			p=0.721
negative	52 (59.09%)	24 (55.81%)	
positive	36 (40.91%)	19 (44.19%)	
PR status			p=0.463
negative	55 (62.50%)	24 (55.81%)	
positive	33 (37.50%)	19 (44.19%)	
HER-2 status			p=0.154
negative	81 (92.05%)	35 (81.40%)	
positive	1 (1.14%)	2 (4.65%)	
unknown	6 (6.81%)	6 (13.95%)	
Ki67 expression			p=0.052
low	22 (25.00%)	6 (13.95%)	
high	66 (75.00%)	35 (81.40%)	
unknown	0 (0.00%)	2 (4.65%)	

Abbreviation: LN=lymph nodes; ER=estrogen receptor; PR=progesterone receptor; HER-2: human epidermal growth factor receptor-2; p value＜0.05 marked in bond font to show statistical significant.

Table S2. Univariate and multivariate Cox regression analyses for OS of 131 breast cancer patients.

	Univariate analysis		Multivariate analysis	
	HR (95% CI)	p value	HR (95% CI)	p value
Age (≥59 vs. ＜59)	3.028 (1.259-7.281)	**0.013**	2.704 (1.123-6.510)	**0.026**
Histologic grade				
G2 vs. G1	9317.972 (0.000-3.001E+78)	0.917	-	-
G3 vs. G1	8650.070 (0.000-2.787E+78)	0.918	-	-
Unknown vs. G1	8523.247 (0.000-2.758E+78)	0.918	-	-
Tumor size (＞T1 vs.≤T1+unknown)	1.313 (0.543-3.172)	0.545	-	-
LN metastasis				
1-3 vs. 0	1.983 (0.580-6.780)	0.275	2.067 (0.590-7.241)	0.256
＞3 vs. 0	8.050 (2.902-22.326)	**＜0.001**	6.213 (2.165-17.827)	**0.001**
Distant metastasis (Yes vs. No+unknown)	1.579 (0.462-5.394)	0.466	-	-
ER status (pos vs. neg)	1.017 (0.400-2.584)	0.973	-	-
PR status (pos vs. neg)	0.878 (0.339-2.272)	0.788	-	-
HER-2 status (pos vs. neg+unknown)	2.433 (0.322-18.388)	0.389	-	-
Ki67 expression (pos vs. neg+unknown)	1.185 (0.395-3.559)	0.762	-	-
HSPB1 expression (High vs. Low)	7.042 (2.556-19.398)	**＜0.001**	5.520 (1.984-15.355)	**0.001**

Abbreviation: 95% CI=95% confidence interval; HR=hazard ratio; LN=lymph nodes; ER=estrogen receptor; PR=progesterone receptor; HER-2: human epidermal growth factor receptor-2; p value＜0.05 marked in bond font to show statistical significant.

Table S3. Univariate and multivariate Cox regression analyses for DFS of 131 breast cancer patients.

	Univariate analysis		Multivariate analysis	
	HR (95% CI)	p value	HR (95% CI)	p value
Age (≥59 vs. ＜59)	2.412 (1.169-4.975)	**0.017**	2.171 (1.038-4.540)	**0.039**
Histologic grade				
G2 vs. G1	9683.222 (0.000-1.018E+69)	0.904	-	-
G3 vs. G1	9420.873 (0.000-9.911E+68)	0.905	-	-
Unknown vs. G1	5068.560 (0.000-5.361E+68)	0.911	-	-
Tumor size (＞T1 vs. ≤T1+unknown)	1.638 (0.788-3.407)	0.187	-	-
LN metastasis				
1-3 vs. 0	2.956 (1.162-7.520)	**0.023**	3.677 (1.348-10.033)	**0.011**
＞3 vs. 0	10.279 (4.180-25.280)	**＜0.001**	7.422 (2.808-19.617)	**＜0.001**
Distant metastasis (Yes vs. No+unknown)	6.412 (3.105-13.241)	**＜0.001**	2.726 (1.284-5.789)	**0.009**
ER status (pos vs. neg)	1.531 (0.683-3.432)	0.301	-	-
PR status (pos vs. neg)	1.396 (0.625-3.120)	0.415	-	-
HER-2 status (pos vs. neg+unknown)	2.242 (0.298-16.885)	0.433	-	-
Ki67 expression (pos vs. neg+unknown)	0.932 (0.398-2.183)	0.871	-	-
HSPB1 expression (High vs. Low)	5.753 (2.631-12.579)	**＜0.001**	4.279 (1.886-9.709)	**0.001**

Abbreviation: 95% CI=95% confidence interval; HR=hazard ratio; LN=lymph nodes; ER=estrogen receptor; PR=progesterone receptor; HER-2: human epidermal growth factor receptor-2; p value＜0.05 marked in bond font to show statistical significant.

Table S4. SiRNA used for transfection.

Name	Sense （5'-3'）	Antisense （5'-3'）
si-HSPB1	GGUGCUUCACGCGGAAAUA	UAUUUCCGCGUGAAGCACC
si-Ikβ-α	CCGAGACUUUCGAGGAAAU	AUUUCCUCGAAAGUCUCGG
si-NC	UUCUCCGAACGUGUCACGU	ACGUGACACGUUCGGAGAA

Table S5. Primers used for qRT-PCR.

Gene	Forward （5'-3'）	Reverse （5'-3'）
HSPB1	AAGGATGGCGTGGTGGAGA	GAGGAGGAAACTTGGGTGGG
IL6	CAGCCACTCACCTCTTCAG	TGCCAGTGCCTCTTTGCTG
Survivin	AACTGGCCCTTCTTGGAG	GGTCGTCATCTGGCTCCC
Ikβ-α	TCCACTCCATCCTGAAGGCTAC	CAAGGACACCAAAAGCTCCACG
Actin	CACTGTGCCCATCTACGAG	AATGTCACGCACGATTTCC

Table S6. Antibodies used in the experiments.

Antigen	Supplier	Catalog #	Application
Actin	Proteintech	60008-1-Ig	IB (1:2 000)
Fibronectin	Proteintech	15613-1-AP	IB (1:1 000)
N-cadherin	Proteintech	YT2988	IB (1:1 000)
E-cadherin	Proteintech	20874-1-AP	IB (1:1 000)
Vimentin	Cell Signaling Technology	5741	IB (1:1 000)
HSPB1	Proteintech	18284-1-AP	IB (1:1 000) IF (1:200) IHC (1:200)
Twist	Proteintech	25465-1-AP	IB (1:1 000)
p-IKβ-α	Cell Signaling Technology	5209S	IB (1:1 000)
IKβ-α	Proteintech	10268-1-AP	IB (1:1 000) IF (1:200) IHC (1:200)
NF-κB	Cell Signaling Technology	8242	IB (1:1 000) IF (1:200) IHC (1:200)
IL6	Proteintech	21865-1-AP	IB (1:1 000) IF (1:200) IHC (1:200)
Survivin	Proteintech	10508-1-AP	IB (1:1 000) IF (1:200) IHC (1:200)
p-AKT	Proteintech	28731-1-AP	IB (1:1 000)
AKT	Proteintech	10176-2-AP	IB (1:1 000)
Lamin A/C	Proteintech	10298-1-AP	IB (1:1 000)
Flag	Sigma-Aldrich	F2555	IB (1:1 000)
HA	Sigma-Aldrich	H6908	IB (1:1 000)
Ki67	Proteintech	27309-1-AP	IHC(1:500)
HRP-anti-mouse	Cell Signaling Technology	7076	IB (1:5 000)
HRP-anti-rabbit	Cell Signaling Technology	7074	IB (1:5 000)
