# Supplementary material for: HSPB1 facilitates chemoresistance through inhibiting ferroptotic cancer cell death and regulating NF-κB signaling pathway in breast cancer
Source: Cell Death Dis. 2023 Jul 15;14(7):434. doi: 10.1038/s41419-023-05972-0 (PMC10349816; doi:10.1038/s41419-023-05972-0)
Supplement: Supplementary file 2 — Original western blot [file 41419_2023_5972_MOESM2_ESM.pptx]

## Slide 1
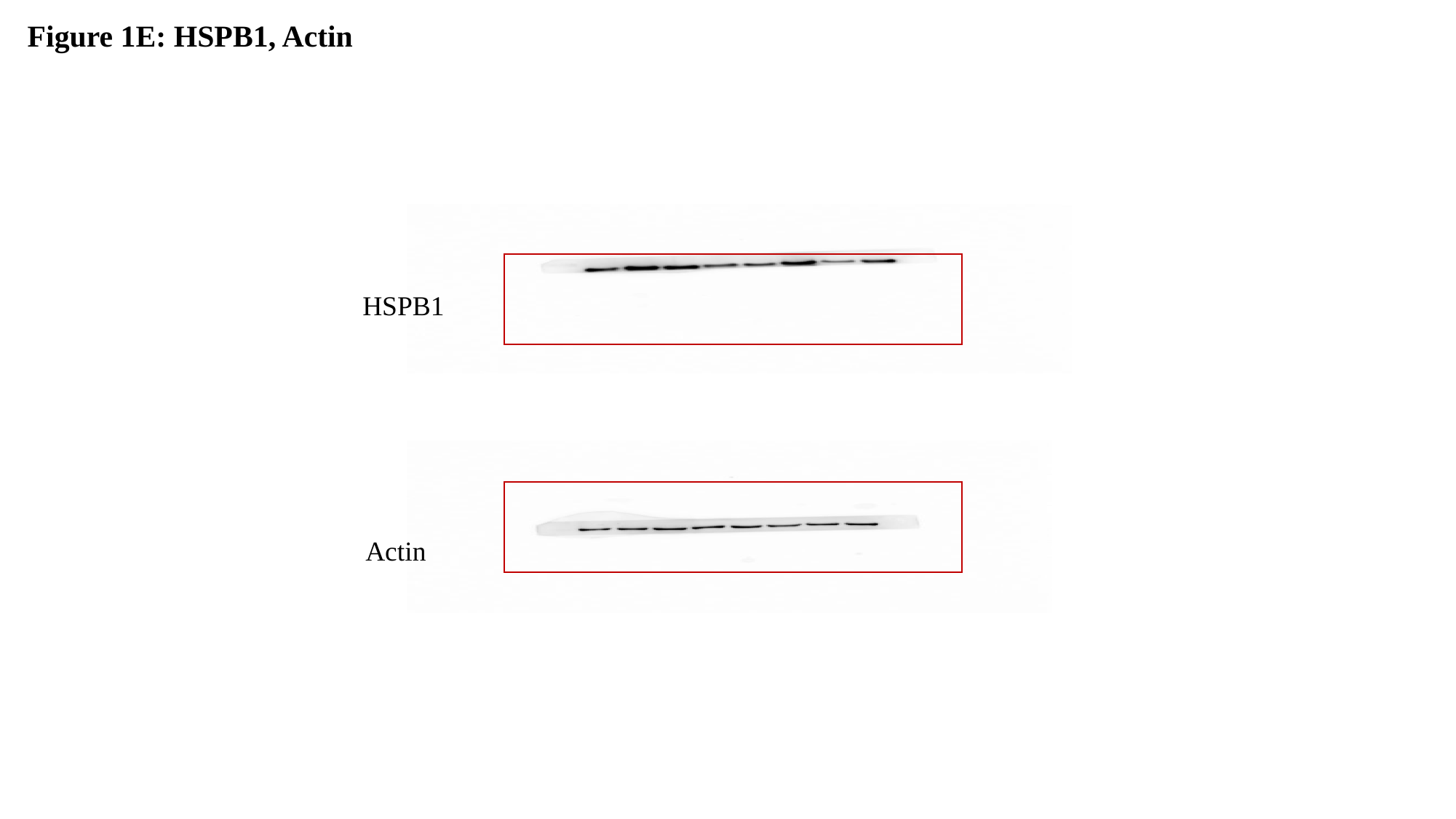

Figure 1E: HSPB1, Actin
HSPB1
Actin

## Slide 2
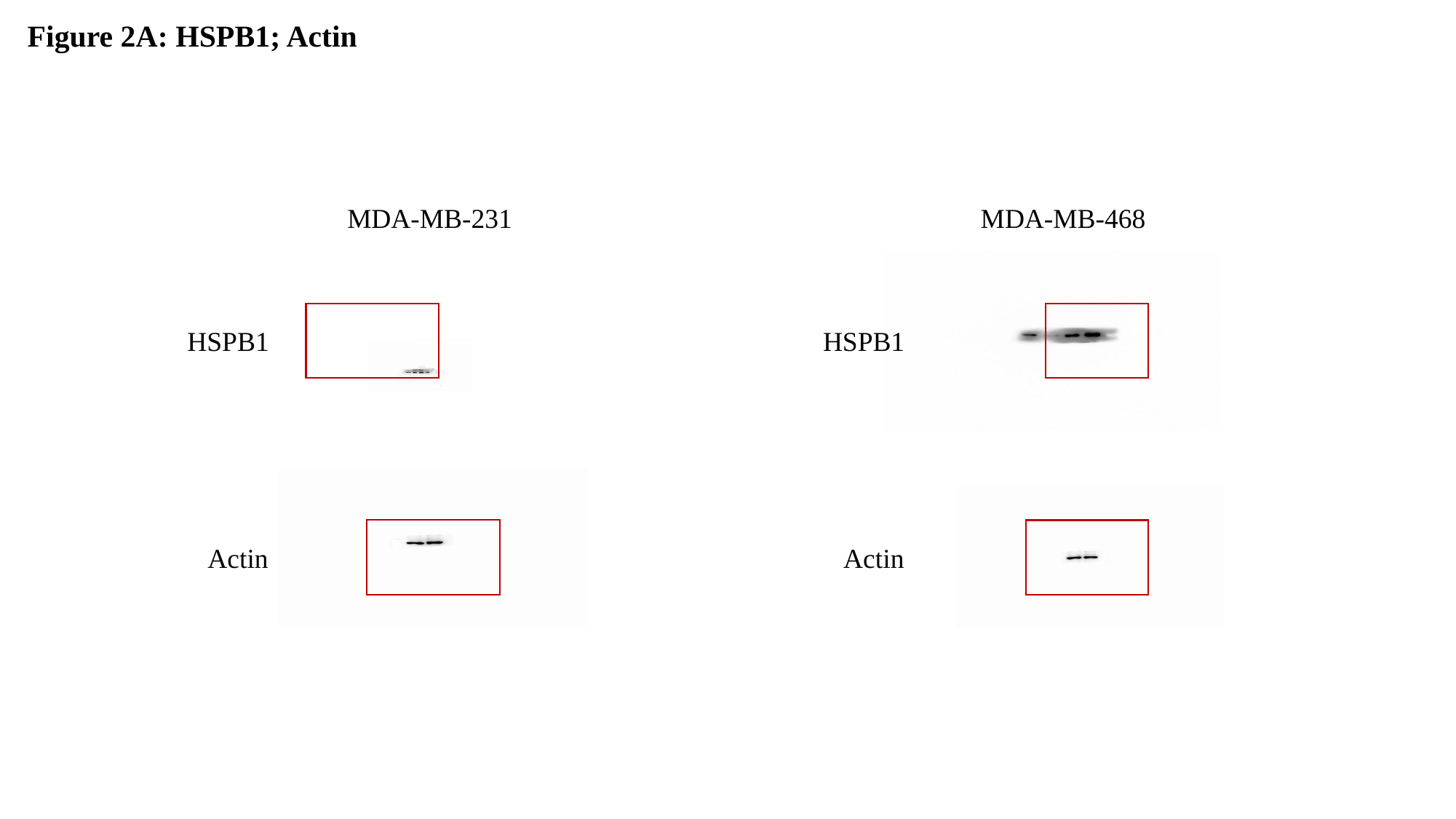

Figure 2A: HSPB1; Actin
MDA-MB-231
MDA-MB-468
HSPB1
HSPB1
Actin
Actin

## Slide 3
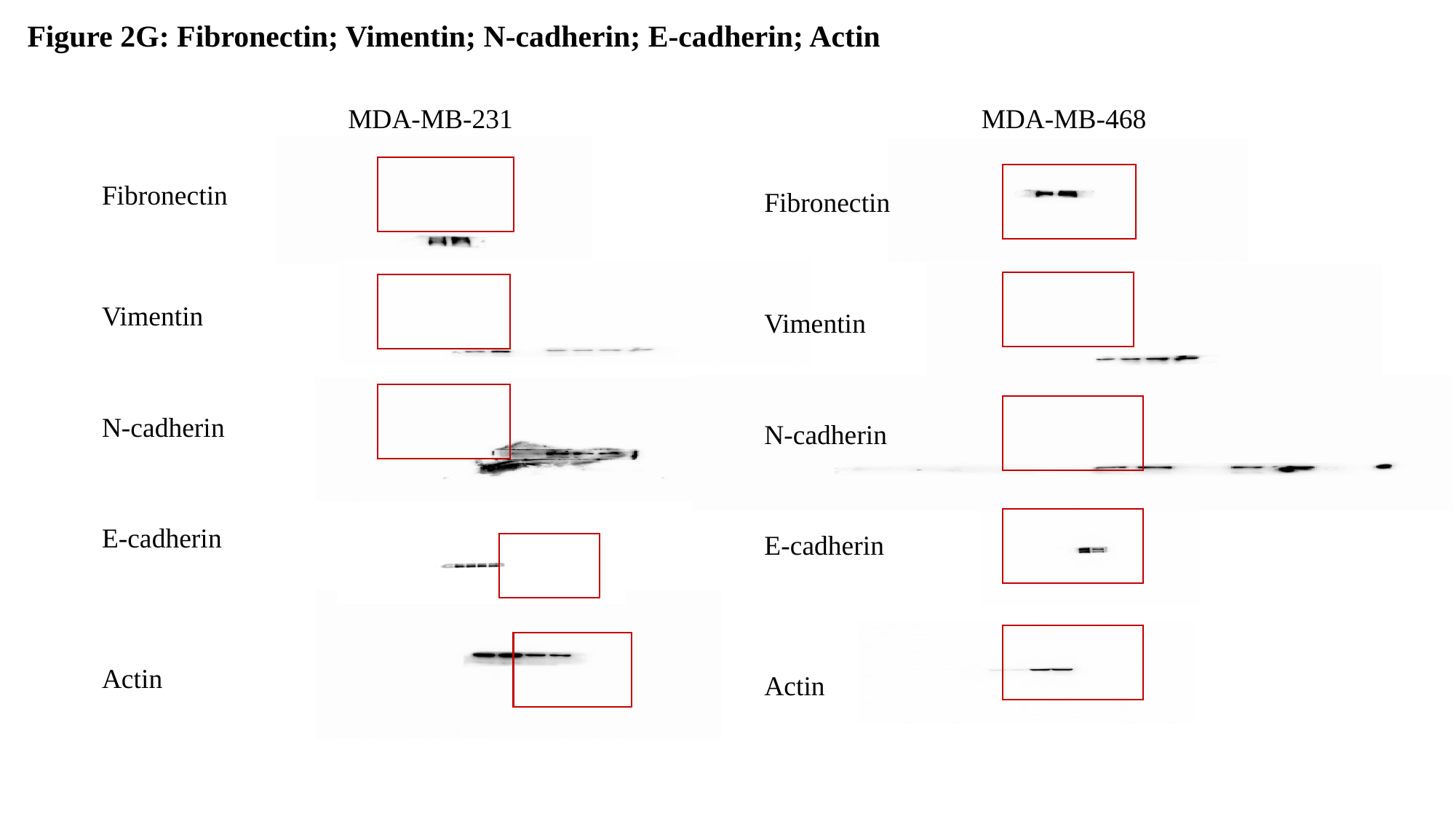

Figure 2G: Fibronectin; Vimentin; N-cadherin; E-cadherin; Actin
MDA-MB-231
MDA-MB-468
Fibronectin
Fibronectin
Vimentin
Vimentin
N-cadherin
N-cadherin
E-cadherin
E-cadherin
Actin
Actin

## Slide 4
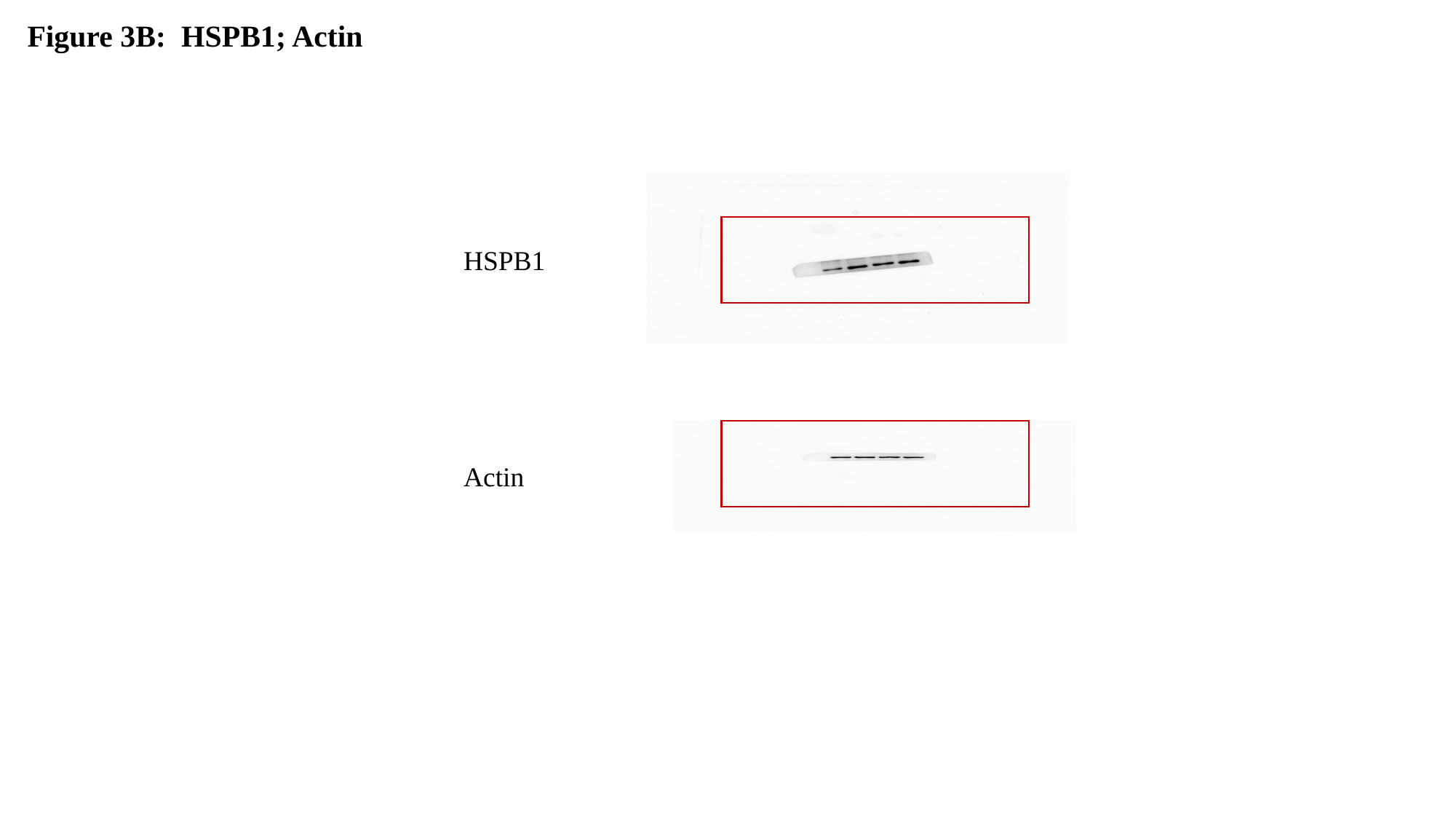

Figure 3B: HSPB1; Actin
HSPB1
Actin

## Slide 5
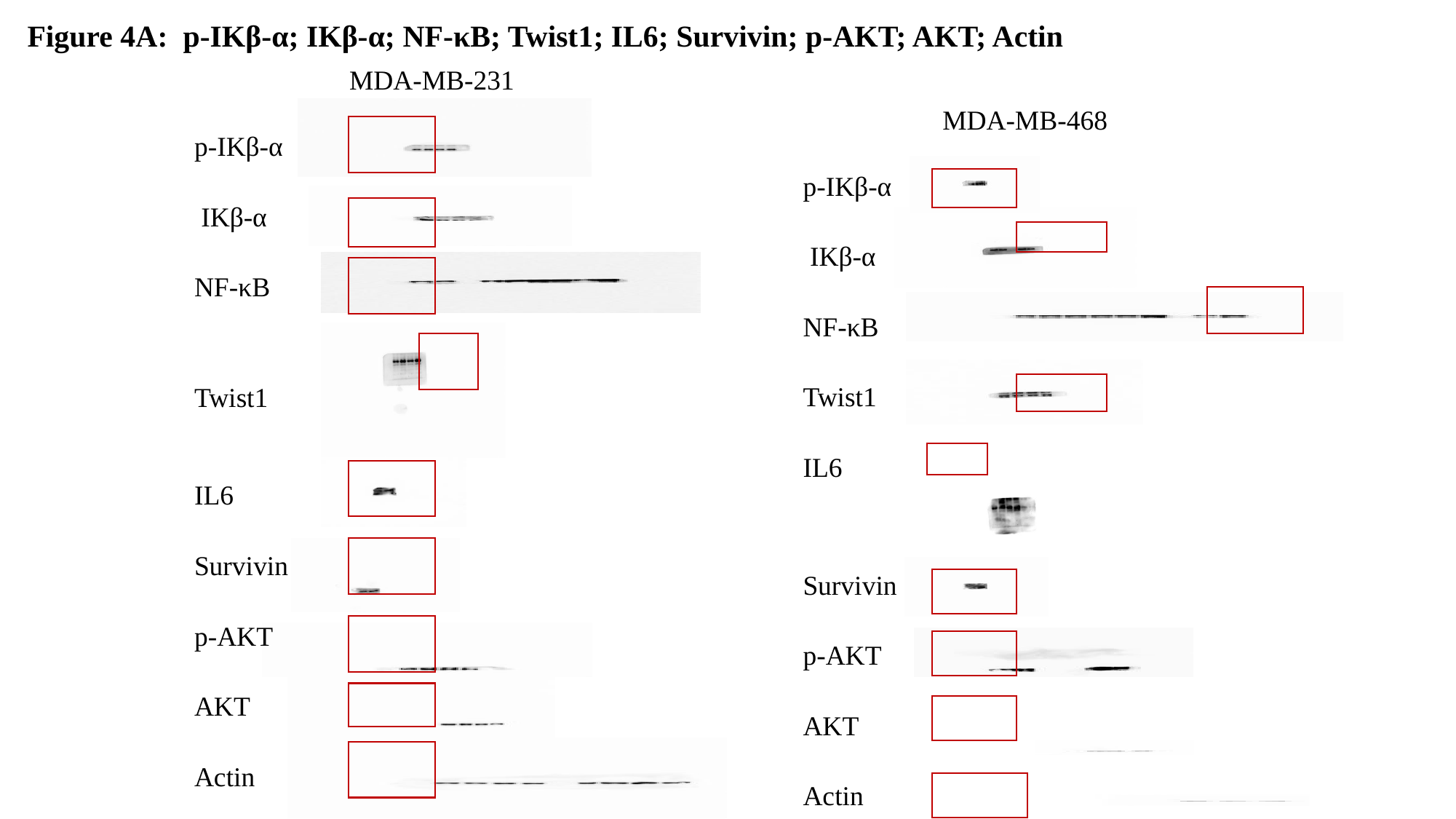

Figure 4A: p-IKβ-α; IKβ-α; NF-κB; Twist1; IL6; Survivin; p-AKT; AKT; Actin
MDA-MB-231
MDA-MB-468
p-IKβ-α
p-IKβ-α
 IKβ-α
 IKβ-α
NF-κB
NF-κB
Twist1
Twist1
IL6
IL6
Survivin
Survivin
p-AKT
p-AKT
AKT
AKT
Actin
Actin

## Slide 6
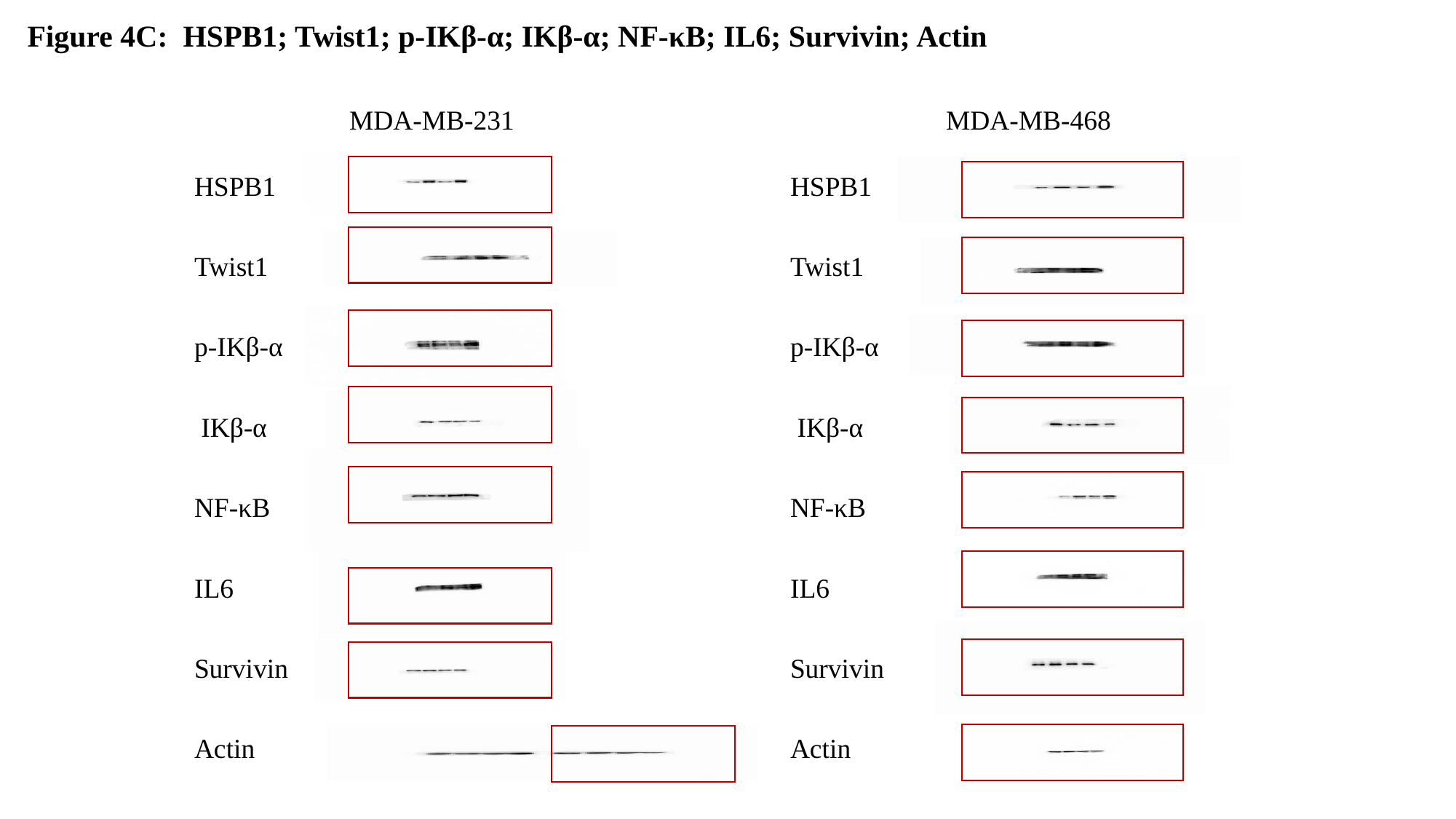

Figure 4C: HSPB1; Twist1; p-IKβ-α; IKβ-α; NF-κB; IL6; Survivin; Actin
MDA-MB-231
MDA-MB-468
HSPB1
HSPB1
Twist1
Twist1
p-IKβ-α
p-IKβ-α
 IKβ-α
 IKβ-α
NF-κB
NF-κB
IL6
IL6
Survivin
Survivin
Actin
Actin

## Slide 7
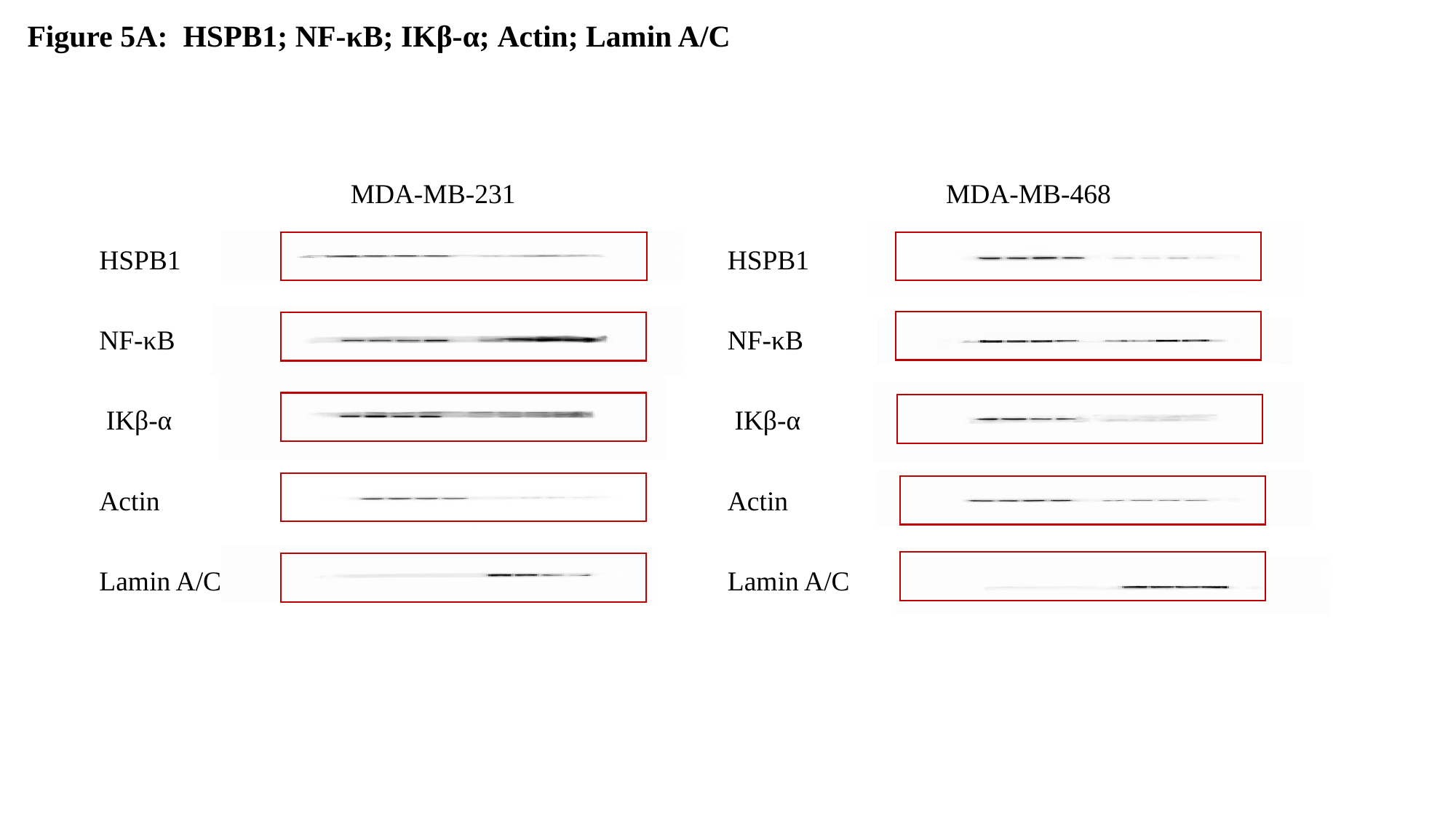

Figure 5A: HSPB1; NF-κB; IKβ-α; Actin; Lamin A/C
MDA-MB-231
MDA-MB-468
HSPB1
HSPB1
NF-κB
NF-κB
 IKβ-α
 IKβ-α
Actin
Actin
Lamin A/C
Lamin A/C

## Slide 8
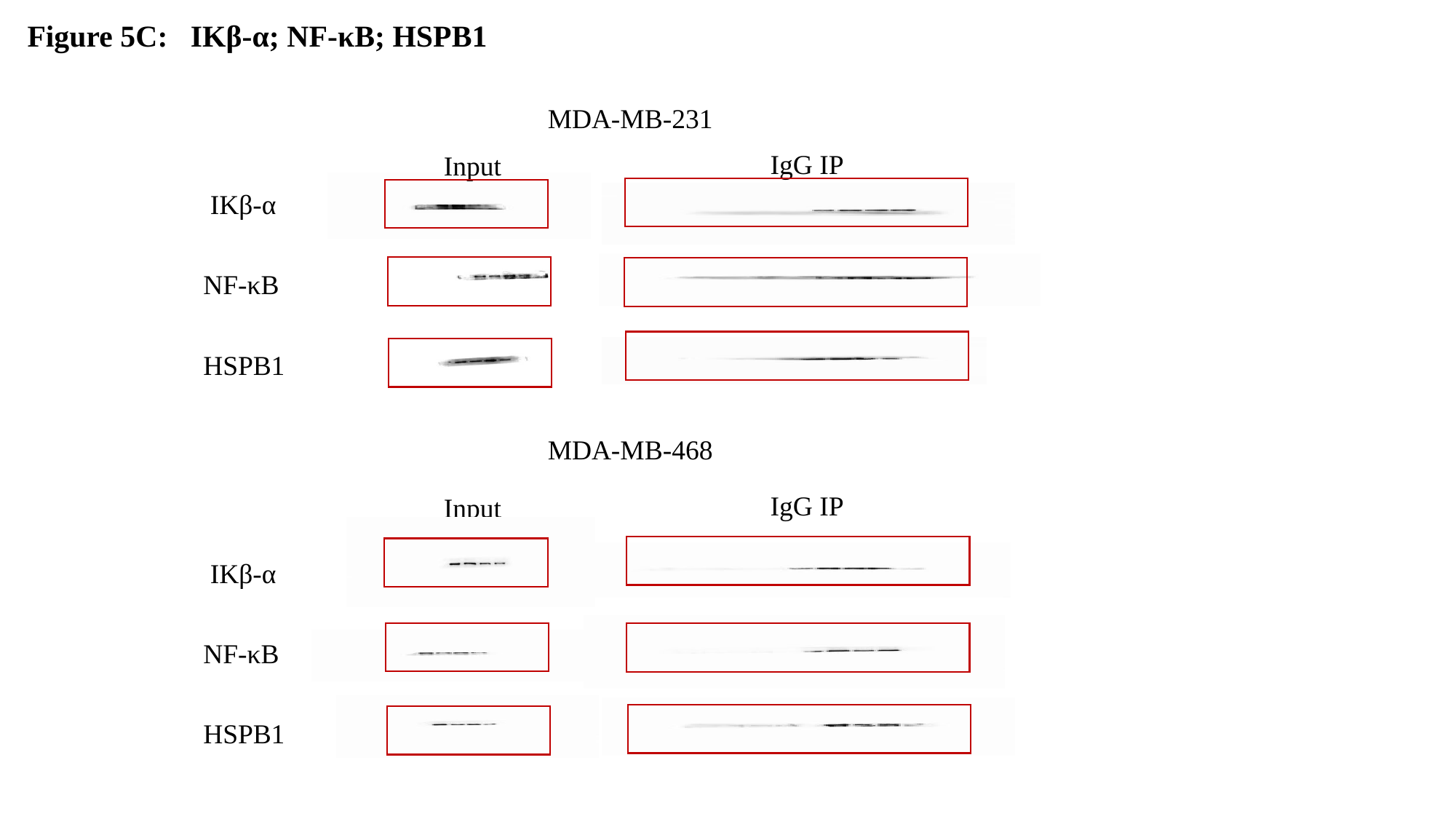

Figure 5C: IKβ-α; NF-κB; HSPB1
MDA-MB-231
IgG IP
Input
 IKβ-α
NF-κB
HSPB1
MDA-MB-468
IgG IP
Input
 IKβ-α
NF-κB
HSPB1

## Slide 9
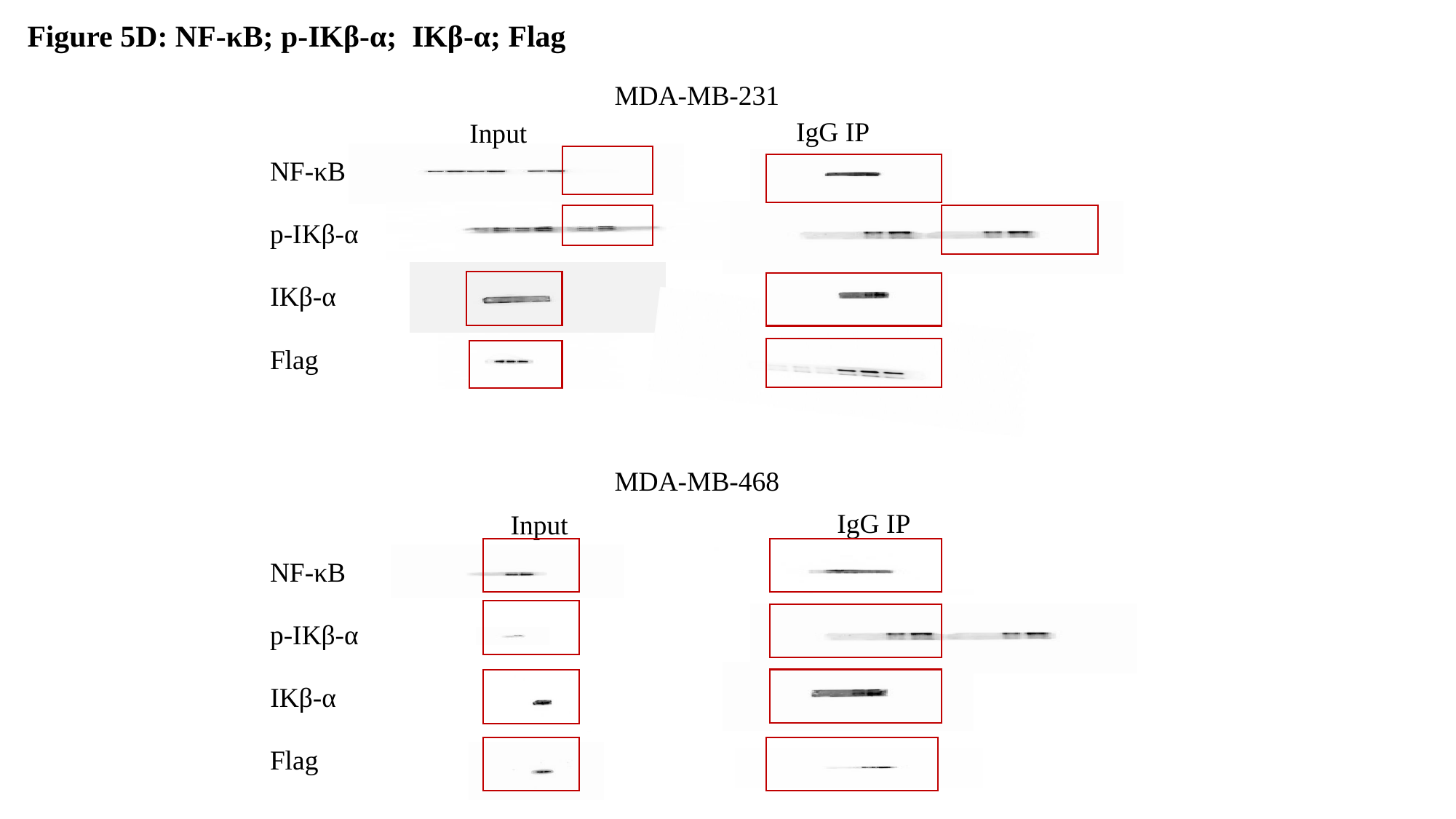

Figure 5D: NF-κB; p-IKβ-α; IKβ-α; Flag
MDA-MB-231
IgG IP
Input
NF-κB
p-IKβ-α
IKβ-α
Flag
MDA-MB-468
IgG IP
Input
NF-κB
p-IKβ-α
IKβ-α
Flag

## Slide 10
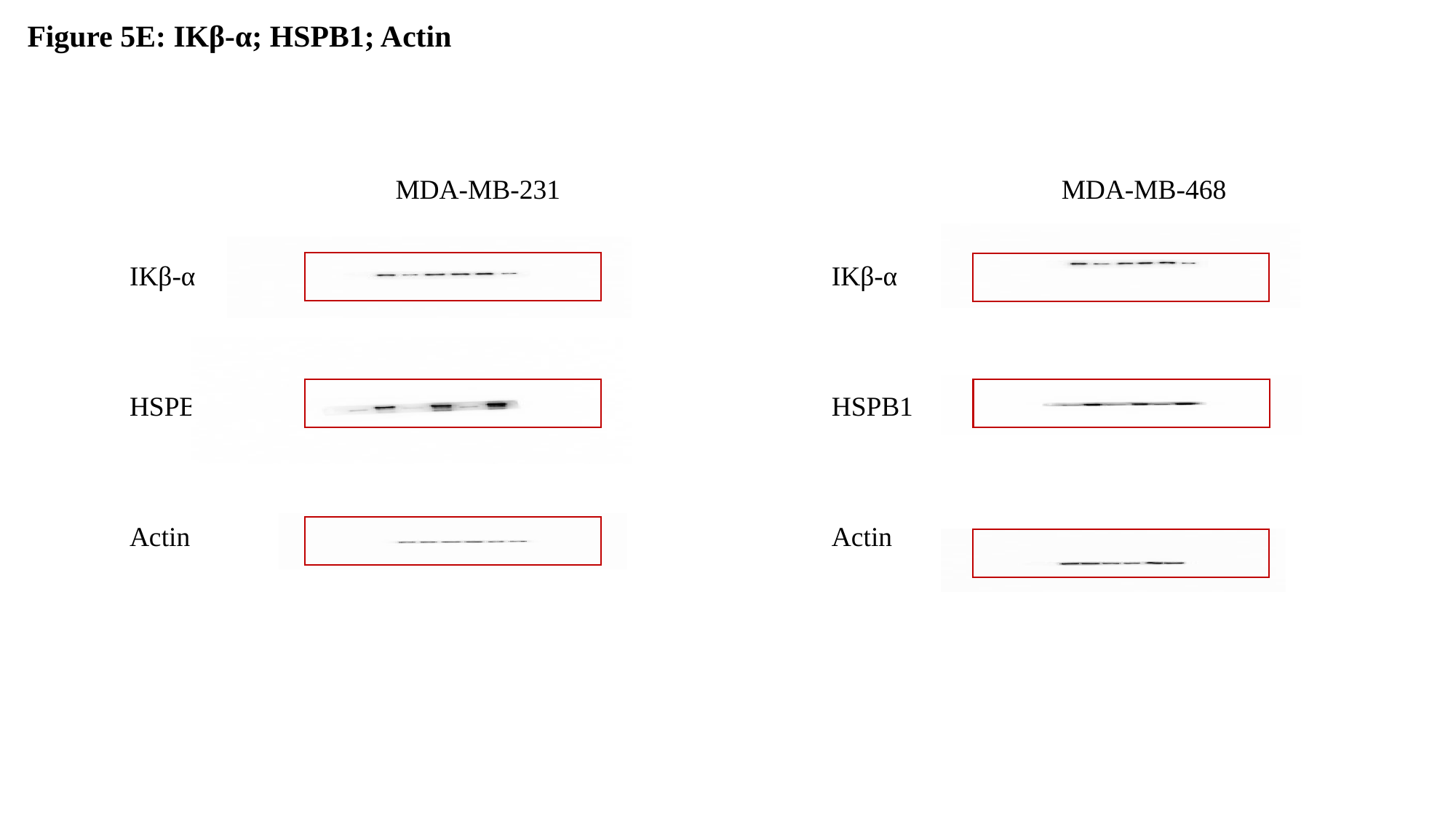

Figure 5E: IKβ-α; HSPB1; Actin
MDA-MB-231
MDA-MB-468
IKβ-α
IKβ-α
HSPB1
HSPB1
Actin
Actin

## Slide 11
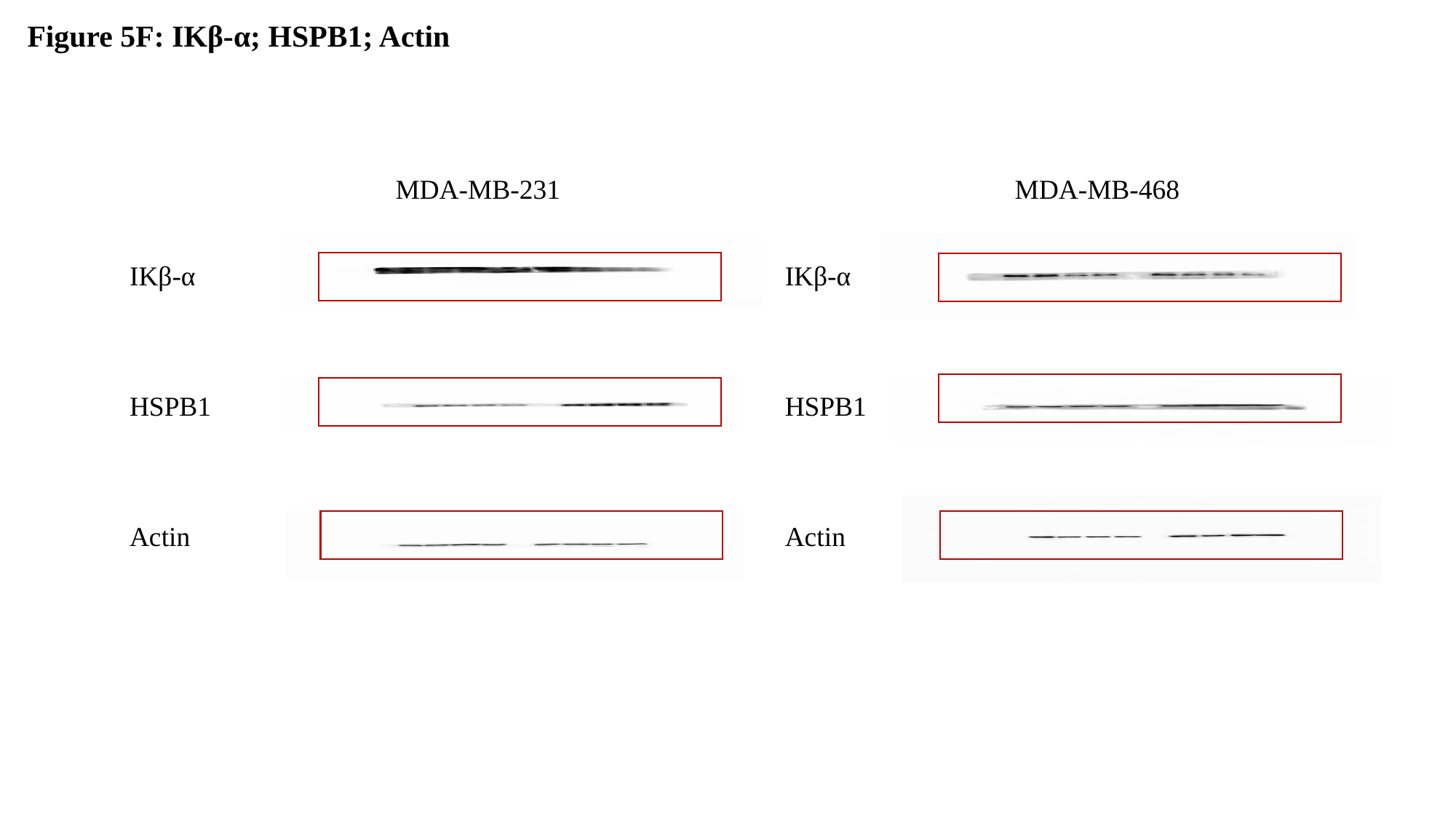

Figure 5F: IKβ-α; HSPB1; Actin
MDA-MB-231
MDA-MB-468
IKβ-α
IKβ-α
HSPB1
HSPB1
Actin
Actin

## Slide 12
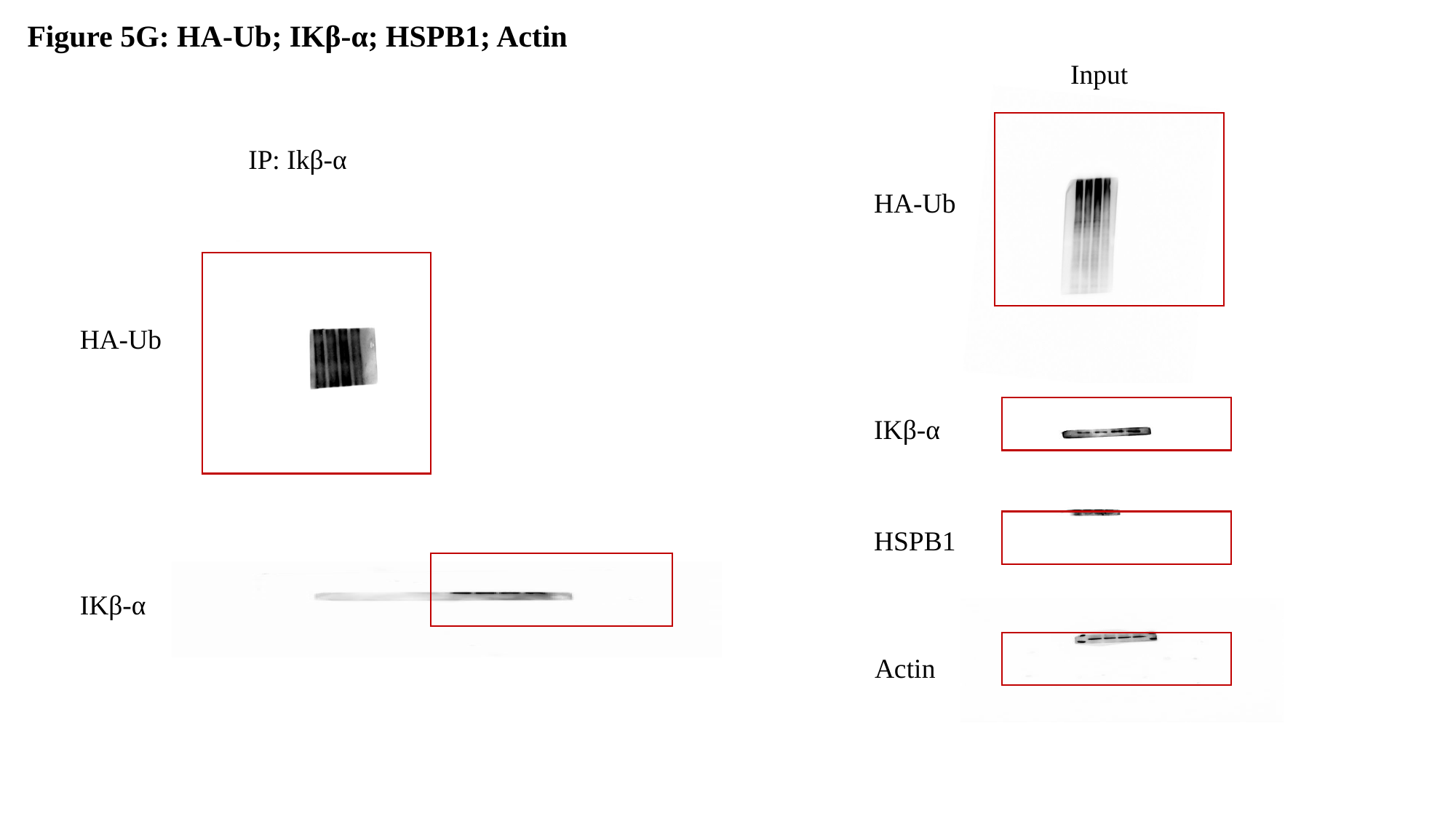

Figure 5G: HA-Ub; IKβ-α; HSPB1; Actin
Input
IP: Ikβ-α
HA-Ub
HA-Ub
IKβ-α
HSPB1
IKβ-α
Actin

## Slide 13
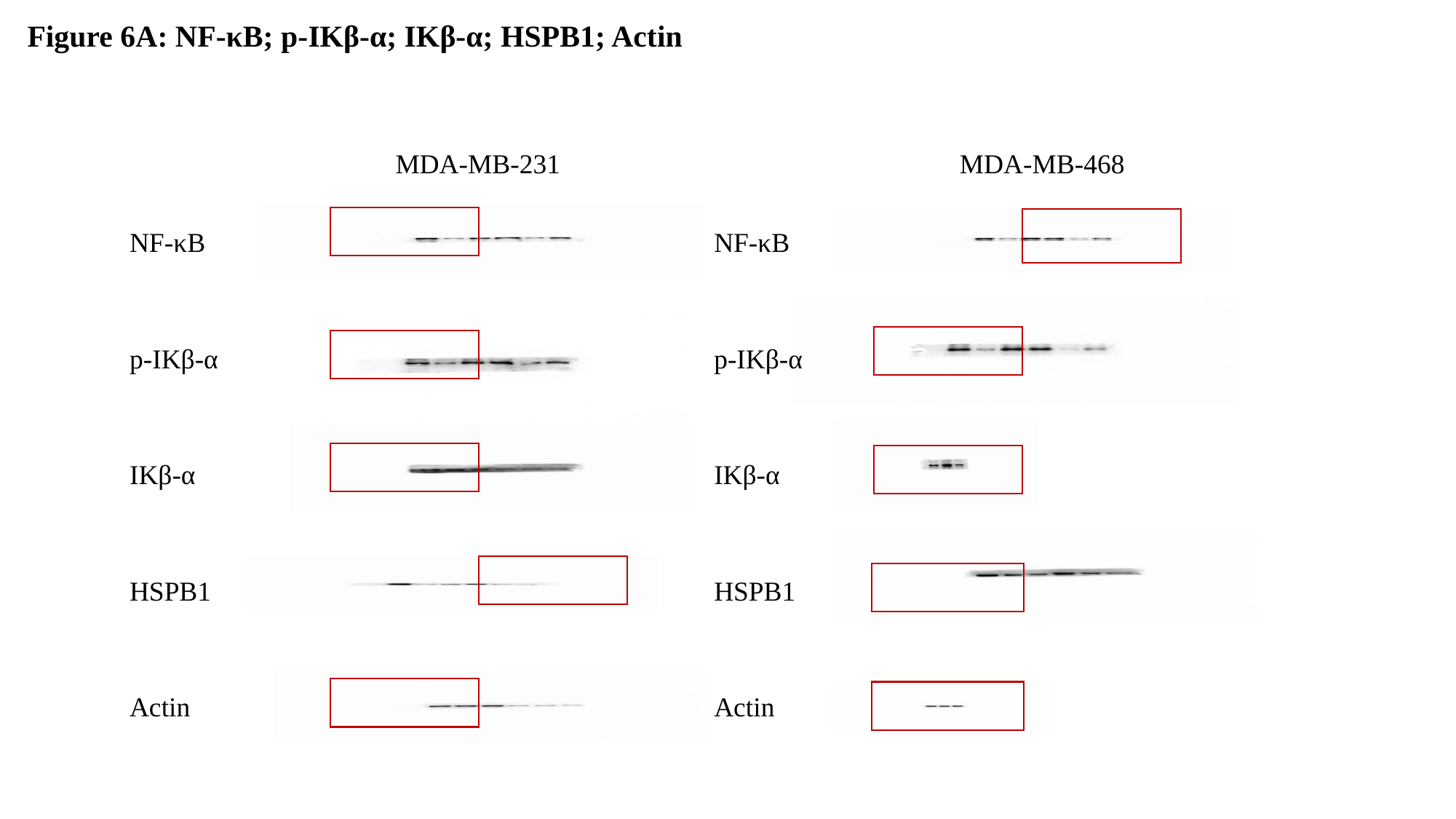

Figure 6A: NF-κB; p-IKβ-α; IKβ-α; HSPB1; Actin
MDA-MB-231
MDA-MB-468
NF-κB
NF-κB
p-IKβ-α
p-IKβ-α
IKβ-α
IKβ-α
HSPB1
HSPB1
Actin
Actin

## Slide 14
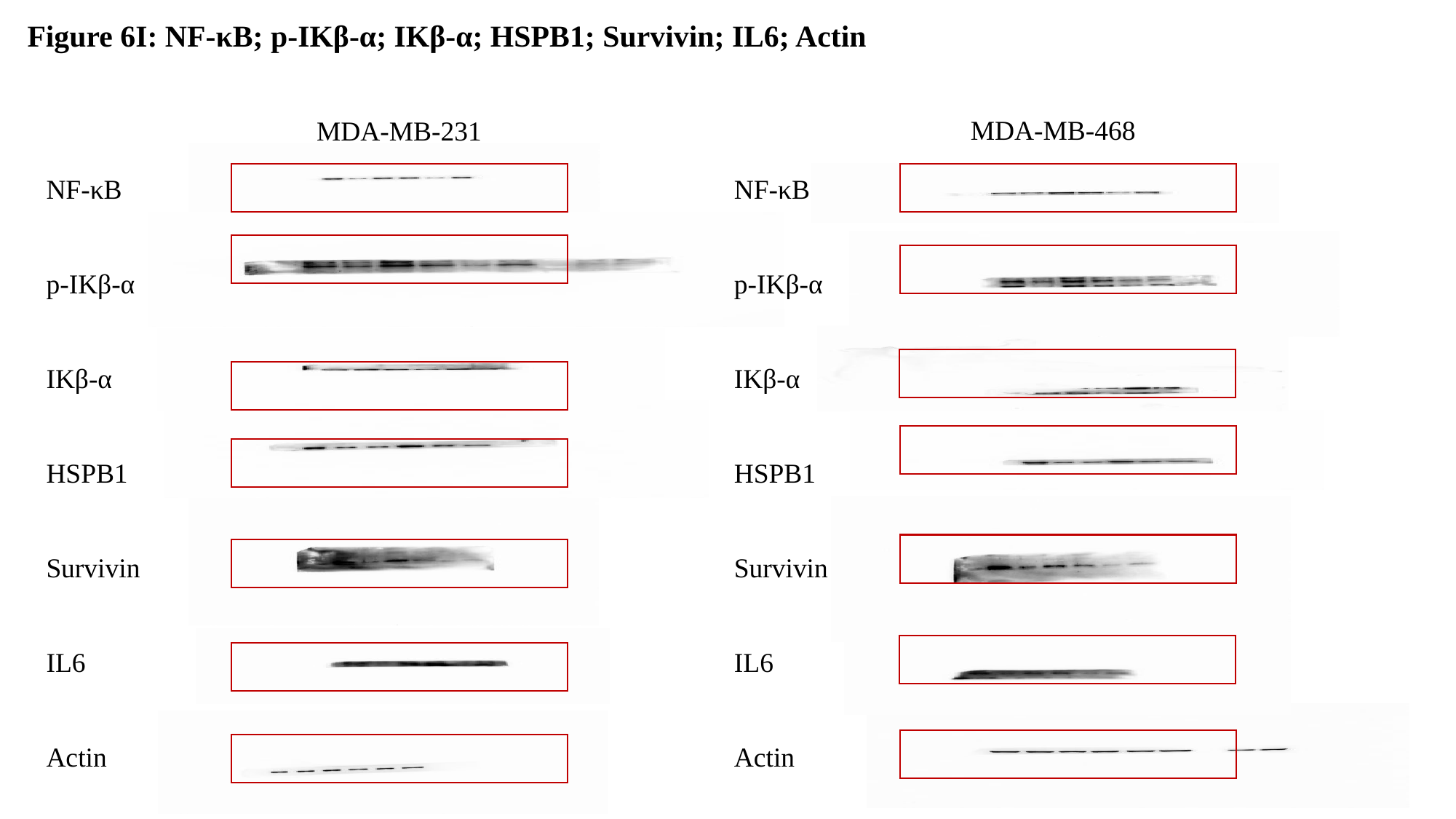

Figure 6I: NF-κB; p-IKβ-α; IKβ-α; HSPB1; Survivin; IL6; Actin
MDA-MB-468
MDA-MB-231
NF-κB
NF-κB
p-IKβ-α
p-IKβ-α
IKβ-α
IKβ-α
HSPB1
HSPB1
Survivin
Survivin
IL6
IL6
Actin
Actin

## Slide 15
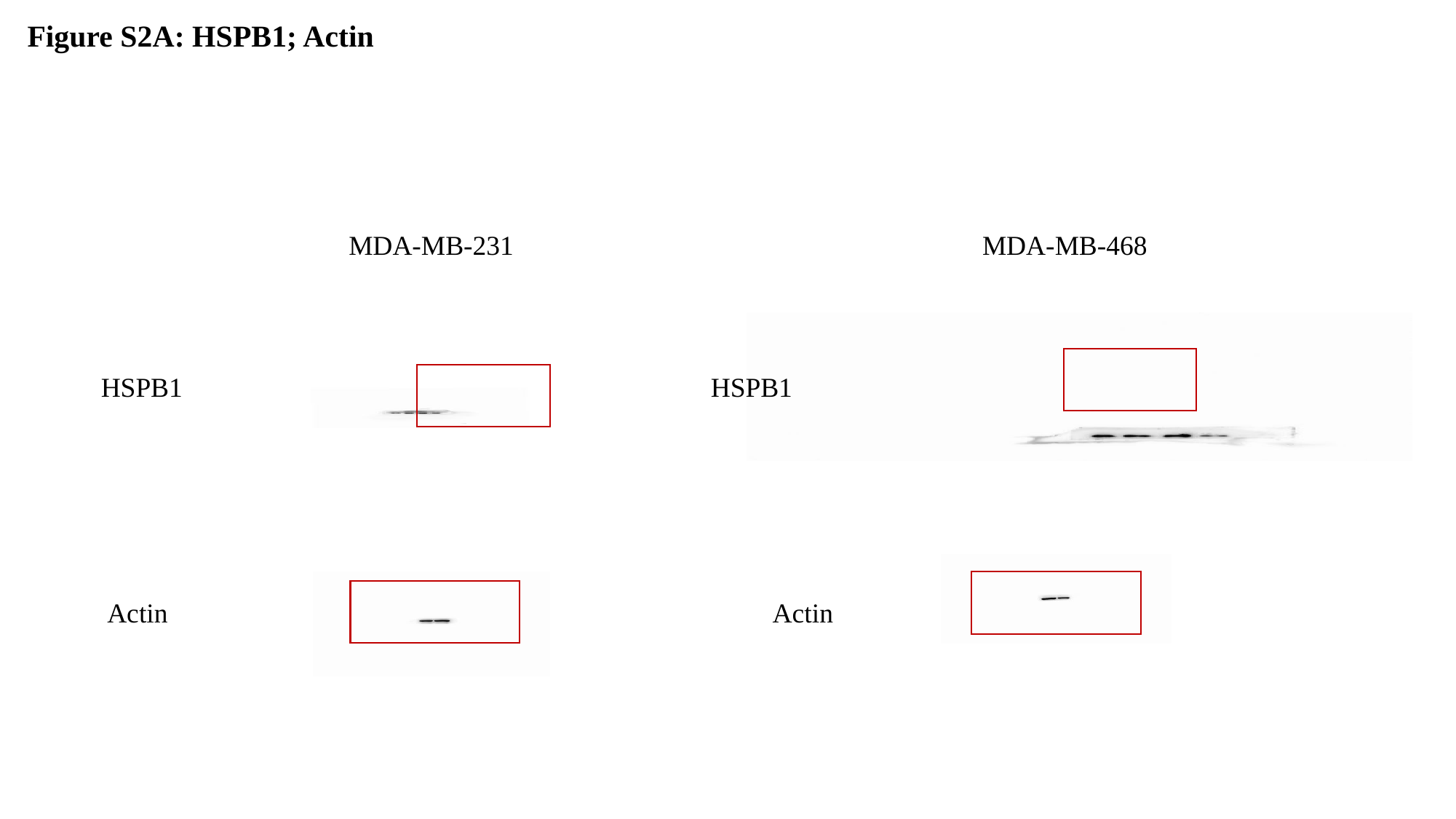

Figure S2A: HSPB1; Actin
MDA-MB-231
MDA-MB-468
HSPB1
HSPB1
Actin
Actin

## Slide 16
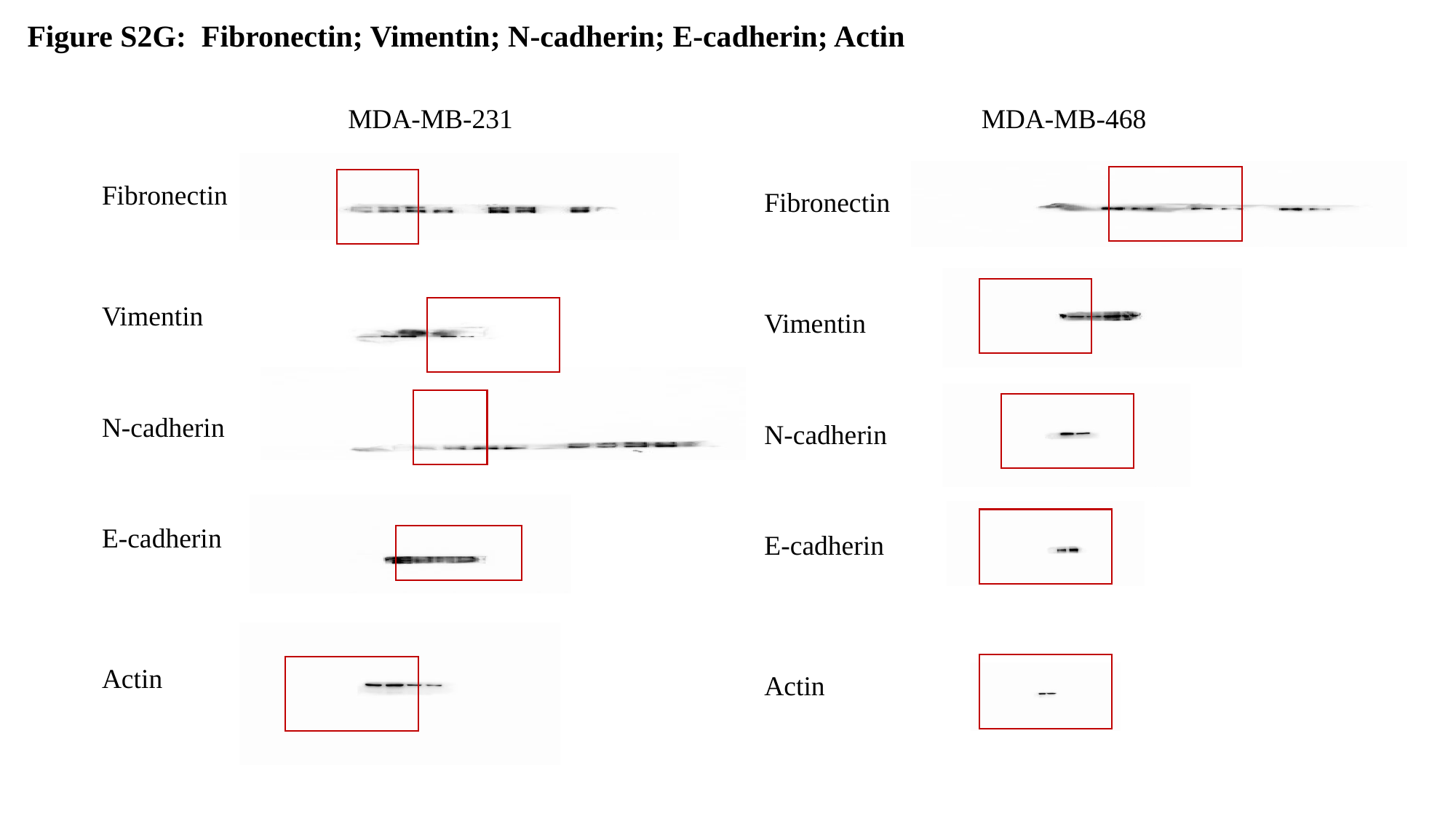

Figure S2G: Fibronectin; Vimentin; N-cadherin; E-cadherin; Actin
MDA-MB-231
MDA-MB-468
Fibronectin
Fibronectin
Vimentin
Vimentin
N-cadherin
N-cadherin
E-cadherin
E-cadherin
Actin
Actin

## Slide 17
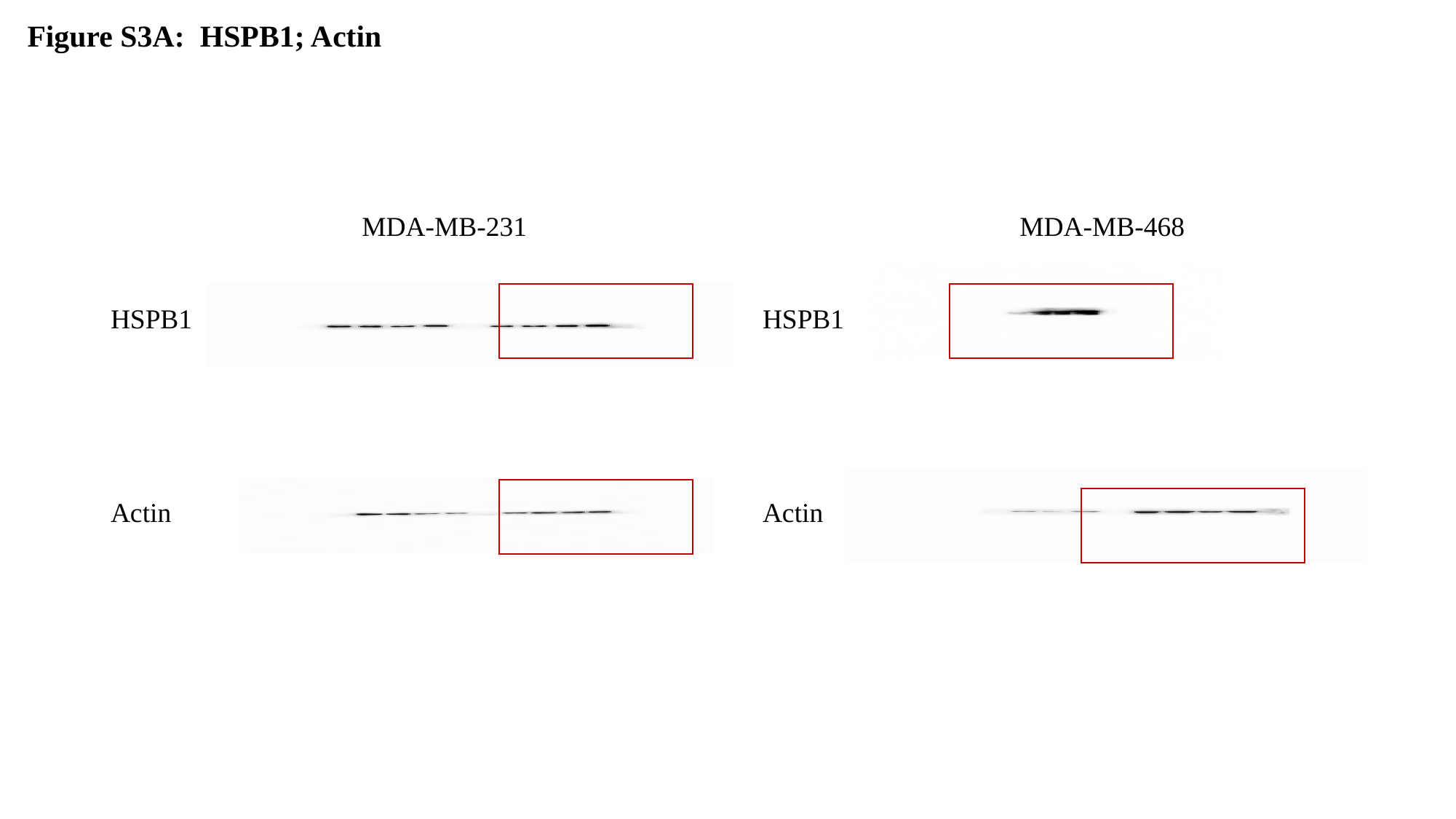

Figure S3A: HSPB1; Actin
MDA-MB-231
MDA-MB-468
HSPB1
HSPB1
Actin
Actin

## Slide 18
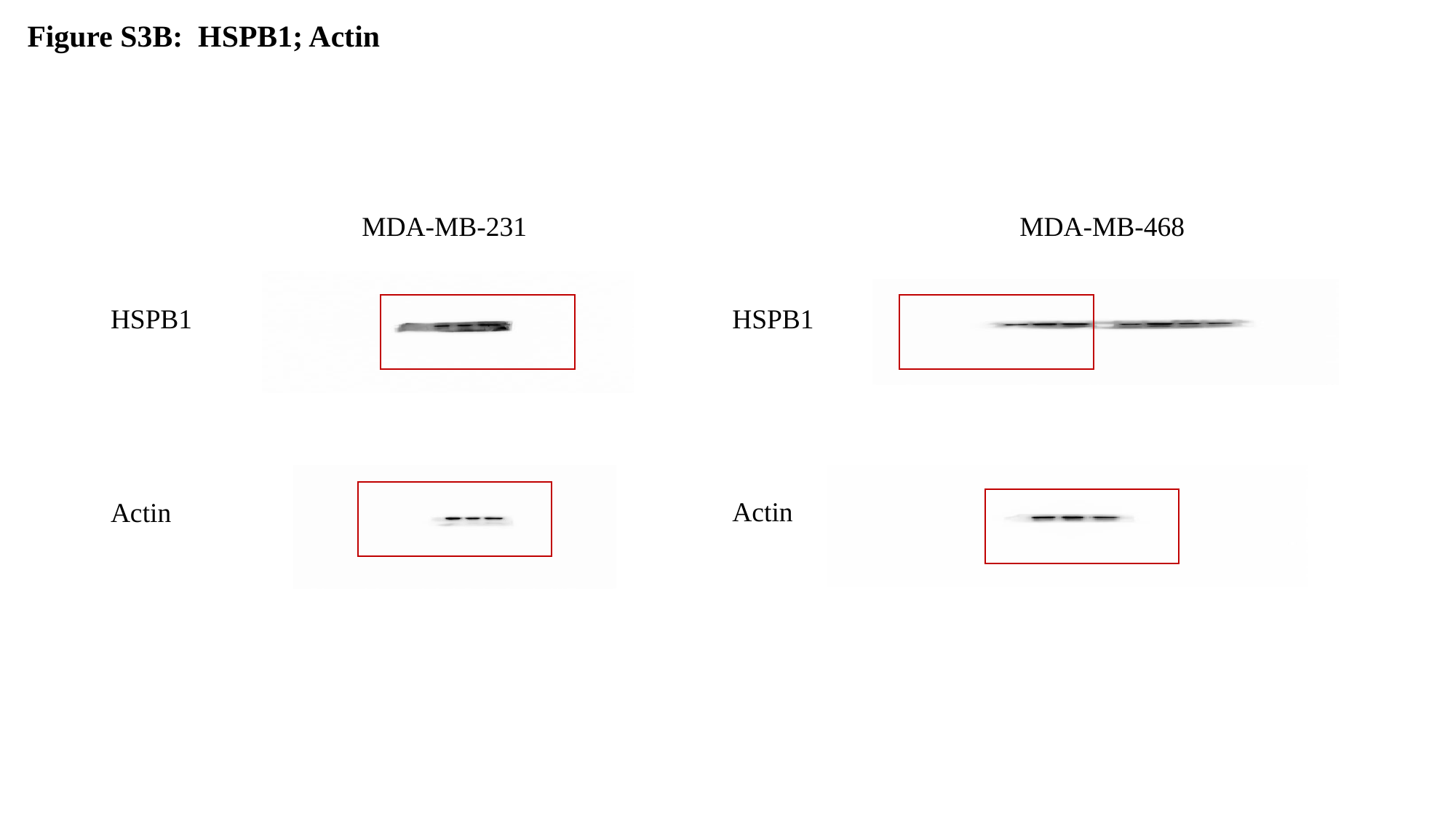

Figure S3B: HSPB1; Actin
MDA-MB-231
MDA-MB-468
HSPB1
HSPB1
Actin
Actin

## Slide 19
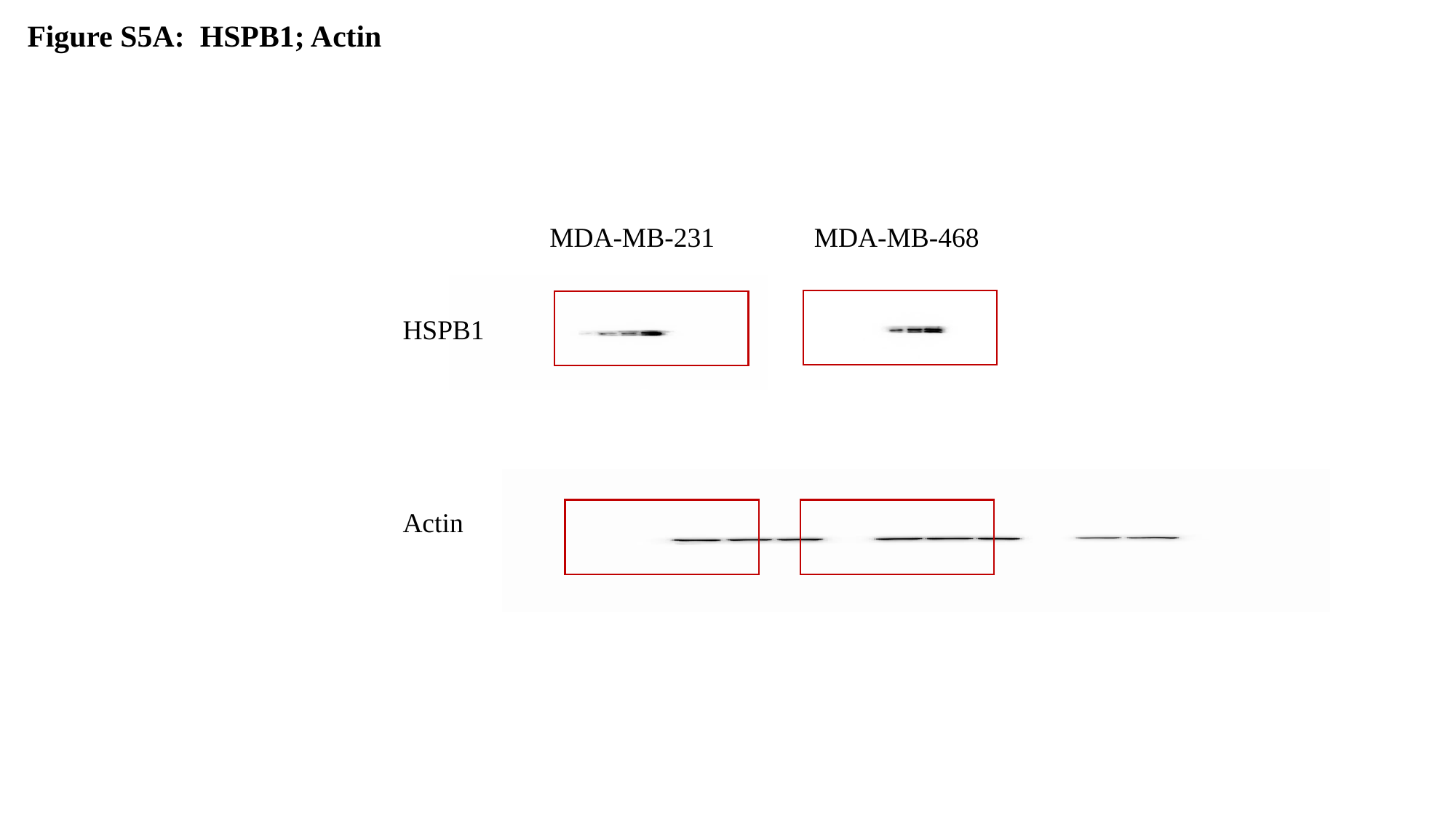

Figure S5A: HSPB1; Actin
MDA-MB-231
MDA-MB-468
HSPB1
Actin

## Slide 20
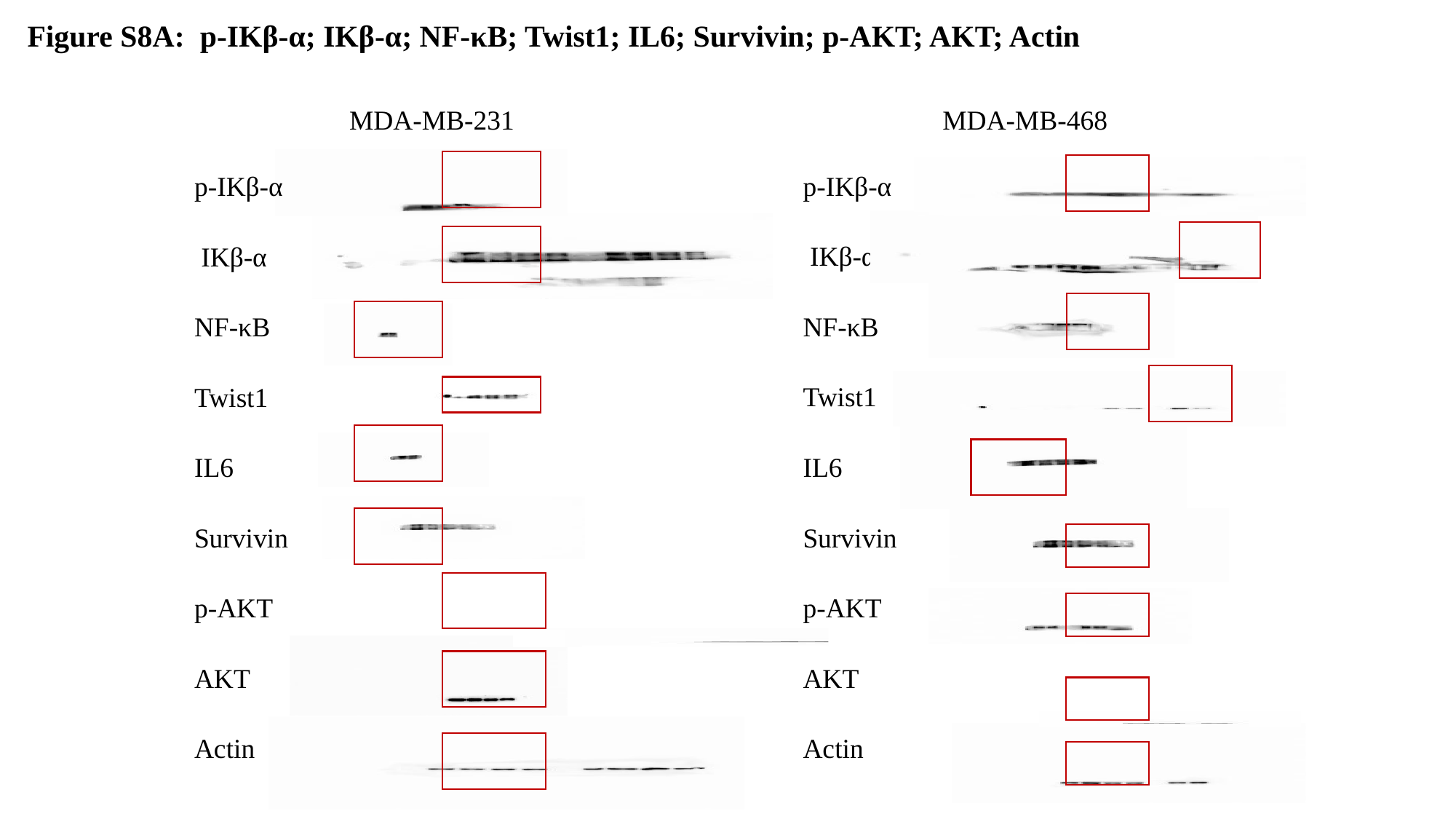

Figure S8A: p-IKβ-α; IKβ-α; NF-κB; Twist1; IL6; Survivin; p-AKT; AKT; Actin
MDA-MB-231
MDA-MB-468
p-IKβ-α
p-IKβ-α
 IKβ-α
 IKβ-α
NF-κB
NF-κB
Twist1
Twist1
IL6
IL6
Survivin
Survivin
p-AKT
p-AKT
AKT
AKT
Actin
Actin

## Slide 21
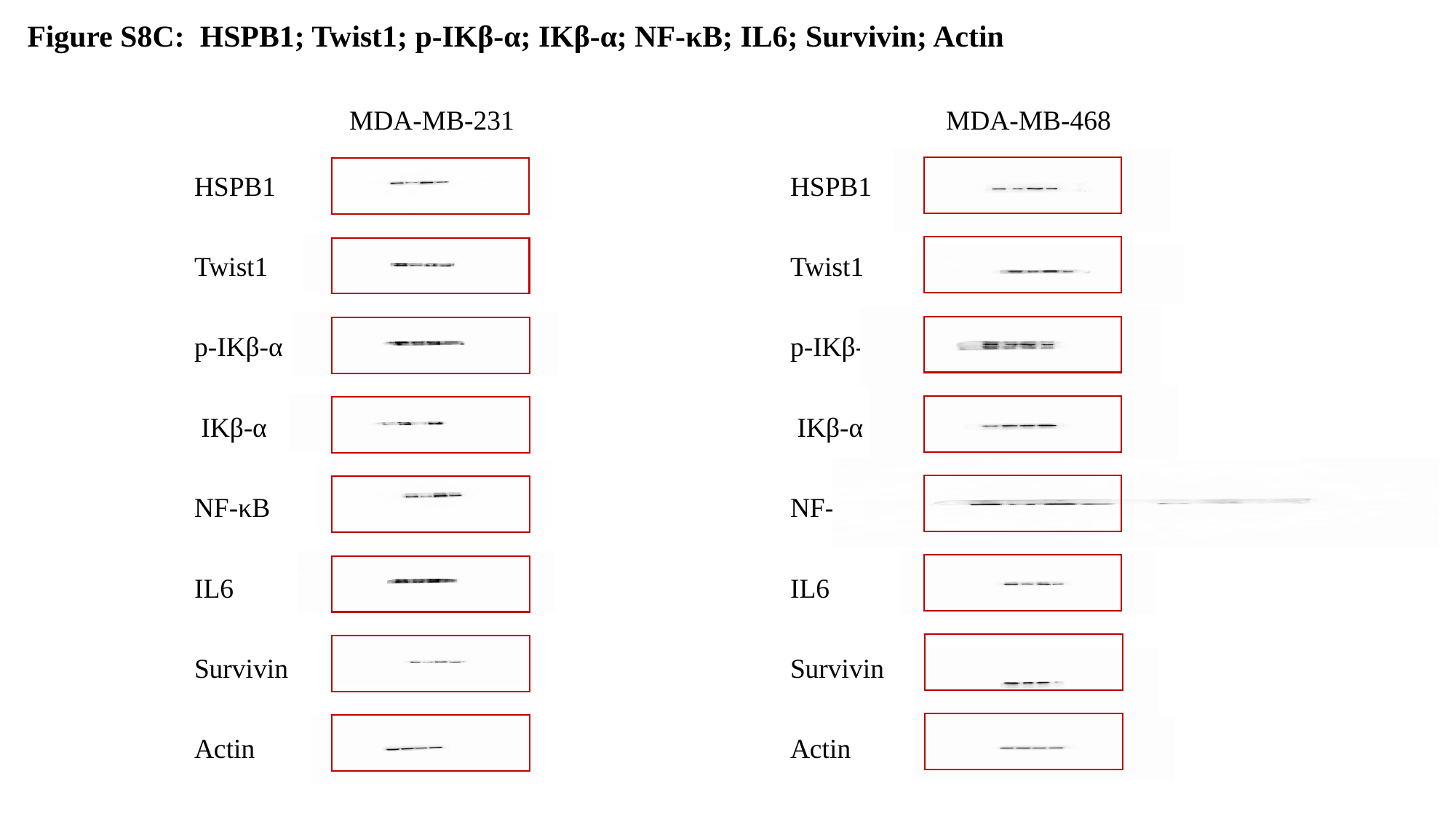

Figure S8C: HSPB1; Twist1; p-IKβ-α; IKβ-α; NF-κB; IL6; Survivin; Actin
MDA-MB-231
MDA-MB-468
HSPB1
HSPB1
Twist1
Twist1
p-IKβ-α
p-IKβ-α
 IKβ-α
 IKβ-α
NF-κB
NF-κB
IL6
IL6
Survivin
Survivin
Actin
Actin
